# Supplementary material for: Health effects associated with chewing tobacco: a Burden of Proof study
Source: Nat Commun. 2024 Feb 5;15:1082. doi: 10.1038/s41467-024-45074-9 (PMC10844244; doi:10.1038/s41467-024-45074-9)
Supplement: Supplementary file 1 — Supplementary Information [file 41467_2024_45074_MOESM1_ESM.pdf]

Supplementary Information for “Health effects associated with chewing tobacco: a Burden of Proof study"

This appendix provides detailed information on input data sources and supplementary results for the publication titled “Health effects associated with chewing tobacco: a Burden of Proof study.”

Table of Contents

Section 1: Data source identification and assessment 3

Section 1.1: Literature searches 3

Section 1.1.1: PubMed Search Strings 3

Section 1.1.2: Web of Science Search Strings 6

Section 1.1.3: Global Index Medicus Search Strings 8

Section 1.2: Inclusion and Exclusion Criteria 11

Section 1.3: Outcome Definitions 11

Table S1: Definitions of seven included outcomes 11

Section 1.4: Exposure Definition 12

Table S2: Local smokeless tobacco products categorized as chewing tobacco 12

Section 1.5: Data Extraction 13

Table S3: Data Extraction Template 13

Section 2: Data Inputs 19

Section 2.1: Study Characteristics 20

Table S4: Summary of Study Metadata and Characteristics 20

Section 2.2: Effect Size Data Details 36

Table S5: Summary of Data Inputs 37

Section 3: Supplementary Methods 50

Section 3.1: Umbrella review 50

Section 3.1.1: PubMed Umbrella Review Search String 50

Table S6: Outcome groupings of MA/SRs identified in the umbrella review 51

Section 3.2: The Scope of the Systematic Literature Reviews 51

Table S7: Data inputs for the relative risks of chewing tobacco use 52

Section 3.3: Study Quality and Bias Assessment 53

Table S8: Setting up the bias covariates to evaluate the level of adjustment of included data points 53

Table S9: Bias Characteristics for Included Observations 55

Table S10: Bias Covariates eligible for testing for each health outcome 68
Section 3.4: Detailed methods for estimating the relative risk of 8 health outcomes 69
Table S11: MR-BRT model specifications by risk-outcome pair 70
Section 3.5: Quantifying between-study heterogeneity 70
Table S12: Gamma solution for each risk-outcome pair quantifying between-study heterogeneity 70
Section 4: Sensitivity Analyses 70
Section 4.1: Sensitivity analyses for the relationship between chewing tobacco and stroke 72
Table S13: Results of sensitivity analyses for chewing tobacco and stroke 72
Section 4.2: Sensitivity analyses for the relationship between chewing tobacco and ischemic heart disease 73
Table S14: Results of sensitivity analyses for chewing tobacco and ischemic heart disease 73
Section 4.3: Sensitivity analyses for the relationship between chewing tobacco and esophageal cancer 74
Table S15: Results of sensitivity analyses for chewing tobacco and esophageal cancer 74
Section 4.4: Sensitivity analyses for the relationship between chewing tobacco and lip and oral cavity cancer 77
Table S16: Results of sensitivity analyses for chewing tobacco and lip and oral cavity cancer 77
Section 4.5: Sensitivity analyses for the relationship between chewing tobacco and laryngeal cancer 80
Table S17: Results of sensitivity analyses for chewing tobacco and laryngeal cancer 80
Section 4.6: Sensitivity analyses for the relationship between chewing tobacco and nasopharynx cancer 82
Table S18: Results of sensitivity analyses for chewing tobacco and nasopharynx cancer 82
Section 4.7: Sensitivity analyses for the relationship between chewing tobacco and other pharynx cancer 84
Table S19: Results of sensitivity analyses for chewing tobacco and other pharynx cancer 84
Section 4.8: Study characteristics and observations for sex-specific sensitivity analyses 87
Table S20: Study characteristics for male-specific sensitivity analysis 87
Table S21: Study characteristics for female-specific sensitivity analysis 88
Table S22: Additional data inputs for sex-specific sensitivity analyses 88
Section 5: GATHER and PRISMA checklists 90
Section 5.1: PRISMA 90
Table S23: PRISMA 2020 Abstract Checklist 90
Table S24: PRISMA 2020 Manuscript Checklist 91
Figure S1. PRISMA 2020 flow diagram for chewing tobacco and head and neck cancers 94

Figure S2. PRISMA 2020 flow diagram for chewing tobacco and ischemic heart disease.................95
Figure S3. PRISMA 2020 flow diagram for chewing tobacco and stroke..96
Section 5.2: GATHER 97
Table S25: GATHER Checklist 97
Section 6: Supplementary References 98

Section 1: Data source identification and assessment

Data for the present analysis was identified through three systematic reviews for our seven health outcomes of interest. Each data source underwent a minimum of two independent screenings and evaluated on the basis of pre-established inclusion and exclusion criteria described below.

Section 1.1: Literature searches

We conducted three systematic reviews for head and neck cancers, ischemic heart disease, and stroke, respectively. These reviews encompassed three databases: PubMed, Web of Science, and Global Index Medicus. These databases were selected to cover a broad swath of peer-reviewed global and regional literature. The search strings were last run on February 15, 2023 to capture all studies, irrespective of publication language, published from January 1, 1970 through January 30, 2023.

Section 1.1.1: PubMed Search Strings

Head and Neck Cancers

("smokeless tobacco"[Title/Abstract] OR "Tobacco, Smokeless"[MeSH Terms] OR bajjar[Title/Abstract] OR ("betel quid"[Title/Abstract] AND tobacco[Title/Abstract]) OR "chewing tobacco"[Title/Abstract] OR chimó[Title/Abstract] OR snuff[Title/Abstract] OR snuif[Title/Abstract] OR dip[Title/Abstract] OR dohra[Title/Abstract] OR gudakhu[Title/Abstract] OR gul[Title/Abstract] OR gutka[Title/Abstract] OR gutkha[Title/Abstract] OR "hnat hsey"[Title/Abstract] OR iq'mik[Title/Abstract] OR khaini[Title/Abstract] OR kharra[Title/Abstract] OR khiwam[Title/Abstract] OR khimam[Title/Abstract] OR kiwam[Title/Abstract] OR kimam[Title/Abstract] OR "lal dant manjan"[Title/Abstract] OR ("loose leaf"[Title/Abstract] AND (chew[Title/Abstract] OR tobacco[Title/Abstract])) OR mainpuri[Title/Abstract] OR maras[Title/Abstract] OR mawa[Title/Abstract] OR mshri[Title/Abstract] OR naffa[Title/Abstract] OR nas[Supplementary Concept] OR ((nas[Title/Abstract] OR nass[Title/Abstract]) AND tobacco[Title/Abstract]) OR naswar[Title/Abstract] OR nasway[Title/Abstract] OR nasvay[Title/Abstract] OR neffa[Title/Abstract] OR((pan[Title/Abstract] OR paan[Title/Abstract]) AND tobacco[Title/Abstract]) OR (plug[Title/Abstract] AND tobacco[Title/Abstract]) OR (rapé[Title/Abstract] AND tobacco[Title/Abstract]) OR ((red[Title/Abstract] OR tobacco[Title/Abstract]) AND (toothpowder[Title/Abstract] OR toothpaste[Title/Abstract])) OR shammah[Title/Abstract] OR snus[Title/Abstract] OR taaba[Title/Abstract] OR tapkeer[Title/Abstract] OR tawa[Title/Abstract] OR tombol[Title/Abstract] OR toombak[Title/Abstract] OR tuibur[Title/Abstract] OR "tobacco water"[Title/Abstract] OR (twist[Title/Abstract] AND tobacco[Title/Abstract]) OR zarda[Title/Abstract]) AND

(Head and neck neoplasms[MeSH Terms] OR "head and neck cancers"[Title/Abstract] OR "head and neck neoplasms"[Title/Abstract] OR "pharyngeal cancer"[Title/Abstract] OR "pharyngeal cancers"[Title/Abstract] OR "pharyngeal neoplasm"[Title/Abstract] OR "pharyngeal

neoplasms"[Title/Abstract] OR "pharynx cancer"[Title/Abstract] OR "pharynx cancers"[Title/Abstract] OR "pharynx neoplasm"[Title/Abstract] OR "pharynx neoplasms"[Title/Abstract] OR "cancer of the pharynx"[Title/Abstract] OR "cancers of the pharynx"[Title/Abstract] OR "nasopharyngeal cancer"[Title/Abstract] OR "nasopharyngeal cancers"[Title/Abstract] OR "nasopharyngeal neoplasm"[Title/Abstract] OR "nasopharyngeal neoplasms"[Title/Abstract] OR "nasopharynx cancer"[Title/Abstract] OR "nasopharynx cancers"[Title/Abstract] OR "nasopharynx neoplasm"[Title/Abstract] OR "nasopharynx neoplasms"[Title/Abstract] OR "cancer of the nasopharynx"[Title/Abstract] OR "cancers of the nasopharynx"[Title/Abstract] OR "neoplasm of the nasopharynx"[Title/Abstract] OR "neoplasms of the nasopharynx"[Title/Abstract] OR "mouth cancer"[Title/Abstract] OR "mouth cancers"[Title/Abstract] OR "mouth neoplasm"[Title/Abstract] OR "mouth neoplasms"[Title/Abstract] OR "cancer of mouth"[Title/Abstract] OR "cancer of the mouth"[Title/Abstract] OR "cancers of the mouth"[Title/Abstract] OR "oral cancer"[Title/Abstract] OR "oral cancers"[Title/Abstract] OR "oral neoplasm"[Title/Abstract] OR "oral neoplasms"[Title/Abstract] OR "gingival neoplasm"[Title/Abstract] OR "gingival neoplasms"[Title/Abstract] OR "gingival cancer"[Title/Abstract] OR "gingival cancers"[Title/Abstract] OR "lip neoplasm"[Title/Abstract] OR "lip neoplasms"[Title/Abstract] OR "lip cancer"[Title/Abstract] OR "lip cancers"[Title/Abstract] OR "cancer of the lip"[Title/Abstract] OR "cancers of the lip"[Title/Abstract] OR "tongue neoplasm"[Title/Abstract] OR "tongue neoplasms"[Title/Abstract] OR "tongue cancer"[Title/Abstract] OR "tongue cancers"[Title/Abstract] OR "cancer of the tongue"[Title/Abstract] OR "cancers of the tongue"[Title/Abstract] OR "palatal neoplasm"[Title/Abstract] OR "palatal neoplasms"[Title/Abstract] OR "palatal cancer"[Title/Abstract] OR "palatal cancers"[Title/Abstract] OR "oral cavity cancer"[Title/Abstract] OR "oral cavity cancers"[Title/Abstract] OR "laryngeal cancer"[Title/Abstract] OR "laryngeal cancers"[Title/Abstract] OR "laryngeal neoplasm"[Title/Abstract] OR "laryngeal neoplasms"[Title/Abstract] OR "larynx cancer"[Title/Abstract] OR "larynx cancers"[Title/Abstract] OR "larynx neoplasm"[Title/Abstract] OR "larynx neoplasms"[Title/Abstract] OR "cancer of the larynx"[Title/Abstract] OR "cancers of the larynx"[Title/Abstract] OR "neoplasm of the larynx"[Title/Abstract] OR "neoplasms of the larynx"[Title/Abstract] OR "esophageal cancer"[Title/Abstract] OR "esophageal cancers"[Title/Abstract] OR "esophageal neoplasm"[Title/Abstract] OR "esophageal neoplasms"[Title/Abstract] OR "esophagus cancer"[Title/Abstract] OR "esophagus cancers"[Title/Abstract] OR "esophagus neoplasm"[Title/Abstract] OR "esophagus neoplasms"[Title/Abstract] OR "esophageal squamous cell carcinoma"[Title/Abstract] OR "cancer of the esophagus"[Title/Abstract] OR "cancers of the esophagus"[Title/Abstract] OR "neoplasm of the esophagus"[Title/Abstract] OR "neoplasms of the esophagus"[Title/Abstract] OR "oesophageal cancer"[Title/Abstract] OR "oesophageal cancers"[Title/Abstract] OR "oesophageal neoplasm"[Title/Abstract]OR "oesophageal neoplasms"[Title/Abstract] OR "oesophagus cancer"[Title/Abstract] OR "oesophagus cancers"[Title/Abstract] OR "oesophagus neoplasm"[Title/Abstract] OR "oesophagus neoplasms"[Title/Abstract] OR "oesophageal squamous cell carcinoma"[Title/Abstract] OR "cancer of the oesophagus"[Title/Abstract] OR "cancers of the oesophagus"[Title/Abstract] OR "neoplasm of the oesophagus"[Title/Abstract] OR "neoplasms of the oesophagus"[Title/Abstract] OR "oropharyngeal cancer"[Title/Abstract] OR "oropharyngeal cancers"[Title/Abstract] OR "oropharynx cancer"[Title/Abstract] OR "oropharynx cancers"[Title/Abstract] OR "oropharyngeal neoplasm"[Title/Abstract] OR "oropharyngeal neoplasms"[Title/Abstract] OR "oropharynx neoplasm"[Title/Abstract] OR "cancer of the oropharynx"[Title/Abstract] OR "cancers of the oropharynx"[Title/Abstract] OR "neoplasm of the oropharynx"[Title/Abstract] OR "neoplasms of the oropharynx"[Title/Abstract] OR "hypopharyngeal cancer"[Title/Abstract] OR "hypopharyngeal cancers"[Title/Abstract] OR "hypopharyngeal neoplasm"[Title/Abstract] OR "hypopharyngeal

neoplasms"[Title/Abstract] OR "cancer of the hypopharynx"[Title/Abstract] OR "cancers of the hypopharynx"[Title/Abstract] OR "neoplasm of the hypopharynx"[Title/Abstract] OR "neoplasms of the hypopharynx"[Title/Abstract]) AND

(Risk[MeSH Terms] OR Odds Ratio[MeSH Terms] OR "risk"[Title/Abstract] OR "odds ratio"[Title/Abstract] OR "cross-product ratio"[Title/Abstract] OR "hazards ratio"[Title/Abstract] OR "hazard ratio"[Title/Abstract]) AND ("1970/01/01"[PDat] : "2023/01/30"[PDat]) NOT (animals[MeSH Terms] NOT Humans[MeSH Terms])

Ischemic Heart Disease

("smokeless tobacco"[Title/Abstract] OR "Tobacco, Smokeless"[MeSH Terms] OR bajjar[Title/Abstract] OR ("betel quid"[Title/Abstract] AND tobacco[Title/Abstract]) OR "chewing tobacco"[Title/Abstract] OR chimó[Title/Abstract] OR snuff[Title/Abstract] OR snuif[Title/Abstract] OR dip[Title/Abstract] OR dohra[Title/Abstract] OR gudakhu[Title/Abstract] OR gul[Title/Abstract] OR gutka[Title/Abstract] OR gutkha[Title/Abstract] OR "hnat hsey"[Title/Abstract] OR iq'mik[Title/Abstract] OR khaini[Title/Abstract] OR kharra[Title/Abstract] OR khiwam[Title/Abstract] OR khimam[Title/Abstract] OR kiwam[Title/Abstract] OR kimam[Title/Abstract] OR "lal dant manjan"[Title/Abstract] OR ("loose leaf"[Title/Abstract] AND (chew[Title/Abstract] OR tobacco[Title/Abstract])) OR mainpuri[Title/Abstract] OR maras[Title/Abstract] OR mawa[Title/Abstract] OR mshri[Title/Abstract] OR naffa[Title/Abstract] OR nas[Supplementary Concept] OR ((nas[Title/Abstract] OR nass[Title/Abstract]) AND tobacco[Title/Abstract]) OR naswar[Title/Abstract] OR nasway[Title/Abstract] OR nasvay[Title/Abstract] OR neffa[Title/Abstract] OR((pan[Title/Abstract] OR paan[Title/Abstract]) AND tobacco[Title/Abstract]) OR (plug[Title/Abstract] AND tobacco[Title/Abstract]) OR (rapé[Title/Abstract] AND tobacco[Title/Abstract]) OR ((red[Title/Abstract] OR tobacco[Title/Abstract]) AND (toothpowder[Title/Abstract] OR toothpaste[Title/Abstract])) OR shammah[Title/Abstract] OR snus[Title/Abstract] OR taaba[Title/Abstract] OR tapkeer[Title/Abstract] OR tawa[Title/Abstract] OR tombol[Title/Abstract] OR toombak[Title/Abstract] OR tuibur[Title/Abstract] OR "tobacco water"[Title/Abstract] OR (twist[Title/Abstract] AND tobacco[Title/Abstract]) OR zarda[Title/Abstract]) AND

("Myocardial Ischemia"[MeSH Terms] OR "heart disease"[Title/Abstract] OR "coronary"[Title/Abstract] OR"myocardial infarction"[Title/Abstract] OR "heart attack"[Title/Abstract] OR "heart disease"[Title/Abstract]) AND

(Risk[MeSH Terms] OR Odds Ratio[MeSH Terms] OR "risk"[Title/Abstract] OR "odds ratio"[Title/Abstract] OR "cross-product ratio"[Title/Abstract] OR "hazards ratio"[Title/Abstract] OR "hazard ratio"[Title/Abstract]) AND ("1970/01/01"[PDat] : "2023/01/30"[PDat]) NOT (animals[MeSH Terms] NOT Humans[MeSH Terms])

Stroke

("smokeless tobacco"[Title/Abstract] OR "Tobacco, Smokeless"[MeSH Terms] OR bajjar[Title/Abstract] OR ("betel quid"[Title/Abstract] AND tobacco[Title/Abstract]) OR "chewing tobacco"[Title/Abstract] OR chimó[Title/Abstract] OR snuff[Title/Abstract] OR snuif[Title/Abstract] OR dip[Title/Abstract] OR dohra[Title/Abstract] OR gudakhu[Title/Abstract] OR gul[Title/Abstract] OR gutka[Title/Abstract] OR gutkha[Title/Abstract] OR "hnat hsey"[Title/Abstract] OR iq'mik[Title/Abstract] OR khaini[Title/Abstract] OR kharra[Title/Abstract] OR khiwam[Title/Abstract] OR khimam[Title/Abstract] OR kiwam[Title/Abstract] OR kimam[Title/Abstract] OR "lal dant manjan"[Title/Abstract] OR ("loose leaf"[Title/Abstract] AND (chew[Title/Abstract] OR tobacco[Title/Abstract])) OR

mainpuri[Title/Abstract] OR maras[Title/Abstract] OR mawa[Title/Abstract] OR mshri[Title/Abstract] OR naffa[Title/Abstract] OR nas[Supplementary Concept] OR ((nas[Title/Abstract] OR nass[Title/Abstract]) AND tobacco[Title/Abstract]) OR naswar[Title/Abstract] OR nasway[Title/Abstract] OR nasvay[Title/Abstract] OR neffa[Title/Abstract] OR((pan[Title/Abstract] OR paan[Title/Abstract]) AND tobacco[Title/Abstract]) OR (plug[Title/Abstract] AND tobacco[Title/Abstract]) OR (rapé[Title/Abstract] AND tobacco[Title/Abstract]) OR ((red[Title/Abstract] OR tobacco[Title/Abstract]) AND (toothpowder[Title/Abstract] OR toothpaste[Title/Abstract])) OR shammah[Title/Abstract] OR snus[Title/Abstract] OR taaba[Title/Abstract] OR tapkeer[Title/Abstract] OR tawa[Title/Abstract] OR tombol[Title/Abstract] OR toombak[Title/Abstract] OR tuibur[Title/Abstract] OR "tobacco water"[Title/Abstract] OR (twist[Title/Abstract] AND tobacco[Title/Abstract]) OR zarda[Title/Abstract]) AND

("Stroke"[MeSH Terms] OR "stroke"[Title/Abstract] OR "cva"[Title/Abstract] OR "cerebrovascular"[Title/Abstract] OR "hemorrhage"[Title/Abstract] OR "haemorrhage"[Title/Abstract] OR "aneurism"[Title/Abstract] OR "aneurysm"[Title/Abstract]) AND

(Risk[MeSH Terms] OR Odds Ratio[MeSH Terms] OR "risk"[Title/Abstract] OR "odds ratio"[Title/Abstract] OR "cross-product ratio"[Title/Abstract] OR "hazards ratio"[Title/Abstract] OR "hazard ratio"[Title/Abstract]) AND ("1970/01/01"[PDat] : "2023/1/30"[PDat]) NOT (animals[MeSH Terms] NOT Humans[MeSH Terms])

Section 1.1.2: Web of Science Search Strings

Head and Neck Cancers

TS=("smokeless tobacco" OR "Tobacco, Smokeless" OR bajjar OR ("betel quid" AND tobacco) OR (“betel quid” NEAR tobacco) OR "chewing tobacco" OR chimó OR snuff OR snuif OR dip OR dohra OR gudakhu OR gul OR gutka OR gutkha OR "hnat hsey" OR iq’mik OR khaini OR kharra OR khiwam OR khimam OR kiwam OR kimam OR "lal dant manjan" OR ("loose leaf" AND (chew OR tobacco)) OR mainpuri OR maras OR mawa OR mshri OR naffa OR nas OR ((nas OR nass) AND tobacco) OR naswar OR nasway OR nasvay OR neffa OR ((pan OR paan) AND tobacco) OR (plug AND tobacco) OR (rapé AND tobacco) OR ((red OR tobacco) AND (toothpowder OR toothpaste)) OR shammah OR snus OR taaba OR tapkeer OR tawa OR tombol OR toombak OR tuibur OR "tobacco water" OR (twist AND tobacco) OR zarda)

AND

TS=("relative risk" OR "attributable risk" OR "odds ratio" OR "cross-product ratio" OR "hazards ratio" OR "hazard ratio")

AND

TS=((("head and neck" OR pharyngeal OR pharynx OR nasopharyngeal OR nasopharynx OR mouth OR oral OR gingival OR lip OR tongue OR palatal OR "oral cavity" OR laryngeal OR larynx OR esophageal OR esophagus OR oesophageal OR oesophagus OR oropharyngeal OR oropharynx OR hypopharyngeal) NEAR/0 (cancer* OR neoplasm*)) OR "cancer$ of the pharynx" OR "cancer$ of the nasopharynx" OR "neoplasm$ of the nasopharynx" OR "cancer$ of mouth" OR "cancer$ of the mouth" OR "cancer$ of the lip" OR "cancer$ of the tongue" OR "cancer$ of the larynx" OR "neoplasm$ of the larynx" OR "cancer$ of the esophagus" OR "neoplasm$ of the esophagus" OR "cancer$ of the oesophagus" OR "neoplasm$ of the oesophagus" OR "cancer$ of the oropharynx" OR "neoplasm$ of the oropharynx" OR "cancer$ of the hypopharynx" OR "neoplasm$ of the hypopharynx" OR "$esophageal squamous cell carcinoma")

AND

DOP=1970-01-01/2023-01-30

Ischemic Heart Disease

TS=("smokeless tobacco" OR "Tobacco, Smokeless" OR bajjar OR ("betel quid" AND tobacco) OR (“betel quid” NEAR tobacco) OR "chewing tobacco" OR chimó OR snuff OR snuif OR dip OR dohra OR gudakhu OR gul OR gutka OR gutkha OR "hnat hsey" OR iq’mik OR khaini OR kharra OR khiwam OR khimam OR kiwam OR kimam OR "lal dant manjan" OR ("loose leaf" AND (chew OR tobacco)) OR mainpuri OR maras OR mawa OR mshri OR naffa OR nas OR ((nas OR nass) AND tobacco) OR naswar OR nasway OR nasvay OR neffa OR ((pan OR paan) AND tobacco) OR (plug AND tobacco) OR (rapé AND tobacco) OR ((red OR tobacco) AND (toothpowder OR toothpaste)) OR shammah OR snus OR taaba OR tapkeer OR tawa OR tombol OR toombak OR tuibur OR "tobacco water" OR (twist AND tobacco) OR zarda)

AND

TS=("relative risk" OR "attributable risk" OR "odds ratio" OR "cross-product ratio" OR "hazards ratio" OR "hazard ratio")

AND

TS=("coronary artery disease" OR "myocardial isch$emia" OR "cardiac isch$emia" OR "silent isch$emia" OR atherosclerosis OR "isch$emic heart disease" OR "coronary heart disease" OR "myocardial infarct*" OR "heart attack" OR "heart infarct*" OR "cardiac infarct*")

AND

DOP=1970-01-01/2023-01-30

Stroke

TS=("smokeless tobacco" OR "Tobacco, Smokeless" OR bajjar OR ("betel quid" AND tobacco) OR (“betel quid” NEAR tobacco) OR "chewing tobacco" OR chimó OR snuff OR snuif OR dip OR dohra OR gudakhu OR gul OR gutka OR gutkha OR "hnat hsey" OR iq’mik OR khaini OR kharra OR khiwam OR khimam OR kiwam OR kimam OR "lal dant manjan" OR ("loose leaf" AND (chew OR tobacco)) OR mainpuri OR maras OR mawa OR mshri OR naffa OR nas OR ((nas OR nass) AND tobacco) OR naswar OR nasway OR nasvay OR neffa OR ((pan OR paan) AND tobacco) OR (plug AND tobacco) OR (rapé AND tobacco) OR ((red OR tobacco) AND (toothpowder OR toothpaste)) OR shammah OR snus OR taaba OR tapkeer OR tawa OR tombol OR toombak OR tuibur OR "tobacco water" OR (twist AND tobacco) OR zarda)

AND

TS=("relative risk" OR "attributable risk" OR "odds ratio" OR "cross-product ratio" OR "hazards ratio" OR "hazard ratio")

AND

TS=("stroke" OR "cerebrovascular accident" OR ((brain OR cerebral OR cortex OR cerebrovascular OR cortical OR hemisphere OR hemispheric) NEAR/0 infarct*) OR ((intracerebral OR subarachnoid OR brain) NEAR/0 hemorrhage))

AND

DOP=1970-01-01/2023-01-30

Section 1.1.3: Global Index Medicus Search Strings

Head and Neck Cancers

(mh:(F02.145.958* OR J01.637.767.844.500* OR VS2.005.002* OR B01.875.800.575.912.250.093.088* OR HP4.018.127.479*) OR tw:("smokeless tobacco" OR "Tobacco, Smokeless" OR bajjar OR ("betel quid" AND tobacco) OR "chewing tobacco" OR chimó OR snuff OR snuif OR dip OR dohra OR gudakhu OR gul OR gutka OR gutkha OR "hnat hsey" OR iq’mik OR khaini OR kharra OR khiwam OR khimam OR kiwam OR kimam OR "lal dant manjan" OR ("loose leaf" AND (chew OR tobacco)) OR mainpuri OR maras OR mawa OR mshri OR naffa OR nas OR ((nas OR nass) AND tobacco) OR naswar OR nasway OR nasvay OR neffa OR((pan OR paan) AND tobacco) OR (plug AND tobacco) OR (rapé AND tobacco) OR ((red OR tobacco) AND (toothpowder OR toothpaste)) OR shammah OR snus OR taaba OR tapkeer OR tawa OR tombol OR toombak OR tuibur OR "tobacco water" OR (twist AND tobacco) OR zarda))

AND

(mh:(E05.318.740.600.800* OR E05.318.740.600.600*) OR tw: ("relative risk" OR "attributable risk" OR "odds ratio" OR "cross-product ratio" OR "hazards ratio" OR "hazard ratio"))

AND

(year_cluster:[1970 TO 2023])

AND

(mh:(C04.588.443*) OR tw:("head and neck cancer" OR "head and neck cancers" OR "head and neck neoplasm" OR "head and neck neoplasms" OR "pharyngeal cancer" OR "pharyngeal cancers" OR "pharyngeal neoplasm" OR "pharyngeal neoplasms" OR "pharynx cancer" OR "pharynx cancers" OR "pharynx neoplasm" OR "pharynx neoplasms" OR "cancer of the pharynx" OR "cancers of the pharynx" OR "nasopharyngeal cancer" OR "nasopharyngeal cancers" OR "nasopharyngeal neoplasms" OR "nasopharyngeal neoplasm" OR "nasopharynx cancer" OR "nasopharynx cancers" OR "nasopharynx neoplasm" OR "nasopharynx neoplasms" OR "cancer of the nasopharynx" OR "cancers of the nasopharynx" OR "neoplasm of the nasopharynx" OR "neoplasms of the nasopharynx" OR "mouth cancer" OR "mouth cancers" OR "mouth neoplasm" OR "mouth neoplasms" OR "cancer of mouth" OR "cancer of the mouth" OR "cancers of the mouth" OR "oral cancer" OR "oral cancers" OR "oral neoplasm" OR "oral neoplasms" OR "gingival neoplasm" OR "gingival neoplasms" OR "gingival cancer" OR "gingival cancers" OR "lip neoplasm" OR "lip neoplasms" OR "lip cancer" OR "lip cancers" OR "cancer of the lip" OR "cancers of the lip" OR "tongue neoplasm" OR "tongue neoplasms" OR "tongue cancer" OR "tongue cancers" OR "cancer of the tongue" OR "cancers of the tongue" OR "palatal neoplasm" OR "palatal neoplasms" OR "palatal cancer" OR "palatal cancers" OR "oral cavity cancer" OR "oral cavity cancers" OR "laryngeal cancer" OR "laryngeal cancers" OR "laryngeal neoplasm" OR "laryngeal neoplasms" OR "larynx cancer" OR "larynx cancers" OR "larynx neoplasm" OR "larynx neoplasms" OR "cancer of the larynx" OR "cancers of the larynx" OR "neoplasm of the larynx" OR "neoplasms of the larynx" OR "esophageal cancer" OR "esophageal cancers" OR "esophageal neoplasm" OR "esophageal neoplasms" OR "esophagus cancer" OR "esophagus cancers" OR "esophagus neoplasm" OR "esophagus neoplasms" OR "esophageal squamous cell carcinoma" OR "cancer of the esophagus" OR "cancers of the esophagus" OR "neoplasm of the esophagus" OR "neoplasms of the esophagus" OR "oesophageal cancer" OR "oesophageal cancers" OR "oesophageal neoplasm" OR "oesophageal neoplasms" OR "oesophagus cancer" OR "oesophagus cancers" OR "oesophagus neoplasm" OR "oesophagus neoplasms" OR "oesophageal squamous cell carcinoma" OR "cancer of the oesophagus" OR "cancers of the oesophagus" OR "neoplasm of the oesophagus" OR "neoplasms of the oesophagus" OR "oropharyngeal cancer" OR "oropharyngeal cancers" OR "oropharynx cancer" OR "oropharynx cancers" OR "oropharyngeal neoplasms" OR "oropharynx neoplasm" OR "oropharynx neoplasms" OR "cancer of the oropharynx" OR "cancers of the oropharynx" OR "neoplasm of the oropharynx" OR "neoplasms of the oropharynx" OR "hypopharyngeal cancer" OR "hypopharyngeal cancers" OR "hypopharyngeal neoplasms" OR "cancer of the hypopharynx" OR "cancers of the hypopharynx" OR "neoplasm of the hypopharynx" OR "neoplasms of the hypopharynx"))

Ischemic Heart Disease

(tw:("smokeless tobacco" OR "Tobacco, Smokeless" OR bajjar OR ("betel quid" AND tobacco) OR "chewing tobacco" OR chimó OR snuff OR snuif OR dip OR dohra OR gudakhu OR gul OR gutka OR gutkha OR "hnat hsey" OR iq'mik OR khaini OR kharra OR khiwam OR khimam OR kiwam OR kimam OR "lal dant manjan" OR ("loose leaf" AND (chew OR tobacco)) OR mainpuri OR maras OR mawa OR mshri OR naffa OR nas OR ((nas OR nass) AND tobacco) OR naswar OR nasway OR nasvay OR neffa OR((pan OR paan) AND tobacco) OR (plug AND tobacco) OR (rapé AND tobacco) OR ((red OR tobacco) AND (toothpowder OR toothpaste)) OR shammah OR snus OR taaba OR tapkeer OR tawa OR tombol OR toombak OR tuibur OR "tobacco water" OR (twist AND tobacco) OR zarda))

AND

(tw: ("relative risk" OR "attributable risk" OR "odds ratio" OR "cross-product ratio" OR "hazards ratio" OR "hazard ratio"))

AND

(year_cluster:[1970 TO 2023])

AND

(mh:(C14.280.647.250.260* OR C14.280.647* OR C14.907.137.126.307*) OR tw:("coronary artery disease" OR "myocardial ischemia" OR "myocardial ischaemia" OR "cardiac ischemia" OR "cardiac ischaemia" OR "silent ischemia" OR "silent ischaemia" OR "atherosclerosis" OR "ischaemic heart disease" OR "ischemic heart disease" OR "coronary heart disease" OR "myocardial infarct" OR "myocardial infarcts" OR "myocardial infarction" OR "myocardial infarctions" OR "heart attack" OR "heart infarct" OR "heart infarcts" OR "heart infarction" OR "heart infarctions" OR "cardiac infarct" OR "cardiac infarcts" OR "cardiac infarction"))

Stroke

(tw:("smokeless tobacco" OR "Tobacco, Smokeless" OR bajjar OR ("betel quid" AND tobacco) OR "chewing tobacco" OR chimó OR snuff OR snuif OR dip OR dohra OR gudakhu OR gul OR gutka OR gutkha OR "hnat hsey" OR iq'mik OR khaini OR kharra OR khiwam OR khimam OR kiwam OR kimam OR "lal dant manjan" OR ("loose leaf" AND (chew OR tobacco)) OR mainpuri OR maras OR mawa OR mshri OR naffa OR nas OR ((nas OR nass) AND tobacco) OR naswar OR nasway OR nasvay OR neffa OR((pan OR paan) AND tobacco) OR (plug AND tobacco) OR (rapé AND tobacco) OR ((red OR tobacco) AND (toothpowder OR toothpaste)) OR shammah OR snus OR taaba OR tapkeer OR tawa OR tombol OR toombak OR tuibur OR "tobacco water" OR (twist AND tobacco) OR zarda))

AND

(tw: ("relative risk" OR "attributable risk" OR "odds ratio" OR "cross-product ratio" OR "hazards ratio" OR "hazard ratio"))

AND

(year_cluster:[1970 TO 2023])

AND

(mh:(C10.228.140.300.150.477* OR C10.228.140.300.775*OR C10.228.140.300.535*) OR tw:(stroke OR "cerebrovascular accident" OR "brain infarct" OR "brain infarcts" OR "brain infarction" OR "brain infarctions" OR "cerebral infarct" OR "cerebral infarcts" OR "cerebral infarction" OR "cerebral infarctions" OR "cortex infarct" OR "cortex infarcts" OR "cortex infarction" OR "cortex infarctions" OR "cerebrovascular infarct" OR "cerebrovascular infarcts" OR "cerebrovascular infarction" OR "cerebrovascular infarctions" OR "cortical infarct" OR "cortical infarcts" OR "cortical infarction" OR "cortical infarctions" OR "hemisphere infarct" OR "hemisphere infarcts" OR "hemisphere infarction" OR "hemisphere infarctions" OR "hemispheric infarct" OR "hemispheric infarcts" OR "hemispheric infarction" OR "hemispheric infarctions" OR "intracerebral hemorrhage" OR "intracerebral haemorrhage" OR "subarachnoid hemorrhage" OR "subarachnoid haemorrhage" OR "brain hemorrhage"))

Section 1.2: Inclusion and Exclusion Criteria

Studies were screened on the basis of pre-determined exclusion and inclusion criteria. These criteria were readily available for all reviewers to consult and were described in detail to the research team prior to screening. The same criteria were used to resolve conflicts by a third screener.

Exclusion criteria:

- Irrelevant study design: Study does not use a cohort or (nested) case-control study design.
- Non-chewing tobacco exposure: Study does not specifically report results on chewing tobacco or a chewing tobacco product.
- Highly specific sub-population: Study focuses on a very specific sub-population (eg. former cancer survivors or diabetes patients) that would more likely than not interfere with the generalizability of the findings.
- Irrelevant focus: Study does not report on the relationship between chewing tobacco and the relevant outcome.
- Irrelevant outcome: Study does not report on the outcome of interest, even if it reports all other relevant information.
- GBD study: Study reports on Global Burden of Disease results.
- Incorrect exposure categories: Study does report on chewing tobacco, but it does not report binary effect sizes or contains information to manually calculate binary effect sizes.

Inclusion criteria:

- Study design: Study uses a cohort or (nested) case-control study design.
- Risk and focus: Study reports an effect size for chewing tobacco. Studies that use the term smokeless tobacco in locations where chewing tobacco is the overwhelmingly dominant form of smokeless tobacco and no explicit non-chewing form is included in the case definition may be considered.
- Outcome: Study has results for the outcome of interest.

Section 1.3: Outcome Definitions

The outcomes examined in this review were defined in accordance with the health outcome definitions used in the Global Burden of Disease study[1].

Table S1: Definitions of seven included outcomes

Cause Grouping	Cause Name	Definition
Head and neck cancers	Esophageal cancer	Malignant neoplasm of the esophagus
	Laryngeal cancer	Malignant neoplasm of the larynx
	Lip and oral cavity cancer	Malignant neoplasm of the lips and oral cavity
	Nasopharyngeal cancer	Malignant neoplasm of the nasopharynx
	Other pharynx cancer	Malignant neoplasm of the oropharynx, hypopharynx, and other pharynx
Cardiovascular outcomes	Ischemic heart disease	Also referred to as acute myocardial infarction (MI): definite and possible MI according to the third universal definition of myocardial infarction; includes

		recurrent cases and cases who died before reaching medical care
	Stroke	Rapidly developing clinical signs of usually focal disturbance of cerebral function lasting more than 24 h or leading to death

Section 1.4: Exposure Definition

The exposure examined in this review was defined according to the chewing tobacco definition used in the Global Burden of Disease study[2]. We consider chewing tobacco as the current daily or occasional use of chewing tobacco, including local products such as betel quid *with* tobacco. It is a dichotomous risk factor in which the use of chewing tobacco products is compared to non-users (reference).

Table S2: Local smokeless tobacco products categorized as chewing tobacco

**Product**	**Description**	**Mode of use**
Guthka or gutka	Areca nut, slaked lime, catechu and sun-dried, roasted, finely chopped tobacco with flavorings and sweeteners.	Held in mouth and chewed or sucked.
Khat, chat, qaad, qat	Stimulant drug made from the leaves and twigs, evergreen shrub. Active ingredients are cathine and cathinone. We are only interested in Khat *with* tobacco.	Typically chewed — retained in the cheek and chewed.
Man pori, mainpuri, kapoori	Mixture of finely cut betel nut and small pieces of tobacco leaves treated in slaked lime and flavoring agents such as powdered cloves, cardamom, Kewara and sandalwood powder. Catechu is sometimes used.	Chewed and smoked
Mawa	Thin areca nut shavings with slaked lime and tobacco flakes.	Chewed
Paan, pan, betel quid, pan masala or betel nut	Generally contains betel leaf, slaked lime and areca nut, sometimes spices such as clove, saffron, aniseed, turmeric, mustard, or other sweeteners are added. Tobacco is optional. We are only interested in pan or betel *with* tobacco.	Placed in the mouth and chewed.
Sada Pata or chadha	Air cured tobacco. Sometimes used in betel quid.	Chewed or held in mouth.
Zarda	Flaked chewing tobacco flavored with spices, herbs, etc. Often used in betel quid or mixed with with or areca nut.	Chewed

Section 1.5: Data Extraction

Data was extracted using a modified Covidence 2.0 extraction template. The extracted variables are described in the template below.

Table S3: Data Extraction Template

Extraction field name	Field type	Description	Options (if applicable)
NID	Free text	Unique identifier for the data created for the Global Health Data Exchange (GHDx)	
PMID (if available)	Free text	Unique identifier for the data used to link the study to PubMed or other database	
Title	Free text	Title of paper that data are extracted from	
File path	Free text	Full file path for the article where it is stored internally	
Note	Free text	Notes related to extraction, including assumptions, data adjustment, concerns about the source, and anything else that may be useful for the modeler	
Risk	Single choice	What is the risk factor of interest?	Chewing tobacco
Risk mapping	Single choice	What is the relationship between study definition of the risk and the GBD definition of the risk?	Exact; Component; Aggregate; Different form; Unspecified
Study-level risk definition	Free text	How does the study define the relevant risk factor?	
Exposure assessment level	Single choice	Level at which the exposure was assessed. It should always be measured at the individual level for smoking and chewing tobacco	At the individual; Other [fill in]
Exposure assessment period	Single choice	What was the frequency of exposure assessment?	Only at baseline; Multiple times at follow-up; Continuous
Exposure assessment period value	Free text	If the exposure assessment period is multiple times, specify the number of time the exposure was assessed, excluding baseline.	
Exposure recall period	Single choice	Describe the unit of exposure recall used in data collection, primarily for self-reported data.	Lifetime; Point; Period: Days; Period: Weeks; Period: Years; Other [fill in]
Exposure recall period value	Free text	If exposure recall was over a period of time (instead of Lifetime or Point), how long is the period?	

Risk assessment method	Multi-select	Please specify the method of exposure assessment.	Self-report (human/environment); Biomarker (human); Physical measurement (human); Direct observation (human/environment); Disease registry (human); Physical measurement/monitoring (environment)
Risk data source type	Single choice	Identify the underlying mode of data collection for the risk factor.	Case notification – general; Registry – cancer; Registry – other/unknown; Facility – inpatient; Facility – outpatient; Facility – discharge; Facility – long-term; Facility – hospice; Facility – ambulatory; Facility – primary; Facility – other/unknown; Vital registration – national; Vital registration – sample; Vital registration – sentinel; Vital registration – other/unknown; Survey – cross-sectional; Survey – cohort; Survey – longitudinal; Survey – other/unknown; Survey – VA; Survey – Sibling history; Surveillance – VA; Surveillance – facility; Surveillance – other/unknown; Unidentifiable
Study-level outcome	Multi-select	Choose the outcomes that best match the outcome definition used for the study as a whole.<br><br>More detail is provided for each review. Eg. for IHD: Ischemic heart disease is synonymous with coronary heart disease (CHD), coronary artery disease (CAD), and atherosclerotic heart disease. The other options are components of the GBD IHD definition. You should choose the outcomes that best match the outcome definition used in the study.<br>Eg. if a study looks as Myocardial Infraction but does not specify if it looks at ST-elevation or non-ST-elevation, you would choose "Myocardial infraction (MI)."	Ischemic heart disease; Acute coronary syndrome (ACS); Myocardial infraction (MI); Unstable angina; ST-elevation MI (STEMI); Non-ST-elevation MI (NSTEMI); Heart failure due to IHD; Ischemic cardiomyopathy; Sudden cardiac death/arrest; Unheralded/unexpected coronary death; Other [fill in]<br><br>Esophageal cancer; Larynx cancer; Lip and oral cavity cancer; Nasopharynx cancer; Other pharynx cancer; Other [fill in]<br><br>Cerebrovascular disease; Stroke (unspecified); Ischemic stroke/cerebral infarction; Intracerebral hemorrhage; Subarachnoid hemorrhage (SAH); Other [fill in]
Study-level outcome definition	Free text	Provide a brief description of the outcome as reported in the study	
ICD codes (if available)	Free text	Provide the ICD codes reported in the study. If not provided, do not fill in. If provided but not ICD-10, mention what ICD code scheme is used by the study.	
Outcome type	Single choice	Please specify if the outcome definition included incidence of or mortality as a disease endpoint.	Incidence; Mortality; Incidence and/or Mortality
Outcome assessment method	Multi-select	Choose at least one, maximum three	Physician diagnosis; Biomarker; Death certificates; Administrative medical records or disease registries; Self-report

Study/cohort name (if available)	Free text	Enter the name of the study or name used to identify the cohort if provided. Does not equate to the title of the article.	
Study funding source	Free text	List the entities involved	
Possible conflicts of interest for study authors	Free text	List any conflicts of interest disclosed by the authors	
Non-university author affiliation	Free text	List any non-university affiliations included in the author list	
Study location(s): Location name	Table; Free text	Specify the location of the study according to the IHME_Locations document. IHME Location ID refers to the ihme_loc_id value, not the numerical location_id.<br>For studies with subnational data from countries where we model at the subnational level, use one row in the location table to note ONLY the subnational location.<br>For studies with subnational data from countries not in that list, list ONLY the national location(s). Then, enter the specific location name in the question asking for the exact geographic scope of the study below.<br>SUBNATIONAL COUNTRIES: Brazil, China, Ethiopia, India, Indonesia, Iran, Italy, Japan, Kenya, Mexico, New Zealand, Nigeria, Norway, Pakistan, Philippines, Poland, Russian Federation, South Africa, Ukraine, United Kingdom, United States	
Study location(s): IHME location ID	Table; Free text		
Geographically representative	Single choice	Were the study participants geographically representative of the IHME location(s)?	Yes; No
Geographic scope	Free text	If not geographically representative; describe exact geographic scope.	
Representative	Single choice	Where the study participants representative of the study's geographic scope?	Yes; No
Population/selection criteria	Free text	If not representative; describe exact population/selection criteria:	
Mortality among those with disease	Single choice	Is the study looking at mortality among people who have already developed the outcome?	Yes; No

Start year	Free text	Year the study began	
End year	Free text	Year the study ended	
Study design	Single choice	What study design is used to derive the effect size of interest?	Prospective cohort; Case-control; Nested case-control; Case-cohort; Other [fill in]
Follow-up duration: Type of follow-up measure	Table; Free text	For cohort studies only. Possible measures include max, min, mean, median (with a preference for mean or median if available) Possible units include years, months, days	
Follow-up duration: Follow-up units	Table; Free text		
Follow-up duration: Duration (value) of follow-up period	Table; Free text		
Percentage loss to follow-up	Free text	Extract the proportion of individuals in the study (particularly for cohort studies) that were lost to follow-up if this value is reported. Should be a range of 0-1.	
Percentage non-responsive	Free text	Extract the proportion of individuals in the study that were non-responsive to recruitment attempts if this value is reported. Should be a range from 0-1.	
Control pool	Multi-select	For case-control studies. What population are the controls selected from? Hospital includes other patients. Visitors include people accompanying the patients.	Community; Hospital; Visitors; Relatives; Other [fill in]
Exposure assessment point	Single choice	Which form of the exposure was included in relative risk estimation analysis?	Baseline; Cumulative average; Cumulative overall; Change in exposure
ES*: Exposed group definition	Free text	Provide a brief description of the exposed group (i.e., the comparison group) as used to estimate the effect size	
ES*: Exposure temporality	Single choice	What is the temporality of chewing tobacco use that is used for the exposed group for this effect size?	Ever; Current; Former; Never; Non-current (former and never)
ES*: Unexposed group definition	Free text	Provide a brief description of the unexposed group (i.e., the reference group) as used to estimate the effect size	
ES*: Temporality of unexposed group	Single choice	What is the temporality of chewing tobacco use that is used for the unexposed group for this effect size?	Ever; Current; Former; Never; Non-current (former and never)
ES*: Outcome	Multi-select	Choose the outcomes that best match the outcome definition used for this effect size.	Ischemic heart disease; Acute coronary syndrome (ACS); Myocardial infraction (MI); Unstable angina; ST-elevation MI (STEMI); Non-ST-elevation MI (NSTEMI); Heart failure due to IHD; Ischemic cardiomyopathy; Sudden cardiac

			death/arrest; Unheralded/unexpected coronary death; Other [fill in]<br><br>Esophageal cancer; Larynx cancer; Lip and oral cavity cancer; Nasopharynx cancer; Other pharynx cancer; Other [fill in]<br><br>Cerebrovascular disease; Stroke (unspecified); Ischemic stroke/cerebral infarction; Intracerebral hemorrhage; Subarachnoid hemorrhage (SAH); Other [fill in]
ES*: Outcome definition	Free text	Provide a brief description of the outcome as reported in the study specific to this effect size.	
ES*: ICD-10 codes	Free text	Provide the ICD codes reported in the study that are associated with the outcome. If not provided, do not fill in. If provided but not ICD-10, mention what ICD code scheme is used by the study.	
ES*: Confounders controlled for	Multi-select	Select all of the confounders that this effect size is adjusted for as described in the study. List any others not included in the options in the "Other" category, separated by ","	Age; Sex; Education; Income; Smoking: Use of other smokeless products; Alcohol use/drinking; Physical activity; Dietary components; BMI; Hypertension; Diabetes; Hypercholesterolemia; Race or ethnicity; Socioeconomic status; Geographic region; Occupation; Religion; Genotype; Urbanicity; Other [fill in]
ES*: Level of adjustment	Single choice	The effect size's degree of adjustment relative to other models reported in the study.	Most adjusted reported in the study; Least adjusted reported in the study; Manually calculated; Other [fill in]
ES*: Subgroup	Free text	If this effect size is for a subgroup of the study sample, what is the subgroup? Eg. "males" or "females"<br>Should also be reflected in demographic metadata where possible.	
ES*: Smoking status	Single choice	What is the smoking status of the analytic sample used to derive this effect status?	Never smokers; Current smokers; Former smokers; Non-smokers; Any smoking status; Unknown
Age of the effect size sample: Age start	Table; Free text	You need to have either both age start and age end or the mean and standard deviation of the ages. If age start is not provided but age end is, assume 18 if population is adults and 15 otherwise. If age end is not provided but age start is, assume 99 as maximum age end.	
Age of the effect size sample: Age end	Table; Free text		
Age of the effect size sample: Age mean	Table; Free text		
Age of the effect size sample: Age SD	Table; Free text		
Percent male	Table; Free text	A value between and include 1 (all male) and 0 (all female).	

Cohort sample sizes: Exposed person-years	Table; Free text	If study is a cohort study, fill this table in as much as possible. At least one out of the "person-years", "events" or "sample size" column groups need to be filled out. Make sure the effect size number aligns with the metadata specified in that effect size section above.	
Cohort sample sizes: Unexposed person-years	Table; Free text		
Cohort sample sizes: Total person-years	Table; Free text		
Cohort sample sizes: Exposed events/cases	Table; Free text		
Cohort sample sizes: Unexposed events/cases	Table; Free text		
Cohort sample sizes: Total events/cases	Table; Free text		
Cohort sample sizes: Exposed sample size	Table; Free text		
Cohort sample sizes: Unexposed sample size	Table; Free text		
Cohort sample sizes: Total sample size	Table; Free text		
Case-control sample sizes: Exposed cases	Table; Free text	If study is a case-control study, fill this table in as much as possible. You should provide enough information that the modeler could fill in the blank columns by adding and subtracting.<br><br>Make sure the effect size number aligns with the metadata specified in that effect size section above.	
Case-control sample sizes: Exposed controls	Table; Free text		
Case-control sample sizes: Unexposed cases	Table; Free text		
Case-control sample sizes: Unexposed controls	Table; Free text		
Case-control sample sizes: Total cases	Table; Free text		
Case-control sample sizes: Total controls	Table; Free text		
Effect sizes: Effect size	Table; Free text	Extract either CI or SD/SE with each effect size<br>Effect size type: OR, RR, HR<br>CI type: 95, 99, or otherwise the % confidence indicated by upper and lower CI<br>Non-CI uncertainty type: SD or SE<br>Effect size unit: linear, logit, log<br>Make sure the effect size number aligns with the metadata specified in that effect size section above.	
Effect sizes: Effect size type	Table; Free text		
Effect sizes: Lower CI	Table; Free text		
Effect sizes: Upper CI	Table; Free text		
Effect sizes: CI type	Table; Free text		
Effect sizes: Non-CI uncertainty value	Table; Free text		
Effect sizes: Non-CI uncertainty type	Table; Free text		
Effect sizes: Effect size unit	Table; Free text		
Location of effect sizes: Page number	Table; Free text	In supplement: Yes or No<br><br>Page number (where you found effect_size) from literature where you found effect size; Use page number(s) of article, not page # of pdf	
Location of effect sizes: Table number	Table; Free text		
Location of effect sizes: In supplement	Table; Free text		

		Make sure the effect size number aligns with the metadata specified in that effect size section above.	

Section 2: Data Inputs

We extracted our data inputs from peer-reviewed studies identified through the systematic reviews. The data from included studies included in our systematic reviews were extracted by one member of the research team using the extraction template described above. At minimum, the most and least adjusted relevant effect size and related uncertainty reported for each study exposure-outcome definition pairing and analytical sample was extracted, along with the metadata for each effect size and the metadata for the study overall. Reports that presented findings from the same cohort or case-control study population were merged and extracted as a single study to identify all relevant metadata and potential effect sizes for that population. For studies that did not report an effect size for a chewing tobacco product or chewing tobacco broadly and the outcome of interest but did report the number of exposed cases, unexposed cases, exposed controls, and unexposed controls, the extractor manually calculated the unadjusted effect size corresponding to the study design and its related uncertainty.

Section 2.1: Study Characteristics

Table S4: Summary of Study Metadata and Characteristics

Author	Year	Health outcome	Location	Study design	Sex	Age range	Exposure assessment	Outcome assessment method	Endpoint	Sample size	Exposed	Follow-up (years)	Cases	Controls	Control pool	Smoking status	Chewing tobacco type
Akhtar	2012	Esophageal cancer	Sindh	Case-control	Both	20-99	Self-report	Biomarker	Morbidity	NA	NA	N/A	68	335	Hospital	Never smokers	Betel Quid with Tobacco
Chitra	2004	Esophageal cancer	Tamil Nadu	Case-control	Both	N/A	Self-report	Administrative medical records or disease registries	Morbidity	NA	NA	N/A	90	90	Hospital	Any smoking status	Exact
Dar	2012	Esophageal cancer	Jammu & Kashmir and Ladakh	Case-cohort	Both	18-99	Biomarker	Biomarker	Incidence	NA	NA	3.3	702	1,663	Hospital	Any smoking status	Gutkha
Das	2017	Esophageal cancer	Assam	Case-control	Both	40-75	Self-report	Physician diagnosis; Biomarker	Morbidity	NA	NA	N/A	100	100	Hospital	Any smoking status	Betel Quid with Tobacco
Das	2017	Esophageal cancer	Assam	Case-control	Both	40-75	Self-report	Physician diagnosis; Biomarker	Morbidity	NA	NA	N/A	NA	NA	Hospital	Any smoking status	Zarda
Das	2017	Esophageal cancer	Assam	Case-control	Both	40-75	Self-report	Physician diagnosis; Biomarker	Morbidity	NA	NA	N/A	NA	NA	Hospital	Any smoking status	Dhapat
Ganesh	2009	Esophageal cancer	Maharashtra	Case-control	Both	30-75	Self-report	Biomarker	Morbidity	NA	NA	N/A	400	1,458	N/A	Any smoking status	Pan with Tobacco
Ganesh	2009	Esophageal cancer	Maharashtra	Case-control	Both	30-75	Self-report	Biomarker	Morbidity	NA	NA	N/A	296	1,164	N/A	Any smoking status	Exact
Ihsan	2010	Esophageal cancer	Assam	Case-control	Both	18-99	Self-report	Biomarker	Morbidity	NA	NA	N/A	142	185	Unknown	Any smoking status	Exact
Jayalekshmi	2021	Esophageal cancer	Kerala	Prospective cohort	Males	30-84	Self-report	Administrative medical records or disease registries	Incidence	60,737	18,564	23	NA	NA	N/A	Any smoking status	Exact
Joshi	2009	Esophageal cancer	Uttarakhand	Case-control	Both	31-99	Self-report	Physician diagnosis	Morbidity	NA	NA	N/A	94	94	Hospital Visitors	Any smoking status	Exact

Author	Year	Health outcome	Location	Study design	Sex	Age range	Exposure assessment	Outcome assessment method	Endpoint	Sample size	Exposed	Follow-up (years)	Cases	Controls	Control pool	Smoking status	Chewing tobacco type
Jussawalla	1971	Esophageal cancer	Maharashtra, Urban	Case-control	Both	18-99	Self-report	Administrative medical records or disease registries	Morbidity	NA	NA	N/A	305	2005	Community	Non-smokers	Pan with Tobacco
Nandakumar	1996	Esophageal cancer	Karnataka	Case-control	Females	18-99	Self-report	Administrative medical records or disease registries	Morbidity	NA	NA	N/A	121	274	Hospital	Any smoking status	Exact
Nandakumar	1996	Esophageal cancer	Karnataka	Case-control	Males	18-99	Self-report	Administrative medical records or disease registries	Morbidity	NA	NA	N/A	151	327	Hospital	Any smoking status	Exact
Nayar	2000	Esophageal cancer	India	Case-control	Both	18-99	Self-report	Physician diagnosis; Biomarker	Morbidity	NA	NA	N/A	150	150	Relatives	Any smoking status	Betel Quid with Tobacco
Notani	1988	Esophageal cancer	Maharashtra	Case-control	Males	15-99	Self-report	Administrative medical records or disease registries	Morbidity	NA	NA	N/A	236	215	Hospital	Non-smokers	Exact
Phukan	2001	Esophageal cancer	Assam	Case-control	Females	18-99	Self-report	Biomarker	Morbidity	NA	NA	N/A	144	287	Caretakers	Any smoking status	Chadha
Phukan	2001	Esophageal cancer	Assam	Case-control	Males	18-99	Self-report	Biomarker	Morbidity	NA	NA	N/A	358	706	Caretakers	Any smoking status	Chadha
Saikia	2015	Esophageal cancer	Assam	Case-control	Both	N/A	Biomarker	Biomarker	Morbidity	NA	NA	N/A	78	50	Hospital	Any smoking status	Exact
Sankaranarayanan	1991	Esophageal cancer	Kerala	Case-control	Both	18-99	Self-report	Physician diagnosis; Biomarker	Morbidity	NA	NA	N/A	NA	NA	Hospital	Any smoking status	Pan with Tobacco
Sharma	2013	Esophageal cancer	Assam	Case-control	Both	15-80	Self-report	Biomarker	Morbidity	NA	NA	N/A	203	286	Community	Any smoking status	Betel Quid with Tobacco
Sharma	2013	Esophageal cancer	Assam	Case-control	Both	18-80	Self-report	Biomarker	Morbidity	NA	NA	N/A	112	150	Community	Any smoking status	Betel Quid with Tobacco
Talukdar	2013	Esophageal cancer	India	Case-control	Both	25-85	Self-report	Biomarker	Morbidity	NA	NA	N/A	112	130	Community	Any smoking status	Exact

Author	Year	Health outcome	Location	Study design	Sex	Age range	Exposure assessment	Outcome assessment method	Endpoint	Sample size	Exposed	Follow-up (years)	Cases	Controls	Control pool	Smoking status	Chewing tobacco type
Wynder	1977	Esophageal cancer	United States	Case-control	Males	20-89	Self-report	Biomarker	Morbidity	NA	NA	N/A	264	2,560	Hospital	Any smoking status	Exact
Znaor	2003	Esophageal cancer	Tamil Nadu	Case-control	Males	25-99	Biomarker	Biomarker	Morbidity	NA	NA	N/A	602	3453	Hospital	Any smoking status	Exact
Gajalakshmi	2012	Esophageal cancer; Larynx cancer; Lip and oral cavity cancer; Nasopharynx cancer; Other pharynx cancer	Tamil Nadu	Case-control	Females	35-69	Self-report	Death certificates; Self-report	Mortality	NA	NA	N/A	90	47,883	Community	Non-smokers	Exact
Gajalakshmi	2012	Esophageal cancer; Larynx cancer; Lip and oral cavity cancer; Nasopharynx cancer; Other pharynx cancer	Tamil Nadu	Case-control	Females	35-69	Self-report	Death certificates; Self-report	Mortality	NA	NA	N/A	203	219,241	Community	Non-smokers	Exact
Gajalakshmi	2012	Esophageal cancer; Larynx cancer; Lip and oral cavity cancer; Nasopharynx cancer; Other pharynx cancer	Tamil Nadu	Case-control	Males	35-69	Self-report	Death certificates; Self-report	Mortality	NA	NA	N/A	29	23,254	Community	Non-smokers	Exact
Gajalakshmi	2012	Esophageal cancer; Larynx cancer; Lip and oral cavity cancer; Nasopharynx cancer; Other pharynx cancer	Tamil Nadu	Case-control	Males	35-69	Self-report	Death certificates; Self-report	Mortality	NA	NA	N/A	134	138,928	Community	Non-smokers	Exact

Author	Year	Health outcome	Location	Study design	Sex	Age range	Exposure assessment	Outcome assessment method	Endpoint	Sample size	Exposed	Follow-up (years)	Cases	Controls	Control pool	Smoking status	Chewing tobacco type
Gupta	2020	Esophageal cancer; Larynx cancer; Lip and oral cavity cancer; Nasopharynx cancer; Other pharynx cancer	Maharashtra	Case-control	Both	15-99	Self-report	Biomarker	Morbidity	NA	NA	N/A	240	132	Hospital	Any smoking status	Exact
Gupta	2007	Ischemic heart disease	Rajasthan	Case-control	Both	23-76	Self-report	Physician diagnosis	N/A	NA	NA	N/A	200	200	Community	Never smokers	Exact
Henley	2005	Ischemic heart disease	United States	Prospective cohort	Males	30-99	Self-report	Death certificates	N/A	71,503	1,841	18	NA	NA	N/A	Never smokers	Exact
Rahman	2008	Ischemic heart disease	Bangladesh	Case-control	Both	20-49	Self-report	Physician diagnosis	N/A	NA	NA	N/A	69	138	Hospital	Any smoking status	Betel Quid with Tobacco
Rahman	2012	Ischemic heart disease	Bangladesh	Case-control	Both	40-75	Self-report	Physician diagnosis	N/A	NA	NA	N/A	191	1,089	Community; Hospital	Never smokers	Jarda
Ram	2012	Ischemic heart disease	Gujarat	Case-control	Both	31-99	Self-report	Physician diagnosis	N/A	NA	NA	N/A	NA	NA	Hospital	Any smoking status	Exact
Timberlake	2017	Ischemic heart disease	United States	Prospective cohort	Both	15-99	Self-report	Death certificates	N/A	5,856	NA	8.8	NA	NA	N/A	Any smoking status	Exact
Rahman	2012	Ischemic heart disease	Bangladesh	Case-control	Both	40-75	Self-report	Physician diagnosis	N/A	NA	NA	N/A	162	1,089	Community; Hospital	Never smokers	Sada-Pata
Gholap	2023	Larynx cancer	India	Case-control	Males	20-69	Self-report	Physician diagnosis; Biomarker	Morbidity	NA	NA	N/A	6	39	Hospital Visitors	Any smoking status	Betel Quid with Tobacco
Gholap	2023	Larynx cancer	India	Case-control	Males	20-69	Self-report	Physician diagnosis; Biomarker	Morbidity	NA	NA	N/A	2	38	Hospital Visitors	Any smoking status	Gutkha
Gholap	2023	Larynx cancer	India	Case-control	Males	20-69	Self-report	Physician diagnosis; Biomarker	Morbidity	NA	NA	N/A	9	44	Hospital Visitors	Any smoking status	Tobacco with Lime
Jayalekshmi	2013	Larynx cancer	Kerala, Rural	Prospective cohort	Males	30-84	Self-report	Death certificates; Administrative medical records or disease registries	Incidence & Mortality	NA	18,568	13.7	NA	NA	N/A	Any smoking status	Exact

Author	Year	Health outcome	Location	Study design	Sex	Age range	Exposure assessment	Outcome assessment method	Endpoint	Sample size	Exposed	Follow-up (years)	Cases	Controls	Control pool	Smoking status	Chewing tobacco type
Jussawalla	1971	Larynx cancer	Maharashtra, Urban	Case-control	Both	18-99	Self-report	Administrative medical records or disease registries	Morbidity	NA	NA	N/A	560	2005	Community	Non-smokers	Pan with Tobacco
Kapil	2005	Larynx cancer	Delhi	Case-control	Both	18-99	Self-report	Physician diagnosis; Biomarker	Morbidity	NA	NA	N/A	305	305	Hospital	Any smoking status	Exact
Kapil	2005	Larynx cancer	Delhi	Case-control	Both	18-99	Self-report	Physician diagnosis; Biomarker	Morbidity	NA	NA	N/A	305	305	Hospital	Any smoking status	Betel Quid with Tobacco
Notani	1988	Larynx cancer	Maharashtra	Case-control	Males	15-99	Self-report	Administrative medical records or disease registries	Morbidity	NA	NA	N/A	80	215	Hospital	Non-smokers	Exact
Rao	1999	Larynx cancer	Maharashtra	Case-control	Males	18-99	Self-report	Physician diagnosis	Morbidity	NA	NA	N/A	427	631	Hospital	Any smoking status	Exact
Sankaranarayanan	1990	Larynx cancer	Kerala	Case-control	Males	18-99	Self-report	Biomarker	Morbidity	NA	NA	N/A	191	546	Hospital	Any smoking status	Pan with Tobacco
Sapkota	2007	Larynx cancer	Gujarat	Case-control	Both	18-99	Self-report	Administrative medical records or disease registries	Morbidity	NA	NA	N/A	NA	NA	Hospital; Visitors	Any smoking status	Exact
Wynder	1977	Larynx cancer	United States	Case-control	Males	20-89	Self-report	Biomarker	Morbidity	NA	NA	N/A	467	2,560	Hospital	Any smoking status	Exact
Wyss	2016	Larynx cancer	Iowa	Case-control	Both	17-94	Self-report	Administrative medical records or disease registries	Morbidity	NA	NA	N/A	1092	2922	Hospital	Ever smokers	Exact
Akhtar	2016	Larynx cancer; Lip and oral cavity cancer; Nasopharynx cancer; Other pharynx cancer	Punjab	Case-control	Both	18-99	Self-report	Physician diagnosis	Morbidity	NA	NA	N/A	300	300	Relatives	Any smoking status	Exact

Author	Year	Health outcome	Location	Study design	Sex	Age range	Exposure assessment	Outcome assessment method	Endpoint	Sample size	Exposed	Follow-up (years)	Cases	Controls	Control pool	Smoking status	Chewing tobacco type
Anuradha	2019	Larynx cancer; Lip and oral cavity cancer; Nasopharynx cancer; Other pharynx cancer	India	Case-control	Both	21-80	Self-report	Biomarker	Morbidity	NA	NA	N/A	105	110	Hospital	Any smoking status	Exact
Ruwali	2011	Larynx cancer; Lip and oral cavity cancer; Nasopharynx cancer; Other pharynx cancer	Uttar Pradesh	Case-control	Males	18-99	Self-report	Biomarker	Morbidity	NA	NA	N/A	500	500	Clinics	Any smoking status	Exact
Sam	2010	Larynx cancer; Lip and oral cavity cancer; Nasopharynx cancer; Other pharynx cancer	Tamil Nadu	Case-control	Both	18-99	Biomarker	Biomarker	Morbidity	NA	NA	N/A	408	220	Community	Any smoking status	Exact
Sam	2007	Larynx cancer; Lip and oral cavity cancer; Nasopharynx cancer; Other pharynx cancer	Tamil Nadu	Case-control	Both	18-99	Biomarker	Biomarker	Morbidity	NA	NA	N/A	219	210	Hospital	Any smoking status	Exact
Singh	2009	Larynx cancer; Lip and oral cavity cancer; Nasopharynx cancer; Other pharynx cancer	Uttar Pradesh	Case-control	Males	15-99	Self-report	Biomarker	Morbidity	NA	NA	N/A	200	200	Hospital	Any smoking status	Exact
Singh	2008	Larynx cancer; Lip and oral cavity cancer; Nasopharynx cancer; Other pharynx cancer	Uttar Pradesh	Case-control	Males	15-99	Self-report	Biomarker	Morbidity	NA	NA	N/A	78	152	Hospital	Non-smokers	Exact

Author	Year	Health outcome	Location	Study design	Sex	Age range	Exposure assessment	Outcome assessment method	Endpoint	Sample size	Exposed	Follow-up (years)	Cases	Controls	Control pool	Smoking status	Chewing tobacco type
Soya	2007	Larynx cancer; Lip and oral cavity cancer; Nasopharynx cancer; Other pharynx cancer	Tamil Nadu	Case-control	Both	15-99	Self-report	Physician diagnosis; Biomarker	Morbidity	NA	NA	N/A	408	220	Hospital	Any smoking status	Exact
Yadav	2008	Larynx cancer; Lip and oral cavity cancer; Nasopharynx cancer; Other pharynx cancer	Uttar Pradesh	Case-control	Males	15-99	Self-report	Biomarker	Morbidity	NA	NA	N/A	157	228	Community	Non-smokers	Exact
Choudhury	2015	Larynx cancer; Lip and oral cavity cancer; Nasopharynx cancer; Other pharynx cancer	Assam	Case-control	Both	18-99	Self-report	Biomarker	Morbidity	NA	NA	N/A	180	240	Community	Any smoking status	Betel Quid with Tobacco
Chang	2020	Larynx cancer; Lip and oral cavity cancer; Other pharynx cancer	Nepal	Case-control	Both	18-99	Self-report	Physician diagnosis	Morbidity	NA	NA	N/A	549	601	N/A	Any smoking status	Zarda
Ruwali	2009	Larynx cancer; Lip and oral cavity cancer; Other pharynx cancer	Uttar Pradesh	Case-control	Males	18-99	Biomarker	Physician diagnosis; Biomarker	Morbidity	NA	NA	N/A	350	350	Clinics	Any smoking status	Exact
Basu	2008	Larynx cancer; Lip and oral cavity cancer;	West Bengal	Case-control	Both	35-68	Biomarker	Biomarker	Morbidity	NA	NA	N/A	110	110	Hospital	Any smoking status	Gutkha
Akram	2013	Lip and oral cavity cancer	Sindh	Case-control	Both	22-80	Self-report	Physician diagnosis; Biomarker	Morbidity	NA	NA	N/A	NA	NA	Hospital Visitors	Any smoking status	Exact
Amtha	2014	Lip and oral cavity cancer	Jakarta	Case-control	Both	20-99	Self-report	Biomarker	Morbidity	NA	NA	N/A	81	162	Hospital	Any smoking status	Betel Quid with Tobacco
Anantharaman	2007	Lip and oral cavity cancer	Maharashtra	Case-control	Both	18-84	Self-report	Biomarker	Morbidity	NA	NA	N/A	458	729	Hospital	Never smokers	Exact

Author	Year	Health outcome	Location	Study design	Sex	Age range	Exposure assessment	Outcome assessment method	Endpoint	Sample size	Exposed	Follow-up (years)	Cases	Controls	Control pool	Smoking status	Chewing tobacco type
Anantharaman	2007	Lip and oral cavity cancer	Maharashtra	Case-control	Both	18-84	Self-report	Biomarker	Morbidity	NA	NA	N/A	458	729	Hospital	Current smokers	Exact
Arain	2015	Lip and oral cavity cancer	Sindh	Case-control	Females	30-60	Self-report	Biomarker	Morbidity	NA	NA	N/A	NA	NA	Relatives	Any smoking status	Gutkha
Arain	2015	Lip and oral cavity cancer	Sindh	Case-control	Females	30-60	Self-report	Biomarker	Morbidity	NA	NA	N/A	NA	NA	Relatives	Any smoking status	Mainpuri
Arain	2015	Lip and oral cavity cancer	Sindh	Case-control	Males	30-60	Self-report	Biomarker	Morbidity	NA	NA	N/A	NA	NA	Relatives	Any smoking status	Gutkha
Arain	2015	Lip and oral cavity cancer	Sindh	Case-control	Males	30-60	Self-report	Biomarker	Morbidity	NA	NA	N/A	NA	NA	Relatives	Any smoking status	Mainpuri
Awan	2016	Lip and oral cavity cancer	Sindh	Case-control	Both	18-99	Self-report	Biomarker	Morbidity	NA	NA	N/A	134	134	Hospital	Any smoking status	Betel Quid with Tobacco
Awan	2016	Lip and oral cavity cancer	Sindh	Case-control	Both	18-99	Self-report	Biomarker	Morbidity	NA	NA	N/A	134	134	Hospital	Any smoking status	Exact
Awan	2016	Lip and oral cavity cancer	Sindh	Case-control	Both	18-99	Self-report	Biomarker	Morbidity	NA	NA	N/A	134	134	Hospital	Any smoking status	Gutkha
Awan	2016	Lip and oral cavity cancer	Sindh	Case-control	Both	18-99	Self-report	Biomarker	Morbidity	NA	NA	N/A	134	134	Hospital	Any smoking status	Mainpuri
Awan	2016	Lip and oral cavity cancer	Sindh	Case-control	Both	18-99	Self-report	Biomarker	Morbidity	NA	NA	N/A	134	134	Hospital	Any smoking status	Supari
Balaram	2002	Lip and oral cavity cancer	India	Case-cohort	Males	20-85	Self-report	Biomarker	Incidence	NA	NA	2.8	266	269	Hospital	Any smoking status	Pan with Tobacco
Balaram	2002	Lip and oral cavity cancer	India	Case-cohort	Females	18-87	Self-report	Biomarker	Incidence	NA	NA	2.8	251	280	Hospital	Any smoking status	Pan with Tobacco
Buch	2002	Lip and oral cavity cancer	Maharashtra	Case-control	Both	15-99	Self-report	Physician diagnosis	Morbidity	NA	NA	N/A	285	426	Community	Any smoking status	Betel Quid with Tobacco
Bundgaard	1995	Lip and oral cavity cancer	Denmark	Case-control	Both	18-99	Self-report	Physician diagnosis; Biomarker	Morbidity	NA	NA	N/A	161	398	Community	Any smoking status	Exact
Dikshit	2000	Lip and oral cavity cancer	Madhya Pradesh	Case-control	Males	18-99	Self-report	Administrative medical records or disease registries	Morbidity	NA	NA	N/A	NA	NA	Community	Any smoking status	Exact

Author	Year	Health outcome	Location	Study design	Sex	Age range	Exposure assessment	Outcome assessment method	Endpoint	Sample size	Exposed	Follow-up (years)	Cases	Controls	Control pool	Smoking status	Chewing tobacco type
Edirisinghe	2022	Lip and oral cavity cancer	Sri Lanka	Case-control	Both	35-85	Self-report	Physician diagnosis; Biomarker	Morbidity	NA	NA	N/A	33	35	Community	Any smoking status	Betel Quid with Tobacco
Gholap	2023	Lip and oral cavity cancer	India	Case-control	Males	20-69	Self-report	Physician diagnosis; Biomarker	Morbidity	NA	NA	N/A	68	39	Hospital Visitors	Any smoking status	Betel Quid with Tobacco
Gholap	2023	Lip and oral cavity cancer	India	Case-control	Males	20-69	Self-report	Physician diagnosis; Biomarker	Morbidity	NA	NA	N/A	121	38	Hospital Visitors	Any smoking status	Gutkha
Gholap	2023	Lip and oral cavity cancer	India	Case-control	Males	20-69	Self-report	Physician diagnosis; Biomarker	Morbidity	NA	NA	N/A	117	44	Hospital Visitors	Any smoking status	Tobacco with Lime
Goud	1990	Lip and oral cavity cancer	Bihar	Case-control	Both	40-59	Self-report	Administrative medical records or disease registries	Morbidity	NA	NA	N/A	NA	NA	Hospital	Any smoking status	Exact
Gupta	2017	Lip and oral cavity cancer	Maharashtra	Case-control	Both	30-80	Self-report	Biomarker	Morbidity	NA	NA	N/A	187	240	Hospital	Any smoking status	Exact
Gupta	2014	Lip and oral cavity cancer	Uttar Pradesh	Case-control	Both	15-65	Self-report	Physician diagnosis	Morbidity	NA	NA	N/A	66	48	Hospital	Any smoking status	Exact
Herrero	2003	Lip and oral cavity cancer	Karnataka	Case-control	Both	18-99	Self-report	Administrative medical records or disease registries	Morbidity	NA	NA	N/A	540	575	Hospital	Any smoking status	Pan with Tobacco
Ihsan	2011	Lip and oral cavity cancer	India	Case-control	Both	18-99	Self-report	Biomarker	Morbidity	NA	NA	N/A	116	278	Relatives	Any smoking status	Exact
Imam	2021	Lip and oral cavity cancer	Punjab	Case-control	Both	18-99	Self-report	Administrative medical records or disease registries	Morbidity	NA	NA	N/A	94	107	Unknown	Never smokers	Gutkha
Jayalekshmi	2009	Lip and oral cavity cancer	Kerala, Rural	Prospective cohort	Females	30-84	Self-report	Administrative medical records or disease registries	Incidence	78,140	NA	15	NA	NA	N/A	Any smoking status	Exact

Author	Year	Health outcome	Location	Study design	Sex	Age range	Exposure assessment	Outcome assessment method	Endpoint	Sample size	Exposed	Follow-up (years)	Cases	Controls	Control pool	Smoking status	Chewing tobacco type
Jayalekshmi	2011	Lip and oral cavity cancer	Kerala, Rural	Prospective cohort	Males	30-84	Self-report	Physician diagnosis; Death certificates	Incidence	66,277	18,692	15	NA	NA	N/A	Any smoking status	Exact
Jussawalla	1971	Lip and oral cavity cancer	Maharashtra, Urban	Case-control	Both	18-99	Self-report	Administrative medical records or disease registries	Morbidity	NA	NA	N/A	1464	2005	Community	Non-smokers	Pan with Tobacco
Kazi	2010	Lip and oral cavity cancer	Sindh	Case-control	Females	35-65	Self-report	Biomarker	Morbidity	NA	NA	N/A	82	88	Unknown	Non-smokers	Exact
Kietthubthew	2001	Lip and oral cavity cancer	Thailand	Case-control	Both	N/A	Self-report	Administrative medical records or disease registries	Morbidity	NA	NA	N/A	NA	NA	Community	Any smoking status	Betel Quid with Tobacco
Krishna	2014	Lip and oral cavity cancer	Uttar Pradesh	Case-control	Both	18-99	Self-report	Biomarker	Morbidity	NA	NA	N/A	190	189	Hospital	Non-smokers	Exact
Lakhanpal	2014	Lip and oral cavity cancer	India	Case-control	Both	18-99	Self-report	Biomarker	Morbidity	NA	NA	N/A	125	207	Relatives	Any smoking status	Exact
Lohe	2010	Lip and oral cavity cancer	Maharashtra	Case-control	Both	18-80	Self-report	Physician diagnosis; Biomarker	Morbidity	NA	NA	N/A	NA	NA	Hospital	Any smoking status	Betel Quid with Tobacco
Lohe	2010	Lip and oral cavity cancer	Maharashtra	Case-control	Both	18-80	Self-report	Physician diagnosis; Biomarker	Morbidity	NA	NA	N/A	NA	NA	Hospital	Any smoking status	Exact
Madani	2010	Lip and oral cavity cancer	Maharashtra	Case-control	Both	18-99	Self-report	Physician diagnosis; Biomarker	Morbidity	NA	NA	N/A	350	350	Relatives; Visitors	Any smoking status	Gutkha
Madani	2010	Lip and oral cavity cancer	Maharashtra	Case-control	Both	18-99	Self-report	Physician diagnosis; Biomarker	Morbidity	NA	NA	N/A	350	350	Relatives; Visitors	Any smoking status	Tobacco Flakes
Mahapatra	2015	Lip and oral cavity cancer	Karnataka	Case-control	Both	18-83	Self-report	Physician diagnosis; Biomarker	Morbidity	NA	NA	N/A	134	268	Hospital	Any smoking status	Gutkha
Mahapatra	2015	Lip and oral cavity cancer	Karnataka	Case-control	Both	18-83	Self-report	Physician diagnosis; Biomarker	Morbidity	NA	NA	N/A	134	268	Hospital	Any smoking status	Exact

Author	Year	Health outcome	Location	Study design	Sex	Age range	Exposure assessment	Outcome assessment method	Endpoint	Sample size	Exposed	Follow-up (years)	Cases	Controls	Control pool	Smoking status	Chewing tobacco type
Mekala	2020	Lip and oral cavity cancer	Telangana	Case-control	Both	18-99	Self-report	Biomarker	Morbidity	NA	NA	N/A	500	500	Hospital	Any smoking status	Exact
Merchant	2000	Lip and oral cavity cancer	Sindh	Case-control	Both	18-80	Self-report	Physician diagnosis; Biomarker	Morbidity	NA	NA	N/A	79	149	Hospital	Any smoking status	Pan with Tobacco
Merchant	2015	Lip and oral cavity cancer	Sindh	Case-control	Both	18-99	Biomarker	Biomarker	Morbidity	NA	NA	N/A	79	143	Hospital	Any smoking status	Pan with Tobacco
Muwonge	2008	Lip and oral cavity cancer	Kerala	Nested case-control	Both	35-99	Self-report	Physician diagnosis; Biomarker	Incidence	NA	NA	8	282	1,410	Community	Any smoking status	Exact
Nandakumar	1990	Lip and oral cavity cancer	Karnataka	Case-control	Both	15-99	Self-report	Biomarker	Morbidity	NA	NA	N/A	348	348	Hospital	Any smoking status	Pan with Tobacco
Ngelangel	2009	Lip and oral cavity cancer	Philippines	Case-control	Both	N/A	Self-report	Administrative medical records or disease registries	Morbidity	NA	NA	N/A	176	317	Hospital	Any smoking status	Exact
Notani	1988	Lip and oral cavity cancer	Maharashtra	Case-control	Males	15-99	Self-report	Administrative medical records or disease registries	Morbidity	NA	NA	N/A	278	215	Hospital	Non-smokers	Exact
Radoi	2013	Lip and oral cavity cancer	France	Case-control	Both	18-75	Self-report	Physician diagnosis; Biomarker	Morbidity	NA	NA	N/A	772	3,555	Community	Any smoking status	Exact
Rao	1994	Lip and oral cavity cancer	Maharashtra	Case-control	Males	18-99	Biomarker	Biomarker; Administrative medical records or disease registries	N/A	NA	NA	N/A	713	635	Hospital	Any smoking status	Exact
Rao	1998	Lip and oral cavity cancer	Maharashtra	Case-control	Males	25-87	Self-report	Physician diagnosis	Morbidity	NA	NA	N/A	617	615	Hospital	Any smoking status	Exact
Ray	2013	Lip and oral cavity cancer	West Bengal	Case-control	Both	10-99	Self-report	Physician diagnosis; Biomarker	Morbidity	NA	NA	N/A	NA	NA	Hospital	Any smoking status	Exact
Sankaranarayanan	1989	Lip and oral cavity cancer	Kerala	Case-control	Both	18-99	Self-report	Physician diagnosis; Biomarker	Morbidity	NA	NA	N/A	NA	NA	Hospital	Any smoking status	Pan with Tobacco

Author	Year	Health outcome	Location	Study design	Sex	Age range	Exposure assessment	Outcome assessment method	Endpoint	Sample size	Exposed	Follow-up (years)	Cases	Controls	Control pool	Smoking status	Chewing tobacco type
Sankaranarayanan	1990	Lip and oral cavity cancer	Kerala	Case-control	Both	15-99	Self-report	Administrative medical records or disease registries	Morbidity	NA	NA	N/A	402	886	Hospital	Any smoking status	Pan with Tobacco
Sankaranarayanan	1989	Lip and oral cavity cancer	Kerala	Case-control	Both	18-99	Self-report	Administrative medical records or disease registries	Morbidity	NA	NA	N/A	NA	NA	Hospital	Any smoking status	Pan with Tobacco
Sharma	2019	Lip and oral cavity cancer	Delhi	Case-control	Both	18-99	Self-report	Biomarker	Morbidity	NA	NA	N/A	100	150	Hospital	Any smoking status	Exact
Sikdar	2004	Lip and oral cavity cancer	West Bengal	Case-control	Both	15-99	Self-report	Physician diagnosis	Morbidity	NA	NA	N/A	112	144	Hospital	Current smokers	Exact
Singh	2014	Lip and oral cavity cancer	Gujarat	Case-control	Both	N/A	Self-report	Physician diagnosis; Biomarker	Morbidity	NA	NA	N/A	200	200	Hospital; Visitor	Never smokers	Exact
Subapriya	2007	Lip and oral cavity cancer	Tamil Nadu	Case-control	Both	30-75	Self-report	Administrative medical records or disease registries	Morbidity	NA	NA	N/A	251	289	Relatives	Any smoking status	Exact
Subapriya	2007	Lip and oral cavity cancer	Tamil Nadu	Case-control	Both	30-75	Self-report	Administrative medical records or disease registries	Morbidity	NA	NA	N/A	202	266	Relatives	Any smoking status	Betel Quid with Tobacco
Wynder	1977	Lip and oral cavity cancer	United States	Case-control	Males	20-89	Self-report	Biomarker	Morbidity	NA	NA	N/A	NA	NA	Hospital	Any smoking status	Exact
Wyss	2016	Lip and oral cavity cancer	Iowa	Case-control	Both	17-84	Self-report	Administrative medical records or disease registries	Morbidity	NA	NA	N/A	1771	4626	Hospital	Ever smokers	Exact
Wyss	2016	Lip and oral cavity cancer	Iowa	Case-control	Both	17-94	Self-report	Administrative medical records or disease registries	Morbidity	NA	NA	N/A	461	3018	Hospital	Never smokers	Exact
Yadav	2010	Lip and oral cavity cancer	Assam	Case-control	Both	15-99	Self-report	Biomarker	Morbidity	NA	NA	N/A	136	270	Caretakers	Any smoking status	Exact

Author	Year	Health outcome	Location	Study design	Sex	Age range	Exposure assessment	Outcome assessment method	Endpoint	Sample size	Exposed	Follow-up (years)	Cases	Controls	Control pool	Smoking status	Chewing tobacco type
Znaor	2003	Lip and oral cavity cancer	Tamil Nadu	Case-control	Males	25-99	Biomarker	Biomarker	Morbidity	NA	NA	N/A	757	374	Hospital	Any smoking status	Exact
Anantharaman	2014	Lip and oral cavity cancer; Other pharynx cancer	India	Case-control	Both	18-99	Self-report	Biomarker	Morbidity	NA	NA	N/A	3453	602	Hospital	Never smokers	Exact
Dholam	2016	Lip and oral cavity cancer; Other pharynx cancer	India	Case-control	Both	18-45	Self-report	Physician diagnosis	Morbidity	NA	NA	N/A	53	70	Hospital	Never smokers	Exact
Jafarey	1977	Lip and oral cavity cancer; Other pharynx cancer	Sindh	Case-control	Both	18-99	Self-report	Physician diagnosis; Biomarker	Morbidity	NA	NA	N/A	NA	NA	Community	Non-smokers	Exact
Jafarey	1977	Lip and oral cavity cancer; Other pharynx cancer	Sindh	Case-control	Both	18-99	Self-report	Physician diagnosis; Biomarker	Morbidity	NA	NA	N/A	NA	NA	Community	Current smokers	Exact
Kabat	1994	Lip and oral cavity cancer; Other pharynx cancer	United States of America	Case-control	Males	18-80	Self-report	Biomarker; Administrative medical records or disease registries	Morbidity	NA	NA	N/A	82	448	Hospital	Never smokers	Exact
KrishnaRao	2015	Lip and oral cavity cancer; Other pharynx cancer	Karnataka	Case-control	Both	18-99	Self-report	Biomarker	Morbidity	NA	NA	N/A	180	272	Patient Visitors	Any smoking status	Betel Quid with Tobacco
Mashberg	1993	Lip and oral cavity cancer; Other pharynx cancer	New Jersey	Case-control	Males	37-80	Self-report	Physician diagnosis; Biomarker	Morbidity	NA	NA	N/A	359	2,280	Hospital	Any smoking status	Exact
Wyss	2016	Lip and oral cavity cancer; Other pharynx cancer; Nasopharynx cancer	Iowa	Case-control	Both	17-94	Self-report	Administrative medical records or disease registries	Morbidity	NA	NA	N/A	NA		Hospital	Ever smokers	Exact

Author	Year	Health outcome	Location	Study design	Sex	Age range	Exposure assessment	Outcome assessment method	Endpoint	Sample size	Exposed	Follow-up (years)	Cases	Controls	Control pool	Smoking status	Chewing tobacco type
Wyss	2016	Lip and oral cavity cancer; Other pharynx cancer; Nasopharynx cancer	Iowa	Case-control	Both	17-94	Self-report	Administrative medical records or disease registries	Morbidity	NA	NA	N/A	NA	NA	Hospital	Never smokers	Exact
Teo	2006	Myocardial infarction (MI)	Global	Case-control	Both	15-99	Self-report	Physician diagnosis; Biomarker	N/A	NA	NA	N/A	NA	NA	Community; Hospital	Any smoking status	Exact
Panwar	2011	Myocardial infarction (MI); Unstable angina; ST-elevation MI (STEMI); Non-ST-elevation MI (NSTEMI)	Rajasthan	Case-control	Both	15-55	Self-report	Physician diagnosis	N/A	NA	NA	N/A	165	199	Hospital	Any smoking status	Exact
Chelleng	2000	Nasopharynx cancer	Nagaland	Case-control	Both	18-99	Self-report	Biomarker	Morbidity	NA	NA	N/A	47	94	Community	Any smoking status	Exact
Jussawalla	1971	Nasopharynx cancer	Maharashtra, Urban	Case-control	Both	18-99	Self-report	Administrative medical records or disease registries	Morbidity	NA	NA	N/A	17	2005	Community	Non-smokers	Pan with Tobacco
Arain	2015	Nasopharynx cancer; Other pharynx cancer	Sindh	Case-control	Females	30-60	Self-report	Biomarker	Morbidity	NA	NA	N/A	43	120	Relatives	Any smoking status	Gutkha
Arain	2015	Nasopharynx cancer; Other pharynx cancer	Sindh	Case-control	Females	30-60	Self-report	Biomarker	Morbidity	NA	NA	N/A	46	98	Relatives	Any smoking status	Mainpuri
Arain	2015	Nasopharynx cancer; Other pharynx cancer	Sindh	Case-control	Males	30-60	Self-report	Biomarker	Morbidity	NA	NA	N/A	49	158	Relatives	Any smoking status	Gutkha
Arain	2015	Nasopharynx cancer; Other pharynx cancer	Sindh	Case-control	Males	30-60	Self-report	Biomarker	Morbidity	NA	NA	N/A	57	209	Relatives	Any smoking status	Mainpuri

Author	Year	Health outcome	Location	Study design	Sex	Age range	Exposure assessment	Outcome assessment method	Endpoint	Sample size	Exposed	Follow-up (years)	Cases	Controls	Control pool	Smoking status	Chewing tobacco type
Notani	1988	Larynx cancer; esophageal Cancer; Lip and oral cavity cancer; Nasopharynx cancer; Other pharynx cancer	Maharashtra	Case-control	Males	15-99	Self-report	Administrative medical records or disease registries	Morbidity	NA	NA	N/A	225	215	Hospital	Non-smokers	Exact
Wyss	2016	Nasopharynx cancer; Other pharynx cancer	Iowa	Case-control	Both	17-94	Self-report	Administrative medical records or disease registries	Morbidity	NA	NA	N/A	NA	NA	Hospital	Ever smokers	Exact
Wyss	2016	Nasopharynx cancer; Other pharynx cancer	Iowa	Case-control	Both	17-94	Self-report	Administrative medical records or disease registries	Morbidity	NA	NA	N/A	NA	NA	Hospital	Never smokers	Exact
Znaor	2003	Nasopharynx cancer; Other pharynx cancer	Tamil Nadu	Case-control	Males	25-99	Biomarker	Biomarker	Morbidity	NA	NA	N/A	NA	NA	Hospital	Any smoking status	Exact
Dikshit	2000	Other pharynx cancer	Madhya Pradesh	Case-control	Males	18-99	Self-report	Administrative medical records or disease registries	Morbidity	NA	NA	N/A	NA	NA	Community	Any smoking status	Exact
Herrero	2003	Other pharynx cancer	Karnataka	Case-control	Both	18-99	Self-report	Administrative medical records or disease registries	Morbidity	NA	NA	N/A	33	575	Hospital	Any smoking status	Pan with Tobacco
Jayalekshmi	2013	Other pharynx cancer	Kerala, Rural	Prospective cohort	Males	30-84	Self-report	Death certificates; Administrative medical records or disease registries	Incidence & Mortality	NA	18,568	13.7	NA	NA	N/A	Any smoking status	Exact

Author	Year	Health outcome	Location	Study design	Sex	Age range	Exposure assessment	Outcome assessment method	Endpoint	Sample size	Exposed	Follow-up (years)	Cases	Controls	Control pool	Smoking status	Chewing tobacco type
Jussawalla	1971	Other pharynx cancer	Maharashtra, Urban	Case-control	Both	18-99	Self-report	Administrative medical records or disease registries	Morbidity	NA	NA	N/A	70	2005	Community	Non-smokers	Pan with Tobacco
Rao	1999	Other pharynx cancer	Maharashtra	Case-control	Males	18-99	Self-report	Physician diagnosis	Morbidity	NA	NA	N/A	593	631	Hospital	Any smoking status	Exact
Rao	1999	Other pharynx cancer	Maharashtra	Case-control	Males	18-99	Self-report	Physician diagnosis	Morbidity	NA	NA	N/A	678	631	Hospital	Any smoking status	Exact
Sapkota	2007	Other pharynx cancer	Gujarat	Case-control	Both	18-99	Self-report	Administrative medical records or disease registries	Morbidity	NA	NA	N/A	NA	NA	Hospital; Visitors	Any smoking status	Exact
Wasnik	1998	Other pharynx cancer	Maharashtra	Case-control	Both	21-99	Biomarker	Biomarker	Morbidity	NA	NA	N/A	123	246	Hospital	Any smoking status	Exact
Wyss	2016	Other pharynx cancer	Iowa	Case-control	Both	17-94	Self-report	Administrative medical records or disease registries	Morbidity	NA	NA	N/A	NA	NA	Hospital	Ever smokers	Exact
Wyss	2016	Other pharynx cancer	Iowa	Case-control	Both	17-94	Self-report	Administrative medical records or disease registries	Morbidity	NA	NA	N/A	NA	NA	Hospital	Never smokers	Exact
Agashe	2013	Stroke (unspecified)	Maharashtra	Case-control	Males	15-99	Self-report	Physician diagnosis	Morbidity	NA	NA	N/A	80	80	Hospital	Any smoking status	Gutkha
Agashe	2013	Stroke (unspecified)	Maharashtra	Case-control	Males	15-99	Self-report	Physician diagnosis	Morbidity	NA	NA	N/A	80	80	Hospital	Any smoking status	Pan with Tobacco
Gajalakshmi	2015	Stroke (unspecified)	Tamil Nadu	Case-control	Both	35-69	Self-report	Death certificates	Mortality	NA	NA	N/A	NA	429,306	Community	Never smokers	Exact
Mateen	2012	Stroke (unspecified)	Bangladesh	Case-control	Both	20-101	Self-report	Administrative medical records or disease registries	Mortality	NA	NA	N/A	1,250	133	Community	Any smoking status	Betel Quid with Tobacco

Section 2.2: Effect Size Data Details

In the cases of head and neck cancer studies where effect sizes were reported and extracted for aggregate outcomes, we matched those effect sizes to the corresponding component outcomes that align with GBD outcome definitions. As an example, an effect size estimated for lip and oral cancer *and* esophageal cancer combined would be associated both with lip and oral cavity cancer and with esophageal cancer in our datasets for each outcome.

From the extracted and cleaned data points, we identified data points that were eligible for inclusion in our models in order to capture the observation(s) from each included study that would best inform our estimates. This process was undertaken because studies often reported several effect sizes estimated from the same populations for the same health outcome but with slightly different parameters. As an example, a study might report on both head and neck cancers broadly and each sub-outcome specifically; several models with different degrees of adjustment for potential confounders; the risk associated with current chewing and with ever chewing; etc… Including all extracted data points in the models would introduce unnecessary noise and over-represent studies that happened to report more observations. In order to address this challenge, we applied a data point selection process in which each study's extracted data points were evaluated using the following stepwise criteria to identify the most adjusted effect sizes with the closest match to our outcome case definition, our exposure definition, and largest sample size for each analytic sample:

<u>Best match outcome definition:</u>

We selected the observations from each study that best matched the GBD outcome definitions. For studies that reported effect sizes for both aggregate outcome definitions and specific outcome definitions, we selected the specific outcome definitions since these are the ones used in the GBD. For studies that reported effect sizes for several distinct cancer sub-types that mapped to the same GBD outcome, both effect sizes were included with no adjustment since one sub-type is not necessarily a better match for the mapped outcome.

<u>Best match exposure definition:</u>

From the observations with the closest outcome definition, we selected the observations from each study that best matched the chewing tobacco exposure definition used in the GBD. For example, if a study reported on several sub-types of chewing tobacco and on chewing tobacco broadly, we would select the observation that corresponded with the broadest chewing tobacco definition, since our exposure definition is not limited to a specific chewing tobacco product. If a study reported an effect size for ever tobacco chewing and for current tobacco chewing, we preferred the observation of the risk associated with current tobacco chewing.

<u>Most adjusted effect size reported:</u>

If multiple observations from the same study met the above criteria, we selected the most adjusted effect size reported. If there were multiple models that adjusted for the same number of potential confounding variables, we prioritized the models that adjusted for smoking.

<u>Combined sub-groups:</u>

Lastly, if multiple observations still met all of the above criteria, these observations were frequently from studies that reported effect sizes for aggregated and disaggregated sub-groups. Examples of these include observations for males and females combined and separate and multiple study sites being reported as distinct effect sizes and a combined effect size. Since our models are not sex-specific or location-specific, we preferred the effect sizes reported for combined sub-groups over those that were disaggregated. These observations were also informed by large sample sizes.

In the cases where a study still had multiple eligible observations selected after this stepwise process, we examined the differences in the observations to determine whether or not the analytic sample from which they were derived overlapped. Most were studies that only reported the best match and most adjusted effect sizes for disaggregated and mutually exclusive sub-groups, which were all included with no further adjustments. For 13 study-outcome combinations, however, we identified the best match and most adjusted effect sizes for different types of chewing tobacco products without a reported broad chewing tobacco effect size. These groups of users are not mutually exclusive (someone using one form of chewing tobacco is not precluded from using another), but no further information was provided at the study-level regarding the degree of potential overlap between groups. As a result, to avoid the overrepresentation of the individuals in these analytic samples, we down-weighted the data points with overlapping exposure groups based on the number of overlapping observations from that study using the following equation:

$$\begin{aligned} &\textit{standard error used for modeling} \\ &\qquad = \sqrt{\textit{number of overlapping observations}} \times \textit{observed standard error} \end{aligned}$$

Table S5: Summary of Data Inputs

Author	Health outcome	Sample sex	Log(effect size)	Standard error of log(effect size)	Flagged bias covariates	Rational for multiple observations
Agashe 2013	Stroke	Male	0.46	0.45	Geographically representative; Representative; Maximally adjusted; Adjusted for smoking, age, and sex; Chewing tobacco product	Different exposure definitions; Adjusted for non-exclusive samples by a factor of 2
Agashe 2013	Stroke	Male	0.54	1.06	Geographically representative; Representative; Maximally adjusted; Adjusted for smoking, age, and sex; Chewing tobacco product	
Akhtar 2012	Esophageal cancer	Both	2.65	0.41	Geographically representative; Representative; Chewing tobacco temporality; Chewing tobacco product	
Akhtar 2016	Larynx cancer	Both	0.58	0.61	Geographically representative; Representative; Chewing tobacco temporality; Aggregate outcome definition	
Akhtar 2016	Lip and oral cavity cancer	Both	0.58	0.61	Geographically representative; Representative; Chewing tobacco temporality; Aggregate outcome definition	
Akhtar 2016	Nasopharynx cancer	Both	0.58	0.61	Geographically representative; Representative; Chewing tobacco temporality; Aggregate outcome definition	
Akhtar 2016	Other pharynx cancer	Both	0.58	0.61	Geographically representative; Representative; Chewing tobacco temporality; Aggregate outcome definition	
Akram 2013	Lip and oral cavity cancer	Both	4.03	0.65	Geographically representative; Representative; Maximally adjusted; Adjusted for smoking, age, and sex	
Amtha 2014	Lip and oral cavity cancer	Both	1.52	0.72	Geographically representative; Maximally adjusted; Adjusted for smoking, age, and sex; Adjusted for age and sex; Chewing tobacco temporality; Chewing tobacco product	

Anantharaman 2007	Lip and oral cavity cancer	Both	-0.72	0.21	Geographically representative; Representative; Maximally adjusted; Chewing tobacco temporality; Sub-population	Mutually exclusive smoking groups used to stratify analytical samples
Anantharaman 2007	Lip and oral cavity cancer	Both	0.56	0.29	Geographically representative; Representative; Maximally adjusted; Chewing tobacco temporality; Sub-population	
Anantharaman 2014	Lip and oral cavity cancer	Both	2.12	0.18	Geographically representative; Representative; Chewing tobacco temporality; Aggregate outcome definition	
Anantharaman 2014	Other pharynx cancer	Both	2.12	0.18	Geographically representative; Representative; Chewing tobacco temporality; Aggregate outcome definition	
Anuradha 2019	Larynx cancer	Both	0.72	0.35	Geographically representative; Representative; Maximally adjusted; Adjusted for smoking, age, and sex; Chewing tobacco temporality; Aggregate outcome definition	
Anuradha 2019	Lip and oral cavity cancer	Both	0.72	0.35	Geographically representative; Representative; Maximally adjusted; Adjusted for smoking, age, and sex; Chewing tobacco temporality; Aggregate outcome definition	
Anuradha 2019	Nasopharynx cancer	Both	0.72	0.35	Geographically representative; Representative; Maximally adjusted; Adjusted for smoking, age, and sex; Chewing tobacco temporality; Aggregate outcome definition	
Anuradha 2019	Other pharynx cancer	Both	0.72	0.35	Geographically representative; Representative; Maximally adjusted; Adjusted for smoking, age, and sex; Chewing tobacco temporality; Aggregate outcome definition	
Arain 2015	Lip and oral cavity cancer	Female	-0.08	0.25	Geographically representative; Representative; Maximally adjusted; Adjusted for smoking, age, and sex; Adjusted for age and sex; Chewing tobacco product; Sub-population	Separate effect sizes reported for each sex and different types of chewing tobacco products; Adjusted for non-exclusive exposures by a factor of 2
Arain 2015	Lip and oral cavity cancer	Female	0.16	0.26	Geographically representative; Representative; Maximally adjusted; Adjusted for smoking, age, and sex; Adjusted for age and sex; Chewing tobacco product; Sub-population	
Arain 2015	Lip and oral cavity cancer	Male	0.29	0.21	Geographically representative; Representative; Maximally adjusted; Adjusted for smoking, age, and sex; Adjusted for age and sex; Chewing tobacco product; Sub-population	
Arain 2015	Lip and oral cavity cancer	Male	-0.13	0.21	Geographically representative; Representative; Maximally adjusted; Adjusted for smoking, age, and sex; Adjusted for age and sex; Chewing tobacco product; Sub-population	
Arain 2015	Nasopharynx cancer	Female	-0.34	0.50	Geographically representative; Representative; Maximally adjusted; Adjusted for smoking, age, and sex; Adjusted for age and sex; Chewing tobacco product; Sub-population; Aggregate outcome definition	Separate effect sizes reported for each sex and different types of chewing tobacco products; Adjusted for non-exclusive exposures by a factor of 2
Arain 2015	Nasopharynx cancer	Female	0.41	0.44	Geographically representative; Representative; Maximally adjusted; Adjusted for smoking, age, and sex; Adjusted for age and sex; Chewing tobacco product; Sub-population; Aggregate outcome definition	
Arain 2015	Nasopharynx cancer	Male	-0.10	0.42	Geographically representative; Representative; Maximally adjusted; Adjusted for smoking, age, and sex; Adjusted for age and sex; Chewing tobacco product; Sub-population; Aggregate outcome definition	
Arain 2015	Nasopharynx cancer	Male	-0.26	0.40	Geographically representative; Representative; Maximally adjusted; Adjusted for smoking, age, and sex; Adjusted for age and sex; Chewing tobacco product; Sub-population; Aggregate outcome definition	

Arain 2015	Other pharynx cancer	Female	-0.34	0.50	Geographically representative; Representative; Maximally adjusted; Adjusted for smoking, age, and sex; Adjusted for age and sex; Chewing tobacco product; Sub-population; Aggregate outcome definition	Separate effect sizes reported for each sex and different types of chewing tobacco products; Adjusted for non-exclusive exposure groupings by a factor of 2
Arain 2015	Other pharynx cancer	Female	0.41	0.44	Geographically representative; Representative; Maximally adjusted; Adjusted for smoking, age, and sex; Adjusted for age and sex; Chewing tobacco product; Sub-population; Aggregate outcome definition	
Arain 2015	Other pharynx cancer	Male	-0.10	0.42	Geographically representative; Representative; Maximally adjusted; Adjusted for smoking, age, and sex; Adjusted for age and sex; Chewing tobacco product; Sub-population; Aggregate outcome definition	
Arain 2015	Other pharynx cancer	Male	-0.26	0.40	Geographically representative; Representative; Maximally adjusted; Adjusted for smoking, age, and sex; Adjusted for age and sex; Chewing tobacco product; Sub-population; Aggregate outcome definition	
Awan 2016	Lip and oral cavity cancer	Both	0.45	0.77	Geographically representative; Representative; Maximally adjusted; Adjusted for smoking, age, and sex; Adjusted for age and sex; Chewing tobacco temporality; Chewing tobacco product	Effect sizes reported for different chewing tobacco sub-types; Adjusted for non-exclusive samples by a factor of 5
Awan 2016	Lip and oral cavity cancer	Both	0.13	1.76	Geographically representative; Representative; Maximally adjusted; Adjusted for smoking, age, and sex; Adjusted for age and sex; Chewing tobacco temporality	
Awan 2016	Lip and oral cavity cancer	Both	1.71	0.77	Geographically representative; Representative; Maximally adjusted; Adjusted for smoking, age, and sex; Adjusted for age and sex; Chewing tobacco temporality; Chewing tobacco product	
Awan 2016	Lip and oral cavity cancer	Both	0.26	1.46	Geographically representative; Representative; Maximally adjusted; Adjusted for smoking, age, and sex; Adjusted for age and sex; Chewing tobacco temporality; Chewing tobacco product	
Awan 2016	Lip and oral cavity cancer	Both	1.32	1.08	Geographically representative; Representative; Maximally adjusted; Adjusted for smoking, age, and sex; Adjusted for age and sex; Chewing tobacco temporality; Chewing tobacco product	
Balaram 2002	Lip and oral cavity cancer	Male	1.81	0.24	Geographically representative; Representative; Chewing tobacco temporality; Chewing tobacco product; Sub-population	Sex-specific effect sizes were reported with no effect size reported for the aggregate “both” sexes
Balaram 2002	Lip and oral cavity cancer	Female	3.83	0.31	Geographically representative; Representative; Maximally adjusted; Adjusted for smoking, age, and sex; Chewing tobacco temporality; Chewing tobacco product; Sub-population	
Basu 2008	Larynx cancer	Both	0.41	0.92	Geographically representative; Representative; Maximally adjusted; Adjusted for smoking, age, and sex; Chewing tobacco product; Aggregate outcome definition	
Basu 2008	Lip and oral cavity cancer	Both	0.41	0.92	Geographically representative; Representative; Maximally adjusted; Adjusted for smoking, age, and sex; Chewing tobacco product; Aggregate outcome definition	
Buch 2002	Lip and oral cavity cancer	Both	0.49	0.15	Geographically representative; Representative; Maximally adjusted; Adjusted for smoking, age, and sex; Adjusted for age and sex; Chewing tobacco product	
Bundgaard 1995	Lip and oral cavity cancer	Both	0.36	0.45	Geographically representative; Maximally adjusted; Adjusted for smoking, age, and sex; Chewing tobacco temporality	

Chang 2020	Larynx cancer	Both	1.11	0.24	Geographically representative; Representative; Chewing tobacco temporality; Chewing tobacco product; Aggregate outcome definition	
Chang 2020	Lip and oral cavity cancer	Both	1.11	0.24	Geographically representative; Representative; Chewing tobacco temporality; Chewing tobacco product; Aggregate outcome definition	
Chang 2020	Other pharynx cancer	Both	1.11	0.24	Geographically representative; Representative; Chewing tobacco temporality; Chewing tobacco product; Aggregate outcome definition	
Chelleng 2000	Nasopharynx cancer	Both	0.34	0.40	Geographically representative; Representative; Maximally adjusted; Adjusted for smoking, age, and sex	
Chitra 2004	Esophageal cancer	Both	0.92	0.38	Geographically representative; Representative; Maximally adjusted; Adjusted for smoking, age, and sex; Chewing tobacco temporality	
Choudhury 2015	Larynx cancer	Both	0.77	0.21	Geographically representative; Representative; Maximally adjusted; Adjusted for smoking, age, and sex; Adjusted for age and sex; Chewing tobacco temporality; Chewing tobacco product; Aggregate outcome definition	
Choudhury 2015	Lip and oral cavity cancer	Both	0.77	0.21	Geographically representative; Representative; Maximally adjusted; Adjusted for smoking, age, and sex; Adjusted for age and sex; Chewing tobacco temporality; Chewing tobacco product; Aggregate outcome definition	
Choudhury 2015	Nasopharynx cancer	Both	0.77	0.21	Geographically representative; Representative; Maximally adjusted; Adjusted for smoking, age, and sex; Adjusted for age and sex; Chewing tobacco temporality; Chewing tobacco product; Aggregate outcome definition	
Choudhury 2015	Other pharynx cancer	Both	0.77	0.21	Geographically representative; Representative; Maximally adjusted; Adjusted for smoking, age, and sex; Adjusted for age and sex; Chewing tobacco temporality; Chewing tobacco product; Aggregate outcome definition	
Dar 2012	Esophageal cancer	Both	1.05	0.61	Geographically representative; Representative; Chewing tobacco temporality; Chewing tobacco product	
Das 2017	Esophageal cancer	Both	1.91	0.70	Geographically representative; Representative; Maximally adjusted; Adjusted for smoking, age, and sex; Chewing tobacco product	Effect sizes reported for different chewing tobacco sub-types; Adjusted for non-exclusive samples by a factor of 3
Das 2017	Esophageal cancer	Both	1.95	0.83	Geographically representative; Representative; Chewing tobacco product	
Das 2017	Esophageal cancer	Both	1.61	0.77	Geographically representative; Representative; Chewing tobacco product	
Dholam 2016	Lip and oral cavity cancer	Both	2.14	0.42	Geographically representative; Representative; Chewing tobacco temporality; Sub-population; Aggregate outcome definition	
Dholam 2016	Other pharynx cancer	Both	2.14	0.42	Geographically representative; Representative; Chewing tobacco temporality; Sub-population; Aggregate outcome definition	
Dikshit 2000	Lip and oral cavity cancer	Male	1.76	0.25	Geographically representative; Maximally adjusted; Chewing tobacco temporality	
Dikshit 2000	Other pharynx cancer	Male	0.18	0.21	Geographically representative; Maximally adjusted; Chewing tobacco temporality	
Edirisinghe 2022	Lip and oral cavity cancer	Both	1.22	0.55	Representative; Maximally adjusted; Adjusted for smoking, age, and sex; Chewing tobacco product	Effect sizes reported for different chewing tobacco sub-types; Adjusted for non-exclusive samples by a factor of 2
Edirisinghe 2022	Lip and oral cavity cancer	Both	1.45	0.47	Representative; Maximally adjusted; Adjusted for smoking, age, and sex; Chewing tobacco product	
Gajalakshmi 2012	Esophageal cancer	Female	1.34	0.26	Geographically representative; Representative; Chewing tobacco temporality; Outcome assessment; Sub-population; Aggregate outcome definition	Sex-specific effect sizes were reported for mutually exclusive populations (eg. urban and rural) with no

Gajalakshmi 2012	Esophageal cancer	Female	0.99	0.16	Geographically representative; Representative; Chewing tobacco temporality; Outcome assessment; Sub-population; Aggregate outcome definition	aggregate effect sizes reported.
Gajalakshmi 2012	Esophageal cancer	Male	0.64	0.40	Geographically representative; Representative; Chewing tobacco temporality; Outcome assessment; Sub-population; Aggregate outcome definition	
Gajalakshmi 2012	Esophageal cancer	Male	0.79	0.24	Geographically representative; Representative; Chewing tobacco temporality; Outcome assessment; Sub-population; Aggregate outcome definition	
Gajalakshmi 2012	Larynx cancer	Female	1.34	0.26	Geographically representative; Representative; Chewing tobacco temporality; Outcome assessment; Sub-population; Aggregate outcome definition	Sex-specific effect sizes were reported for mutually exclusive populations (eg. urban and rural) with no aggregate effect sizes reported.
Gajalakshmi 2012	Larynx cancer	Female	0.99	0.16	Geographically representative; Representative; Chewing tobacco temporality; Outcome assessment; Sub-population; Aggregate outcome definition	
Gajalakshmi 2012	Larynx cancer	Male	0.64	0.40	Geographically representative; Representative; Chewing tobacco temporality; Outcome assessment; Sub-population; Aggregate outcome definition	
Gajalakshmi 2012	Larynx cancer	Male	0.79	0.24	Geographically representative; Representative; Chewing tobacco temporality; Outcome assessment; Sub-population; Aggregate outcome definition	
Gajalakshmi 2012	Lip and oral cavity cancer	Female	1.34	0.26	Geographically representative; Representative; Chewing tobacco temporality; Outcome assessment; Sub-population; Aggregate outcome definition	Sex-specific effect sizes were reported for mutually exclusive populations (eg. urban and rural) with no aggregate effect sizes reported.
Gajalakshmi 2012	Lip and oral cavity cancer	Female	0.99	0.16	Geographically representative; Representative; Chewing tobacco temporality; Outcome assessment; Sub-population; Aggregate outcome definition	
Gajalakshmi 2012	Lip and oral cavity cancer	Male	0.64	0.40	Geographically representative; Representative; Chewing tobacco temporality; Outcome assessment; Sub-population; Aggregate outcome definition	
Gajalakshmi 2012	Lip and oral cavity cancer	Male	0.79	0.24	Geographically representative; Representative; Chewing tobacco temporality; Outcome assessment; Sub-population; Aggregate outcome definition	
Gajalakshmi 2012	Nasopharynx cancer	Female	1.34	0.26	Geographically representative; Representative; Chewing tobacco temporality; Outcome assessment; Sub-population; Aggregate outcome definition	Sex-specific effect sizes were reported for mutually exclusive populations (eg. urban and rural) with no aggregate effect sizes reported.
Gajalakshmi 2012	Nasopharynx cancer	Female	0.99	0.16	Geographically representative; Representative; Chewing tobacco temporality; Outcome assessment; Sub-population; Aggregate outcome definition	
Gajalakshmi 2012	Nasopharynx cancer	Male	0.64	0.40	Geographically representative; Representative; Chewing tobacco temporality; Outcome assessment; Sub-population; Aggregate outcome definition	
Gajalakshmi 2012	Nasopharynx cancer	Male	0.79	0.24	Geographically representative; Representative; Chewing tobacco temporality; Outcome assessment; Sub-population; Aggregate outcome definition	
Gajalakshmi 2012	Other pharynx cancer	Female	1.34	0.26	Geographically representative; Representative; Chewing tobacco temporality; Outcome assessment; Sub-population; Aggregate outcome definition	Sex-specific effect sizes were reported for mutually exclusive populations (eg. urban and rural) with no

Gajalakshmi 2012	Other pharynx cancer	Female	0.99	0.16	Geographically representative; Representative; Chewing tobacco temporality; Outcome assessment; Sub-population; Aggregate outcome definition	aggregate effect sizes reported.
Gajalakshmi 2012	Other pharynx cancer	Male	0.64	0.40	Geographically representative; Representative; Chewing tobacco temporality; Outcome assessment; Sub-population; Aggregate outcome definition	
Gajalakshmi 2012	Other pharynx cancer	Male	0.79	0.24	Geographically representative; Representative; Chewing tobacco temporality; Outcome assessment; Sub-population; Aggregate outcome definition	
Gajalakshmi 2015	Stroke	Both	0.34	0.07	Geographically representative; Representative; Chewing tobacco temporality	
Ganesh 2009	Esophageal cancer	Both	0.26	0.19	Geographically representative; Representative; Maximally adjusted; Adjusted for smoking, age, and sex; Chewing tobacco temporality; Chewing tobacco product	Effect sizes reported for different chewing tobacco definitions; Adjusted for non-exclusive samples by a factor of 2
Ganesh 2009	Esophageal cancer	Both	0.10	0.38	Geographically representative; Representative; Maximally adjusted; Adjusted for smoking, age, and sex; Chewing tobacco temporality	
Gholap 2023	Larynx cancer	Male	1.74	0.64	Representative; Maximally adjusted; Adjusted for smoking, age, and sex; Chewing tobacco temporality; Chewing tobacco product	Effect sizes reported for different, mutually exclusive chewing tobacco definitions.
Gholap 2023	Larynx cancer	Male	0.92	1.06	Representative; Maximally adjusted; Adjusted for smoking, age, and sex; Chewing tobacco temporality; Chewing tobacco product	
Gholap 2023	Larynx cancer	Male	2.45	0.58	Representative; Maximally adjusted; Adjusted for smoking, age, and sex; Chewing tobacco temporality; Chewing tobacco product	
Gholap 2023	Lip and oral cavity cancer	Male	2.69	0.27	Representative; Maximally adjusted; Adjusted for smoking, age, and sex; Chewing tobacco temporality; Chewing tobacco product	Effect sizes reported for different, mutually exclusive chewing tobacco definitions.
Gholap 2023	Lip and oral cavity cancer	Male	3.52	0.27	Representative; Maximally adjusted; Adjusted for smoking, age, and sex; Chewing tobacco temporality; Chewing tobacco product	
Gholap 2023	Lip and oral cavity cancer	Male	3.19	0.25	Representative; Maximally adjusted; Adjusted for smoking, age, and sex; Chewing tobacco temporality; Chewing tobacco product	
Goud 1990	Lip and oral cavity cancer	Both	2.22	0.32	Geographically representative; Representative; Maximally adjusted; Adjusted for smoking, age, and sex	
Gupta 2007	Ischemic heart disease	Both	-0.49	0.58	Geographically representative; Representative; Maximally adjusted; Adjusted for smoking, age, and sex; Adjusted for age and sex; Chewing tobacco temporality	
Gupta 2014	Lip and oral cavity cancer	Both	-1.21	0.42	Geographically representative; Representative; Maximally adjusted; Adjusted for smoking, age, and sex; Adjusted for age and sex; Chewing tobacco temporality	
Gupta 2017	Lip and oral cavity cancer	Both	2.14	0.28	Geographically representative; Chewing tobacco temporality	
Gupta 2020	Esophageal cancer	Both	4.27	0.37	Geographically representative; Representative; Maximally adjusted; Adjusted for smoking, age, and sex; Chewing tobacco temporality; Aggregate outcome definition	
Gupta 2020	Larynx cancer	Both	4.27	0.37	Geographically representative; Representative; Maximally adjusted; Adjusted for smoking, age, and sex; Chewing tobacco temporality; Aggregate outcome definition	
Gupta 2020	Lip and oral cavity cancer	Both	4.27	0.37	Geographically representative; Representative; Maximally adjusted; Adjusted for smoking, age, and sex; Chewing tobacco temporality; Aggregate outcome definition	

Gupta 2020	Nasopharynx cancer	Both	4.27	0.37	Geographically representative; Representative; Maximally adjusted; Adjusted for smoking, age, and sex; Chewing tobacco temporality; Aggregate outcome definition	
Gupta 2020	Other pharynx cancer	Both	4.27	0.37	Geographically representative; Representative; Maximally adjusted; Adjusted for smoking, age, and sex; Chewing tobacco temporality; Aggregate outcome definition	
Henley 2005	Ischemic heart disease	Male	0.22	0.10	Representative	
Herrero 2003	Lip and oral cavity cancer	Both	2.72	0.15	Geographically representative; Representative; Maximally adjusted; Adjusted for smoking, age, and sex; Chewing tobacco temporality; Chewing tobacco product	
Herrero 2003	Other pharynx cancer	Both	0.15	0.47	Geographically representative; Representative; Maximally adjusted; Adjusted for smoking, age, and sex; Chewing tobacco temporality; Chewing tobacco product	
Ihsan 2010	Esophageal cancer	Both	0.62	0.23	Geographically representative; Representative; Maximally adjusted; Adjusted for smoking, age, and sex; Chewing tobacco temporality	
Ihsan 2011	Lip and oral cavity cancer	Both	1.12	0.27	Geographically representative; Representative; Chewing tobacco temporality	
Imam 2021	Lip and oral cavity cancer	Both	3.08	1.04	Geographically representative; Representative; Maximally adjusted; Adjusted for smoking, age, and sex; Adjusted for age and sex; Chewing tobacco temporality; Chewing tobacco product	
Jafarey 1977	Lip and oral cavity cancer	Both	2.46	0.19	Geographically representative; Sub-population; Aggregate outcome definition	Mutually exclusive samples stratified by smoking status
Jafarey 1977	Lip and oral cavity cancer	Both	-0.04	0.14	Geographically representative; Sub-population; Aggregate outcome definition	
Jafarey 1977	Other pharynx cancer	Both	2.46	0.19	Geographically representative; Sub-population; Aggregate outcome definition	Mutually exclusive samples stratified by smoking status
Jafarey 1977	Other pharynx cancer	Both	-0.04	0.14	Geographically representative; Sub-population; Aggregate outcome definition	
Jayalekshmi 2009	Lip and oral cavity cancer	Female	1.70	0.26	Maximally adjusted; Adjusted for smoking, age, and sex	
Jayalekshmi 2011	Lip and oral cavity cancer	Male	0.88	0.17		
Jayalekshmi 2013	Larynx cancer	Male	-0.11	0.23	Geographically representative	
Jayalekshmi 2013	Other pharynx cancer	Male	-0.11	0.31	Geographically representative	
Jayalekshmi 2021	Esophageal cancer	Male	-0.22	0.18	Maximally adjusted; Adjusted for smoking, age, and sex	
Joshi 2009	Esophageal cancer	Both	0.51	0.29	Geographically representative; Representative; Maximally adjusted; Adjusted for smoking, age, and sex	
Jussawalla 1971	Esophageal cancer	Both	0.93	0.17	Geographically representative; Chewing tobacco product	
Jussawalla 1971	Larynx cancer	Both	1.52	0.17	Geographically representative; Chewing tobacco product	
Jussawalla 1971	Lip and oral cavity cancer	Both	1.79	0.16	Geographically representative; Chewing tobacco product	
Jussawalla 1971	Nasopharynx cancer	Both	0.57	0.71	Geographically representative; Chewing tobacco product	
Jussawalla 1971	Other pharynx cancer	Both	1.83	0.40	Geographically representative; Chewing tobacco product	Effect sizes reported for each component of other pharynx cancer (oropharynx cancer and hypopharynx cancer).
Jussawalla 1971	Other pharynx cancer	Both	1.19	0.19	Geographically representative; Chewing tobacco product	
Kabat 1994	Lip and oral cavity cancer	Male	0.81	0.60	Geographically representative; Representative; Aggregate outcome definition	
Kabat 1994	Other pharynx cancer	Male	0.81	0.60	Geographically representative; Representative; Aggregate outcome definition	

Kapil 2005	Larynx cancer	Both	0.09	0.36	Geographically representative; Representative; Maximally adjusted; Adjusted for smoking, age, and sex	Effect sizes reported for different chewing tobacco definitions; Adjusted for non-exclusive samples by a factor of 2
Kapil 2005	Larynx cancer	Both	0.86	0.54	Geographically representative; Representative; Maximally adjusted; Adjusted for smoking, age, and sex; Chewing tobacco product	
Kazi 2010	Lip and oral cavity cancer	Female	0.61	0.23	Geographically representative; Representative; Maximally adjusted; Adjusted for smoking, age, and sex; Adjusted for age and sex; Chewing tobacco temporality	
Kietthubthew 2001	Lip and oral cavity cancer	Both	0.13	0.49	Geographically representative; Representative; Chewing tobacco product	
Krishna 2014	Lip and oral cavity cancer	Both	1.47	0.36	Geographically representative; Representative; Chewing tobacco temporality	
Krishnarao 2015	Lip and oral cavity cancer	Both	1.10	0.12	Geographically representative; Representative; Maximally adjusted; Adjusted for smoking, age, and sex; Adjusted for age and sex; Chewing tobacco temporality; Chewing tobacco product; Aggregate outcome definition	
Krishnarao 2015	Other pharynx cancer	Both	1.10	0.12	Geographically representative; Representative; Maximally adjusted; Adjusted for smoking, age, and sex; Adjusted for age and sex; Chewing tobacco temporality; Chewing tobacco product; Aggregate outcome definition	
Lakhanpal 2014	Lip and oral cavity cancer	Both	0.11	0.31	Geographically representative; Representative; Maximally adjusted; Adjusted for smoking, age, and sex; Adjusted for age and sex; Chewing tobacco temporality	
Lohe 2010	Lip and oral cavity cancer	Both	3.20	2.53	Geographically representative; Representative; Maximally adjusted; Adjusted for smoking, age, and sex; Chewing tobacco product	Effect sizes reported for different chewing tobacco definitions; Adjusted for non-exclusive samples by a factor of 3
Lohe 2010	Lip and oral cavity cancer	Both	2.40	0.99	Geographically representative; Representative; Maximally adjusted; Adjusted for smoking, age, and sex; Chewing tobacco product	
Lohe 2010	Lip and oral cavity cancer	Both	0.71	0.66	Geographically representative; Representative; Maximally adjusted; Adjusted for smoking, age, and sex	
Madani 2010	Lip and oral cavity cancer	Both	2.52	0.97	Geographically representative; Representative; Chewing tobacco temporality; Chewing tobacco product	Effect sizes reported for different chewing tobacco definitions. This study also reported effect sizes derived from different models with the same degree of adjustment (all adjusting for smoking and a varied set of other confounders); Adjusted for non-exclusive samples by a factor of 10
Madani 2010	Lip and oral cavity cancer	Both	1.81	0.72	Geographically representative; Representative; Chewing tobacco temporality; Chewing tobacco product	
Madani 2010	Lip and oral cavity cancer	Both	2.48	0.96	Geographically representative; Representative; Chewing tobacco temporality; Chewing tobacco product	
Madani 2010	Lip and oral cavity cancer	Both	2.07	0.71	Geographically representative; Representative; Chewing tobacco temporality; Chewing tobacco product	
Madani 2010	Lip and oral cavity cancer	Both	2.54	0.97	Geographically representative; Representative; Chewing tobacco temporality; Chewing tobacco product	
Madani 2010	Lip and oral cavity cancer	Both	2.03	0.72	Geographically representative; Representative; Chewing tobacco temporality; Chewing tobacco product	
Madani 2010	Lip and oral cavity cancer	Both	2.49	0.95	Geographically representative; Representative; Chewing tobacco temporality; Chewing tobacco product	
Madani 2010	Lip and oral cavity cancer	Both	2.12	0.71	Geographically representative; Representative; Chewing tobacco temporality; Chewing tobacco product	
Madani 2010	Lip and oral cavity cancer	Both	2.55	0.48	Geographically representative; Representative; Chewing tobacco temporality; Chewing tobacco product	

Madani 2010	Lip and oral cavity cancer	Both	1.89	0.70	Geographically representative; Representative; Chewing tobacco temporality; Chewing tobacco product	
Mahapatra 2015	Lip and oral cavity cancer	Both	1.63	0.59	Geographically representative; Representative; Chewing tobacco temporality; Chewing tobacco product	Effect sizes reported for different chewing tobacco definitions; Adjusted for non-exclusive samples by a factor of 2
Mahapatra 2015	Lip and oral cavity cancer	Both	1.79	0.69	Geographically representative; Representative; Chewing tobacco temporality	
Mashberg 1993	Lip and oral cavity cancer	Male	0.00	0.18	Geographically representative; Representative; Chewing tobacco temporality; Aggregate outcome definition	
Mashberg 1993	Other pharynx cancer	Male	0.00	0.18	Geographically representative; Representative; Chewing tobacco temporality; Aggregate outcome definition	
Mateen 2012	Stroke	Both	0.86	0.25	Geographically representative; Representative; Maximally adjusted; Adjusted for smoking, age, and sex; Chewing tobacco temporality; Chewing tobacco product	
Mekala 2020	Lip and oral cavity cancer	Both	0.67	0.13	Geographically representative; Representative; Maximally adjusted; Adjusted for smoking, age, and sex; Chewing tobacco temporality	
Merchant 2000	Lip and oral cavity cancer	Both	2.13	0.66	Geographically representative; Representative; Chewing tobacco temporality; Chewing tobacco product	
Merchant 2015	Lip and oral cavity cancer	Both	2.75	0.74	Geographically representative; Representative; Chewing tobacco temporality; Chewing tobacco product	
Muwonge 2008	Lip and oral cavity cancer	Both	1.46	0.17	Geographically representative	
Nandakumar 1990	Lip and oral cavity cancer	Both	2.56	0.22	Geographically representative; Representative; Maximally adjusted; Adjusted for smoking, age, and sex; Chewing tobacco temporality; Chewing tobacco product	
Nandakumar 1996	Esophageal cancer	Female	0.79	0.22	Geographically representative; Representative; Maximally adjusted; Adjusted for smoking, age, and sex; Sub-population	Sex-specific effect sizes were reported in the absence of an effect size for both sexes.
Nandakumar 1996	Esophageal cancer	Male	1.06	0.33	Geographically representative; Representative; Sub-population	
Nayar 2000	Esophageal cancer	Both	0.95	0.37	Geographically representative; Representative; Chewing tobacco product	
Ngelangel 2009	Lip and oral cavity cancer	Both	1.64	0.68	Geographically representative; Representative; Maximally adjusted; Adjusted for smoking, age, and sex; Adjusted for age and sex	
Notani 1988	Esophageal cancer	Male	0.41	0.32	Geographically representative; Representative; Maximally adjusted	
Notani 1988	Larynx cancer	Male	0.59	0.55	Geographically representative; Representative; Maximally adjusted	
Notani 1988	Lip and oral cavity cancer	Male	1.36	0.31	Geographically representative; Representative; Maximally adjusted	
Notani 1988	Nasopharynx cancer	Male	0.83	0.33	Geographically representative; Representative; Maximally adjusted; Aggregate outcome definition	
Notani 1988	Other pharynx cancer	Male	0.83	0.33	Geographically representative; Representative; Maximally adjusted; Aggregate outcome definition	
Panwar 2011	Ischemic heart disease	Both	-0.37	0.22	Geographically representative; Representative; Maximally adjusted; Adjusted for smoking, age, and sex; Chewing tobacco temporality	
Phukan 2001	Esophageal cancer	Female	1.22	0.37	Geographically representative; Chewing tobacco temporality; Chewing tobacco product; Sub-population	Sex-specific effect sizes were reported in the absence of an effect size for both sexes.
Phukan 2001	Esophageal cancer	Male	1.59	0.36	Geographically representative; Chewing tobacco temporality; Chewing tobacco product; Sub-population	

Radoi 2013	Lip and oral cavity cancer	Both	1.48	1.01	Geographically representative; Chewing tobacco temporality	
Rahman 2008	Ischemic heart disease	Both	1.34	0.36	Geographically representative; Representative; Maximally adjusted; Adjusted for smoking, age, and sex; Chewing tobacco temporality; Chewing tobacco product	
Rahman 2012	Ischemic heart disease	Both	-0.45	0.34	Geographically representative; Chewing tobacco product	Effect sizes reported for different chewing tobacco definitions; Adjusted for non-exclusive samples by a factor of 2
Rahman 2012	Ischemic heart disease	Both	0.10	0.76	Geographically representative; Chewing tobacco temporality; Chewing tobacco product	
Ram 2012	Ischemic heart disease	Both	1.18	0.36	Geographically representative; Representative; Maximally adjusted; Adjusted for smoking, age, and sex; Chewing tobacco temporality	
Rao 1994	Lip and oral cavity cancer	Male	1.08	0.12	Geographically representative; Representative; Maximally adjusted; Adjusted for smoking, age, and sex; Adjusted for age and sex; Chewing tobacco temporality	
Rao 1998	Lip and oral cavity cancer	Male	0.12	0.13	Geographically representative; Representative; Chewing tobacco temporality	
Rao 1999	Larynx cancer	Male	0.10	0.15	Geographically representative; Representative; Maximally adjusted; Adjusted for smoking, age, and sex; Adjusted for age and sex; Chewing tobacco temporality	
Rao 1999	Other pharynx cancer	Male	0.10	0.10	Geographically representative; Representative; Maximally adjusted; Adjusted for smoking, age, and sex; Adjusted for age and sex; Chewing tobacco temporality	Effect sizes reported for each component of other pharynx cancer (oropharynx cancer and hypopharynx cancer).
Rao 1999	Other pharynx cancer	Male	-0.36	0.23	Geographically representative; Representative; Maximally adjusted; Adjusted for smoking, age, and sex; Adjusted for age and sex; Chewing tobacco temporality	
Ray 2013	Lip and oral cavity cancer	Both	1.89	0.23	Geographically representative; Representative; Maximally adjusted; Adjusted for smoking, age, and sex; Adjusted for age and sex; Chewing tobacco temporality	
Ruwali 2009	Larynx cancer	Male	0.73	0.16	Geographically representative; Representative; Maximally adjusted; Adjusted for smoking, age, and sex; Adjusted for age and sex; Chewing tobacco temporality; Aggregate outcome definition	
Ruwali 2009	Lip and oral cavity cancer	Male	0.73	0.16	Geographically representative; Representative; Maximally adjusted; Adjusted for smoking, age, and sex; Adjusted for age and sex; Chewing tobacco temporality; Aggregate outcome definition	
Ruwali 2009	Other pharynx cancer	Male	0.73	0.16	Geographically representative; Representative; Maximally adjusted; Adjusted for smoking, age, and sex; Adjusted for age and sex; Chewing tobacco temporality; Aggregate outcome definition	
Ruwali 2011	Larynx cancer	Male	0.73	0.13	Geographically representative; Representative; Chewing tobacco temporality; Aggregate outcome definition	
Ruwali 2011	Lip and oral cavity cancer	Male	0.73	0.13	Geographically representative; Representative; Chewing tobacco temporality; Aggregate outcome definition	
Ruwali 2011	Nasopharynx cancer	Male	0.73	0.13	Geographically representative; Representative; Chewing tobacco temporality; Aggregate outcome definition	
Ruwali 2011	Other pharynx cancer	Male	0.73	0.13	Geographically representative; Representative; Chewing tobacco temporality; Aggregate outcome definition	

Saikia 2015	Esophageal cancer	Both	1.08	0.92	Geographically representative; Representative; Maximally adjusted; Adjusted for smoking, age, and sex; Chewing tobacco temporality	
Sam 2007	Larynx cancer	Both	1.83	0.21	Geographically representative; Representative; Maximally adjusted; Adjusted for smoking, age, and sex; Chewing tobacco temporality; Aggregate outcome definition	
Sam 2007	Lip and oral cavity cancer	Both	1.83	0.21	Geographically representative; Representative; Maximally adjusted; Adjusted for smoking, age, and sex; Chewing tobacco temporality; Aggregate outcome definition	
Sam 2007	Nasopharynx cancer	Both	1.83	0.21	Geographically representative; Representative; Maximally adjusted; Adjusted for smoking, age, and sex; Chewing tobacco temporality; Aggregate outcome definition	
Sam 2007	Other pharynx cancer	Both	1.83	0.21	Geographically representative; Representative; Maximally adjusted; Adjusted for smoking, age, and sex; Chewing tobacco temporality; Aggregate outcome definition	
Sam 2010	Larynx cancer	Both	1.73	0.19	Geographically representative; Representative; Maximally adjusted; Chewing tobacco temporality; Aggregate outcome definition	
Sam 2010	Lip and oral cavity cancer	Both	1.73	0.19	Geographically representative; Representative; Maximally adjusted; Chewing tobacco temporality; Aggregate outcome definition	
Sam 2010	Nasopharynx cancer	Both	1.73	0.19	Geographically representative; Representative; Maximally adjusted; Chewing tobacco temporality; Aggregate outcome definition	
Sam 2010	Other pharynx cancer	Both	1.73	0.19	Geographically representative; Representative; Maximally adjusted; Chewing tobacco temporality; Aggregate outcome definition	
Sankaranarayanan 1989	Lip and oral cavity cancer	Both	1.42	0.18	Geographically representative; Representative; Maximally adjusted; Adjusted for smoking, age, and sex; Chewing tobacco temporality; Chewing tobacco product	These effect sizes were reported in two different studies published with the same lead author in the same year. They are reported for two different component cancers of lip and oral cavity cancers.
Sankaranarayanan 1989	Lip and oral cavity cancer	Both	2.23	0.23	Geographically representative; Representative; Maximally adjusted; Adjusted for smoking, age, and sex; Adjusted for age and sex; Chewing tobacco temporality; Chewing tobacco product	
Sankaranarayanan 1990	Esophageal cancer	Male	0.11	0.18	Geographically representative; Representative; Maximally adjusted; Adjusted for smoking, age, and sex; Adjusted for age and sex; Chewing tobacco temporality; Chewing tobacco product	
Sankaranarayanan 1990	Lip and oral cavity cancer	Both	2.21	0.16	Geographically representative; Representative; Maximally adjusted; Adjusted for smoking, age, and sex; Adjusted for age and sex; Chewing tobacco temporality; Chewing tobacco product	
Sankaranarayanan 1991	Esophageal cancer	Both	0.08	0.14	Geographically representative; Representative; Maximally adjusted; Adjusted for smoking, age, and sex; Adjusted for age and sex; Chewing tobacco temporality; Chewing tobacco product	
Sapkota 2007	Larynx cancer	Both	-0.29	0.30	Geographically representative; Representative; Chewing tobacco temporality; Sub-population	
Sapkota 2007	Other pharynx cancer	Both	0.41	0.17	Geographically representative; Representative; Chewing tobacco temporality; Sub-population	
Sharma 2013	Esophageal cancer	Both	1.21	0.24	Geographically representative; Representative; Maximally adjusted; Adjusted for smoking, age, and sex; Adjusted for age and sex; Chewing tobacco product; Sub-population	Effect sizes reported for two distinct geographic regions with no aggregate effect size reported.
Sharma 2013	Esophageal cancer	Both	0.00	0.51	Geographically representative; Representative; Maximally adjusted; Adjusted for smoking, age, and sex; Adjusted for age and sex; Chewing tobacco product; Sub-population	

Sharma 2019	Lip and oral cavity cancer	Both	1.32	0.37	Geographically representative; Representative; Maximally adjusted; Adjusted for smoking, age, and sex; Adjusted for age and sex
Sikdar 2004	Lip and oral cavity cancer	Both	0.97	0.26	Geographically representative; Representative; Maximally adjusted; Adjusted for smoking, age, and sex; Adjusted for age and sex; Chewing tobacco temporality
Singh 2008	Larynx cancer	Male	2.03	0.33	Geographically representative; Representative; Maximally adjusted; Chewing tobacco temporality; Sub-population; Aggregate outcome definition
Singh 2008	Lip and oral cavity cancer	Male	2.03	0.33	Geographically representative; Representative; Maximally adjusted; Chewing tobacco temporality; Sub-population; Aggregate outcome definition
Singh 2008	Nasopharynx cancer	Male	2.03	0.33	Geographically representative; Representative; Maximally adjusted; Chewing tobacco temporality; Sub-population; Aggregate outcome definition
Singh 2008	Other pharynx cancer	Male	2.03	0.33	Geographically representative; Representative; Maximally adjusted; Chewing tobacco temporality; Sub-population; Aggregate outcome definition
Singh 2009	Larynx cancer	Male	0.51	0.22	Geographically representative; Representative; Maximally adjusted; Adjusted for smoking, age, and sex; Adjusted for age and sex; Chewing tobacco temporality; Aggregate outcome definition
Singh 2009	Lip and oral cavity cancer	Male	0.51	0.22	Geographically representative; Representative; Maximally adjusted; Adjusted for smoking, age, and sex; Adjusted for age and sex; Chewing tobacco temporality; Aggregate outcome definition
Singh 2009	Nasopharynx cancer	Male	0.51	0.22	Geographically representative; Representative; Maximally adjusted; Adjusted for smoking, age, and sex; Adjusted for age and sex; Chewing tobacco temporality; Aggregate outcome definition
Singh 2009	Other pharynx cancer	Male	0.51	0.22	Geographically representative; Representative; Maximally adjusted; Adjusted for smoking, age, and sex; Adjusted for age and sex; Chewing tobacco temporality; Aggregate outcome definition
Singh 2014	Lip and oral cavity cancer	Both	1.52	0.32	Geographically representative; Representative; Maximally adjusted; Adjusted for smoking, age, and sex; Adjusted for age and sex; Chewing tobacco temporality; Sub-population
Soya 2007	Larynx cancer	Both	1.70	0.18	Geographically representative; Representative; Maximally adjusted; Adjusted for smoking, age, and sex; Chewing tobacco temporality; Aggregate outcome definition
Soya 2007	Lip and oral cavity cancer	Both	1.70	0.18	Geographically representative; Representative; Maximally adjusted; Adjusted for smoking, age, and sex; Chewing tobacco temporality; Aggregate outcome definition
Soya 2007	Nasopharynx cancer	Both	1.70	0.18	Geographically representative; Representative; Maximally adjusted; Adjusted for smoking, age, and sex; Chewing tobacco temporality; Aggregate outcome definition
Soya 2007	Other pharynx cancer	Both	1.70	0.18	Geographically representative; Representative; Maximally adjusted; Adjusted for smoking, age, and sex; Chewing tobacco temporality; Aggregate outcome definition

Subapriya 2007	Lip and oral cavity cancer	Both	1.06	1.31	Geographically representative; Representative; Maximally adjusted; Adjusted for smoking, age, and sex; Chewing tobacco temporality; Sub-population	Effect sizes reported for different chewing tobacco definitions; Adjusted for non-exclusive samples by a factor of 2
Subapriya 2007	Lip and oral cavity cancer	Both	1.16	1.31	Geographically representative; Representative; Maximally adjusted; Adjusted for smoking, age, and sex; Chewing tobacco temporality; Chewing tobacco product; Sub-population	
Talukdar 2013	Esophageal cancer	Both	0.97	0.28	Geographically representative; Representative; Chewing tobacco temporality	
Teo 2006	Ischemic heart disease	Both	0.45	0.12	Geographically representative; Representative; Chewing tobacco temporality	
Timberlake 2017	Ischemic heart disease	Both	0.10	0.12	Maximally adjusted; Adjusted for smoking, age, and sex	
Wasnik 1998	Other pharynx cancer	Both	2.08	0.28	Geographically representative; Representative; Chewing tobacco temporality	
Wynder 1977	Esophageal cancer	Male	0.21	0.25	Geographically representative; Representative; Maximally adjusted; Adjusted for smoking, age, and sex; Chewing tobacco temporality	
Wynder 1977	Larynx cancer	Male	0.30	0.17	Geographically representative; Representative; Maximally adjusted; Adjusted for smoking, age, and sex; Chewing tobacco temporality	
Wynder 1977	Lip and oral cavity cancer	Male	0.14	0.15	Geographically representative; Representative; Maximally adjusted; Adjusted for smoking, age, and sex; Chewing tobacco temporality	
Wyss 2016	Larynx cancer	Both	0.10	0.17	Geographically representative; Representative; Chewing tobacco temporality; Sub-population	
Wyss 2016	Lip and oral cavity cancer	Both	-0.06	0.13	Geographically representative; Representative; Chewing tobacco temporality; Aggregate outcome definition	This study reported effect sizes for different mutually exclusive groupings of smoking statuses (eg. Ever smokers and Never smokers) and for different subtypes of lip and oral cavity cancer, including gum cancer.
Wyss 2016	Lip and oral cavity cancer	Both	0.25	0.21	Geographically representative; Representative; Chewing tobacco temporality; Sub-population	
Wyss 2016	Lip and oral cavity cancer	Both	-0.16	0.32	Geographically representative; Representative; Chewing tobacco temporality; Sub-population	
Wyss 2016	Lip and oral cavity cancer	Both	1.12	0.52	Geographically representative; Representative; Chewing tobacco temporality; Sub-population	
Wyss 2016	Lip and oral cavity cancer	Both	-0.14	0.16	Geographically representative; Representative; Chewing tobacco temporality; Sub-population	
Wyss 2016	Lip and oral cavity cancer	Both	0.59	0.28	Geographically representative; Representative; Chewing tobacco temporality; Sub-population	
Wyss 2016	Lip and oral cavity cancer	Both	-0.29	0.54	Geographically representative; Representative; Chewing tobacco temporality; Sub-population	
Wyss 2016	Lip and oral cavity cancer	Both	-0.02	0.28	Geographically representative; Representative; Chewing tobacco temporality; Sub-population; Aggregate outcome definition	
Wyss 2016	Nasopharynx cancer	Both	-0.07	0.13	Geographically representative; Representative; Chewing tobacco temporality; Aggregate outcome definition	This study reported effect sizes for different mutually exclusive groupings of smoking statuses (eg. Ever smokers and Never smokers).
Wyss 2016	Nasopharynx cancer	Both	0.04	0.26	Geographically representative; Representative; Chewing tobacco temporality; Sub-population; Aggregate outcome definition	
Wyss 2016	Other pharynx cancer	Both	-0.06	0.13	Geographically representative; Representative; Chewing tobacco temporality; Aggregate outcome definition	This study reported effect sizes for different mutually exclusive groupings of smoking statuses (eg. Ever smokers and Never smokers) and for different subtypes of other pharynx cancer cancer (oropharynx cancer and hypopharynx cancer)
Wyss 2016	Other pharynx cancer	Both	-0.13	0.29	Geographically representative; Representative; Chewing tobacco temporality; Sub-population	
Wyss 2016	Other pharynx cancer	Both	0.54	0.68	Geographically representative; Representative; Chewing tobacco temporality; Sub-population	
Wyss 2016	Other pharynx cancer	Both	-0.02	0.28	Geographically representative; Representative; Chewing tobacco temporality; Sub-population; Aggregate outcome definition	
Yadav 2008	Larynx cancer	Male	1.85	0.23	Geographically representative; Representative; Chewing tobacco temporality; Sub-population; Aggregate outcome definition	

Yadav 2008	Lip and oral cavity cancer	Male	1.85	0.23	Geographically representative; Representative; Chewing tobacco temporality; Sub-population; Aggregate outcome definition
Yadav 2008	Nasopharynx cancer	Male	1.85	0.23	Geographically representative; Representative; Chewing tobacco temporality; Sub-population; Aggregate outcome definition
Yadav 2008	Other pharynx cancer	Male	1.85	0.23	Geographically representative; Representative; Chewing tobacco temporality; Sub-population; Aggregate outcome definition
Yadav 2010	Lip and oral cavity cancer	Both	0.89	0.26	Geographically representative; Representative; Chewing tobacco temporality
Znaor 2003	Esophageal cancer	Male	0.72	0.12	Geographically representative; Representative; Chewing tobacco temporality
Znaor 2003	Lip and oral cavity cancer	Male	1.62	0.09	Geographically representative; Representative; Chewing tobacco temporality
Znaor 2003	Other pharynx cancer	Male	0.60	0.12	Geographically representative; Representative; Maximally adjusted; Adjusted for smoking, age, and sex; Adjusted for age and sex; Chewing tobacco temporality

Section 3: Supplementary Methods

Section 3.1: Umbrella review

For future GBD rounds and the present study, there was interest in evaluating new risk-outcome pairs that are not currently within the GBD framework for chewing tobacco. To identify new outcomes of public health interest that are likely to have sufficient relevant data to merit a comprehensive systematic review, we conducted an internal umbrella review in March 2021. We used the following search string to identify all meta-analyses and systematic reviews regarding the health risks associated with smokeless tobacco that are indexed in PubMed. It returned 1,102 total hits. One member of the research team screened these results for studies that mentioned being a meta-analysis or systematic review in the title or abstract and categorized these into outcome groupings based on the outcomes of interest described in the publication. A total of 45 meta-analysis/systematic reviews were identified among the 1,102 hits.

We reviewed the full texts of the meta-analysis/systematic reviews that covered outcomes that were discussed by more than one meta-analysis/systematic review according to our categorization. These full texts were evaluated on the basis of available evidence related to chewing tobacco and overall findings. Following this review, we found that there was insufficient data for pancreatic cancer, while meta-analyses regarding smokeless tobacco and cardiovascular diseases, particularly stroke and ischemic heart disease, and smokeless tobacco and head and neck cancers reported mixed findings with sufficient underlying data to indicate that a full systematic review was feasible and merited the application of the Burden of Proof approach.

Section 3.1.1: PubMed Umbrella Review Search String

("smokeless tobacco"[tiab] OR "Tobacco, Smokeless"[Mesh] OR "Dipping Tobacco"[tiab] OR "Oral Tobacco"[tiab] OR bajjar[tiab] OR ("betel quid"[tiab] AND tobacco[tiab]) OR "chewing tobacco"[tiab] OR chimó[tiab] ORsnuff[tiab] OR snuif[tiab] OR dip[tiab] OR dohra[tiab] OR gudakhu[tiab] OR gul[tiab] OR gutka[tiab] OR gutkha[tiab] OR "hnat hsey"[tiab] OR iq'mik[tiab] OR khaini[tiab] OR kharra[tiab] OR khiwam[tiab] OR khimam[tiab] OR kiwam[tiab] ORkimam[tiab] OR "lal dant manjan"[tiab] OR ("loose

leaf"[tiab] AND (chew[tiab] OR tobacco[tiab])) OR mainpuri[tiab] OR maras[tiab] OR mawa[tiab] OR mshri[tiab] OR naffa[tiab] OR nas[Supplementary Concept] OR ((nas[tiab] OR nass[tiab]) AND tobacco[tiab]) OR naswar[tiab] OR nasway[tiab] OR nasvay[tiab] OR neffa[tiab] OR((pan[tiab] OR paan[tiab]) AND tobacco[tiab]) OR (plug[tiab] AND tobacco[tiab]) OR (rapé[tiab] AND tobacco[tiab]) OR ((red[tiab] OR tobacco[tiab]) AND (toothpowder[tiab] OR toothpaste[tiab])) OR shammah[tiab] OR snus[tiab] OR taaba[tiab] OR tapkeer[tiab] OR tawa[tiab] OR tombol[tiab] OR toombak[tiab] OR tuibur[tiab] OR "tobacco water"[tiab] OR (twist[tiab] AND tobacco[tiab]) OR zarda[tiab]) AND ("Risk"[Mesh] OR "relative risk"[tiab] OR "hazard ratio"[tiab] OR"odds ratio"[tiab] OR "rate"[tiab] OR "risk"[tiab]) AND ("Cohort Studies"[Mesh] OR "Prospective Studies"[Mesh] OR "Cohort"[tiab] OR "prospective"[tiab] OR "longitudinal"[tiab] OR "Case-Control Studies"[Mesh] OR "case-control"[tiab] OR "case control"[tiab] OR "case-crossover"[tiab]) AND ("1970/01/01"[PDat] : "2021/12/31"[PDat]) NOT (animals[MeSH] NOT humans[MeSH])

Table S6: Outcome groupings of MA/SRs identified in the umbrella review

Outcome	MA/SR Count
Oral cancer	8
CVD	3
Circulatory diseases	2
Head and neck cancers	2
Pancreatic cancer	2
Diabetes	1
Stroke*	1
Coronary heart disease*	1
Dental caries	1

*Considered in conjunction with the broad cardiovascular disease and circulatory diseases meta-analyses since they would be encompassed in these disease groupings.

Section 3.2: The Scope of the Systematic Literature Reviews

We aimed to capture all existing literature pertaining to the relationship between chewing tobacco use and the seven health outcomes of interest. To do so, we conducted three systematic reviews, each spanning the literature indexed in PubMed, Global Index Medicus, and Web of Science from January 1, 1970 through January 30, 2023. The systematic reviews were managed separately for head and neck cancers, ischemic heart disease, and stroke using Covidence, a systematic review management tool[3]. Each systematic review followed the same protocol:

1. Search string hits for the three databases were uploaded to Covidence for automated de-duplication. Due to limitations in the Covidence platform, any updates needed to be de-duplicated against old hits using Zotero, a citation management tool that was better suited for this purpose.

2. Two independent screeners reviewed the title and abstract of each study report using the pre-determined inclusion and exclusion criteria described above. At the title and abstract screening phase, screeners were not required to specify an exclusion reason for studies that were excluded, but they were directed to be generous in their inclusion to ensure studies with potentially relevant information were not erroneously excluded. Conflicts were resolved by a third reviewer who sought input from other team members if they were uncertain of the appropriate decision. At this stage, special cases (non-English sources and meta-analyses/systematic reviews) were tagged accordingly and included.
3. Two independent screeners reviewed the full text of each study report that were included after the title and abstract phase. To exclude a source, the screeners had to specify an exclusion reason. Conflicts were resolved by a third reviewer who requested feedback from other team members if the appropriate response was uncertain. Any special cases that were not flagged in the title and abstract phase were tagged at this stage. If reviewers identified study populations that were reported by the two or more study reports, these reports were merged to account for a single study and prevent unnecessary duplication.
    a. Meta-analyses/Systematic reviews: These were evaluated based on their title and abstract and full text by two independent screeners and included if they likely captured studies that would meet our inclusion criteria. They were excluded if their focus was entirely extraneous. The citations of included meta-analyses/systematic reviews were reviewed by one screener for potentially relevant underlying studies based on mentions of chewing tobacco, relevant outcomes, and relevant study design. Potentially relevant underlying studies were deduplicated against those mentioned against themselves and against studies that were already included in the primary review. One screener reviewed the title and abstracts of unique new potentially relevant underlying sources, followed by two independent screeners that reviewed the full text of any included sources. Similar to the primary review, a third member of the team resolved any conflicts. Included underlying studies were uploaded to Covidence to be extracted alongside sources from the primary review.
    b. Non-English sources: These sources were evaluated using the same standard protocol as English language sources in the primary review with two independent reviewers for both title/abstract screening and full text screening. When possible, at least one of the two independent reviewers would be an individual sought out for their relevant language skills. If no reviewer or only one reviewer could be found for a given language, team members reviewed the source for inclusion and exclusion using a free online translation tool.
4. Studies that were included after full text review were extracted by a single reviewer using a standardized extraction template.
5. Completed extractions were reviewed and manually vetted for accuracy by a second team member to generate a final dataset.

Table S7: Data inputs for the relative risks of chewing tobacco use

	Total unique sources	Total source-outcome combinations
Dichotomous risk	103	176

Section 3.3: Study Quality and Bias Assessment

We used a covariate selection algorithm that systematically detects eligible covariates that reflected a significant source of bias in the included observations. The method used to evaluate eligible covariates is outlined in more detail in Zheng et al. and in the main manuscript. We created 10 potential bias covariates that were evaluated for their eligibility to be tested in the algorithm and potentially included in the model. The 'gold standard' value was coded as 0, while the alternatives were coded as 1. The covariates were defined as follows:

Level of adjustment for potential confounding variables:

Three bias covariates were generated as a cascading dummy reflecting different levels of adjustment. Gold standard **maximally adjusted** (adj_L0) observations correspond to estimates that were maximally adjusted for age, sex, smoking, and at minimum one other potential confounder. Gold standard **adjusted for smoking, age, and sex** (adj_L1) observations are those that at minimum account for participants' smoking status, age, and sex. Gold standard **adjusted for age and sex** (adj_L2) observations are any that at least control for the age and sex of participants, regardless of their controlling of other potential confounders.

Table S8: Setting up the bias covariates to evaluate the level of adjustment of included data points

Level of adjustment	Definition	Cascading dummy		
		L0	L1	L2
Insufficient	Does not control for age and sex, regardless of whether it controls for other variables	1	1	1
Minimal	Controls only for age and sex	1	1	0
Middle	Controls for age, sex, and smoking	1	0	0
Maximal	Controls for age, sex, smoking, and other confounders	0	0	0

Aggregate outcome definition:

Is the reported effect size calculated using an aggregate outcome definition that includes the outcome of interest? Gold standard observations were defined as effect sizes that were calculated using an outcome definition specific to the outcome of interest.

Chewing tobacco product:

Is the reported effect size calculated using an exposure definition that encompasses chewing tobacco broadly or a specific chewing tobacco product? Gold standard observations were calculated for chewing tobacco broadly.

<u>Chewing tobacco temporality:</u>

Is the reported effect size calculated using current chewing tobacco (product) use or ever chewing tobacco (product) use as the exposed group? Gold standard observations were those that used current exposure as the alternate group and non-current exposure as the reference group because current use more closely aligns with our exposure definition.

<u>Sub-population:</u>

Is the data point calculated from a sub-population of the total study sample? An example is a study that includes smokers and non-smokers but the observation only uses the data from non-smokers. Other examples include sex-specific observations in studies that were not sex-specific or observations for non-alcohol users in studies that included alcohol users. Gold standard observations were estimated from the entire study sample.

<u>Geographical representativeness:</u>

Is the study population geographically representative of the country or sub-national location the study was conducted based on Global Burden of Disease geographies? Studies were considered gold standard if they were geographically representative.

<u>Representativeness:</u>

Is the study sample representative of the location in which the study was conducted? For example, if the study was conducted in a specific neighborhood, the study sample should be representative of the neighborhood. The gold standard is that the study sample is representative of the study's defined geographic scope.

<u>Outcome assessment:</u>

How were the cases for the study ascertained? The gold standard is that outcomes were ascertained through physician diagnosis, disease registry, medical record review, or biomarker examination. The alternate for this bias covariate is that the outcome was self-reported by the study participants.

We did not create a potential bias covariate to evaluate the impact of selection bias because there was a very limited amount of data reporting loss to follow-up or percent for whom data was not ascertained, particularly since the vast majority of our studies use a case-control study design where percent not ascertained is infrequently reported. The high degree of missingness in this variable would create substantial obfuscation of the true patterns of impact selection bias may have had. We also did not create bias covariates for evaluating the form of exposure assessment and reverse causation. All of the studies identified used self-reported exposure data, which is considered gold standard for tobacco use research. For chewing tobacco, unlike for other risk factors, reverse causation is unlikely since individuals are not likely to take up the use of tobacco products when they are already sick with a tobacco-related outcome, so it was deemed to not be relevant for testing.

Table S9: Bias Characteristics for Included Observations

Author	Study design	Health outcome	Geographically representative	Representative	Maximally adjusted	Adjusted for smoking, age, and sex	Adjusted for age and sex	Chewing tobacco temporality	Chewing tobacco product	Outcome assessment	Sub-population	Aggregate outcome definition
Akhtar 2012	Case-control	Esophageal cancer	1	1	0	0	0	1	1	0	0	0
Chitra 2004	Case-control	Esophageal cancer	1	1	1	1	0	1	0	0	0	0
Dar 2012	Case-cohort	Esophageal cancer	1	1	0	0	0	1	1	0	0	0
Das 2017	Case-control	Esophageal cancer	1	1	1	1	0	0	1	0	0	0
Das 2017	Case-control	Esophageal cancer	1	1	0	0	0	0	1	0	0	0
Gajalakshmi 2012	Case-control	Esophageal cancer	1	1	0	0	0	1	0	1	1	1
Ganesh 2009	Case-control	Esophageal cancer	1	1	1	1	0	1	1	0	0	0
Ganesh 2009	Case-control	Esophageal cancer	1	1	1	1	0	1	0	0	0	0
Gupta 2020	Case-control	Esophageal cancer	1	1	1	1	0	1	0	0	0	1
Ihsan 2010	Case-control	Esophageal cancer	1	1	1	1	0	1	0	0	0	0
Jayalekshmi 2021	Prospective cohort	Esophageal cancer	0	0	1	1	0	0	0	0	0	0
Joshi 2009	Case-control	Esophageal cancer	1	1	1	1	0	0	0	0	0	0
Jussawalla 1971	Case-control	Esophageal cancer	1	0	0	0	0	0	1	0	0	0
Nandakumar 1996	Case-control	Esophageal cancer	1	1	1	1	0	0	0	0	1	0
Nandakumar 1996	Case-control	Esophageal cancer	1	1	0	0	0	0	0	0	1	0
Nayar 2000	Case-control	Esophageal cancer	1	1	0	0	0	0	1	0	0	0
Notani 1988	Case-control	Esophageal cancer	1	1	1	0	0	0	0	0	0	0
Phukan 2001	Case-control	Esophageal cancer	1	0	0	0	0	1	1	0	1	0
Saikia 2015	Case-control	Esophageal cancer	1	1	1	1	0	1	0	0	0	0

Author	Study design	Health outcome	Geographically representative	Representative	Maximally adjusted	Adjusted for smoking, age, and sex	Adjusted for age and sex	Chewing tobacco temporality	Chewing tobacco product	Outcome assessment	Sub-population	Aggregate outcome definition
Sankaranarayanan 1990	Case-control	Esophageal cancer	1	1	1	1	1	1	1	0	0	0
Sankaranarayanan 1991	Case-control	Esophageal cancer	1	1	1	1	1	1	1	0	0	0
Sharma 2013	Case-control	Esophageal cancer	1	1	1	1	1	0	1	0	1	0
Talukdar 2013	Case-control	Esophageal cancer	1	1	0	0	0	1	0	0	0	0
Wynder 1977	Case-control	Esophageal cancer	1	1	1	1	0	1	0	0	0	0
Znaor 2003	Case-control	Esophageal cancer	1	1	0	0	0	1	0	0	0	0
Gupta 2007	Case-control	Ischemic heart disease	1	1	1	1	1	1	0	0	0	N/A
Henley 2005	Prospective cohort	Ischemic heart disease	0	1	0	0	0	0	0	0	0	N/A
Panwar 2011	Case-control	Ischemic heart disease	1	1	1	1	0	1	0	0	0	N/A
Rahman 2008	Case-control	Ischemic heart disease	1	1	1	1	0	1	1	0	0	N/A
Rahman 2012	Case-control	Ischemic heart disease	1	0	0	0	0	0	1	0	0	N/A
Rahman 2012	Case-control	Ischemic heart disease	1	0	0	0	0	1	1	0	0	N/A
Ram 2012	Case-control	Ischemic heart disease	1	1	1	1	0	1	0	0	0	N/A
Teo 2006	Case-control	Ischemic heart disease	1	1	0	0	0	1	0	0	0	N/A
Timberlake 2017	Prospective cohort	Ischemic heart disease	0	0	1	1	0	0	0	0	0	N/A
Akhtar 2016	Case-control	Larynx cancer	1	1	0	0	0	1	0	0	0	1
Anuradha 2019	Case-control	Larynx cancer	1	1	1	1	0	1	0	0	0	1

Author	Study design	Health outcome	Geographically representative	Representative	Maximally adjusted	Adjusted for smoking, age, and sex	Adjusted for age and sex	Chewing tobacco temporality	Chewing tobacco product	Outcome assessment	Sub-population	Aggregate outcome definition
Basu 2008	Case-control	Larynx cancer	1	1	1	1	0	0	1	0	0	1
Chang 2020	Case-control	Larynx cancer	1	1	0	0	0	1	1	0	0	1
Choudhury 2015	Case-control	Larynx cancer	1	1	1	1	1	1	1	0	0	1
Gajalakshmi 2012	Case-control	Larynx cancer	1	1	0	0	0	1	0	1	1	1
Gholap 2023	Case-control	Larynx cancer	0	1	1	1	0	1	1	0	0	0
Gupta 2020	Case-control	Larynx cancer	1	1	1	1	0	1	0	0	0	1
Jayalekshmi 2013	Prospective cohort	Larynx cancer	1	0	0	0	0	0	0	0	0	0
Jussawalla 1971	Case-control	Larynx cancer	1	0	0	0	0	0	1	0	0	0
Kapil 2005	Case-control	Larynx cancer	1	1	1	1	0	0	0	0	0	0
Kapil 2005	Case-control	Larynx cancer	1	1	1	1	0	0	1	0	0	0
Notani 1988	Case-control	Larynx cancer	1	1	1	0	0	0	0	0	0	0
Rao 1999	Case-control	Larynx cancer	1	1	1	1	1	1	0	0	0	0
Ruwali 2009	Case-control	Larynx cancer	1	1	1	1	1	1	0	0	0	1
Ruwali 2011	Case-control	Larynx cancer	1	1	0	0	0	1	0	0	0	1
Sam 2007	Case-control	Larynx cancer	1	1	1	1	0	1	0	0	0	1
Sam 2010	Case-control	Larynx cancer	1	1	1	0	0	1	0	0	0	1
Sapkota 2007	Case-control	Larynx cancer	1	1	0	0	0	1	0	0	1	0
Singh 2008	Case-control	Larynx cancer	1	1	1	0	0	1	0	0	1	1
Singh 2009	Case-control	Larynx cancer	1	1	1	1	1	1	0	0	0	1
Soya 2007	Case-control	Larynx cancer	1	1	1	1	0	1	0	0	0	1

Author	Study design	Health outcome	Geographically representative	Representative	Maximally adjusted	Adjusted for smoking, age, and sex	Adjusted for age and sex	Chewing tobacco temporality	Chewing tobacco product	Outcome assessment	Sub-population	Aggregate outcome definition
Wynder 1977	Case-control	Larynx cancer	1	1	1	1	0	1	0	0	0	0
Wyss 2016	Case-control	Larynx cancer	1	1	0	0	0	1	0	0	1	0
Yadav 2008	Case-control	Larynx cancer	1	1	0	0	0	1	0	0	1	1
Akhtar 2016	Case-control	Lip and oral cavity cancer	1	1	0	0	0	1	0	0	0	1
Akram 2013	Case-control	Lip and oral cavity cancer	1	1	1	1	0	0	0	0	0	0
Amtha 2014	Case-control	Lip and oral cavity cancer	1	0	1	1	1	1	1	0	0	0
Ananthar aman 2007	Case-control	Lip and oral cavity cancer	1	1	1	0	0	1	0	0	1	0
Ananthar aman 2014	Case-control	Lip and oral cavity cancer	1	1	0	0	0	1	0	0	0	1
Anuradha 2019	Case-control	Lip and oral cavity cancer	1	1	1	1	0	1	0	0	0	1
Arain 2015	Case-control	Lip and oral cavity cancer	1	1	1	1	1	0	1	0	1	0
Awan 2016	Case-control	Lip and oral cavity cancer	1	1	1	1	1	1	1	0	0	0
Awan 2016	Case-control	Lip and oral cavity cancer	1	1	1	1	1	1	0	0	0	0
Balaram 2002	Case-cohort	Lip and oral cavity cancer	1	1	0	0	0	1	1	0	1	0

Author	Study design	Health outcome	Geographically representative	Representative	Maximally adjusted	Adjusted for smoking, age, and sex	Adjusted for age and sex	Chewing tobacco temporality	Chewing tobacco product	Outcome assessment	Sub-population	Aggregate outcome definition
Balaram 2002	Case-cohort	Lip and oral cavity cancer	1	1	1	1	0	1	1	0	1	0
Basu 2008	Case-control	Lip and oral cavity cancer	1	1	1	1	0	0	1	0	0	1
Buch 2002	Case-control	Lip and oral cavity cancer	1	1	1	1	1	0	1	0	0	0
Bundgaard 1995	Case-control	Lip and oral cavity cancer	1	0	1	1	0	1	0	0	0	0
Chang 2020	Case-control	Lip and oral cavity cancer	1	1	0	0	0	1	1	0	0	1
Choudhury 2015	Case-control	Lip and oral cavity cancer	1	1	1	1	1	1	1	0	0	1
Dholam 2016	Case-control	Lip and oral cavity cancer	1	1	0	0	0	1	0	0	1	1
Dikshit 2000	Case-control	Lip and oral cavity cancer	1	0	1	0	0	1	0	0	0	0
Edirisinghe 2022	Case-control	Lip and oral cavity cancer	0	1	1	1	0	0	1	0	0	0
Gajalakshmi 2012	Case-control	Lip and oral cavity cancer	1	1	0	0	0	1	0	1	1	1
Gholap 2023	Case-control	Lip and oral cavity cancer	0	1	1	1	0	1	1	0	0	0
Goud 1990	Case-control	Lip and oral cavity cancer	1	1	1	1	0	0	0	0	0	0

Author	Study design	Health outcome	Geographically representative	Representative	Maximally adjusted	Adjusted for smoking, age, and sex	Adjusted for age and sex	Chewing tobacco temporality	Chewing tobacco product	Outcome assessment	Sub-population	Aggregate outcome definition
Gupta 2014	Case-control	Lip and oral cavity cancer	1	1	1	1	1	1	0	0	0	0
Gupta 2017	Case-control	Lip and oral cavity cancer	1	0	0	0	0	1	0	0	0	0
Gupta 2020	Case-control	Lip and oral cavity cancer	1	1	1	1	0	1	0	0	0	1
Herrero 2003	Case-control	Lip and oral cavity cancer	1	1	1	1	0	1	1	0	0	0
Ihsan 2011	Case-control	Lip and oral cavity cancer	1	1	0	0	0	1	0	0	0	0
Imam 2021	Case-control	Lip and oral cavity cancer	1	1	1	1	1	1	1	0	0	0
Jafarey 1977	Case-control	Lip and oral cavity cancer	1	0	0	0	0	0	0	0	1	1
Jayalekshmi 2009	Prospective cohort	Lip and oral cavity cancer	0	0	1	1	0	0	0	0	0	0
Jayalekshmi 2011	Prospective cohort	Lip and oral cavity cancer	0	0	0	0	0	0	0	0	0	0
Jussawalla 1971	Case-control	Lip and oral cavity cancer	1	0	0	0	0	0	1	0	0	0
Kabat 1994	Case-control	Lip and oral cavity cancer	1	1	0	0	0	0	0	0	0	1
Kazi 2010	Case-control	Lip and oral cavity cancer	1	1	1	1	1	1	0	0	0	0

Author	Study design	Health outcome	Geographically representative	Representative	Maximally adjusted	Adjusted for smoking, age, and sex	Adjusted for age and sex	Chewing tobacco temporality	Chewing tobacco product	Outcome assessment	Sub-population	Aggregate outcome definition
Kietthubthew 2001	Case-control	Lip and oral cavity cancer	1	1	0	0	0	0	1	0	0	0
Krishna 2014	Case-control	Lip and oral cavity cancer	1	1	0	0	0	1	0	0	0	0
Krishnarao 2015	Case-control	Lip and oral cavity cancer	1	1	1	1	1	1	1	0	0	1
Lakhanpal 2014	Case-control	Lip and oral cavity cancer	1	1	1	1	1	1	0	0	0	0
Lohe 2010	Case-control	Lip and oral cavity cancer	1	1	1	1	0	0	1	0	0	0
Lohe 2010	Case-control	Lip and oral cavity cancer	1	1	1	1	0	0	0	0	0	0
Madani 2010	Case-control	Lip and oral cavity cancer	1	1	0	0	0	1	1	0	0	0
Mahapatra 2015	Case-control	Lip and oral cavity cancer	1	1	0	0	0	1	1	0	0	0
Mahapatra 2015	Case-control	Lip and oral cavity cancer	1	1	0	0	0	1	0	0	0	0
Mashberg 1993	Case-control	Lip and oral cavity cancer	1	1	0	0	0	1	0	0	0	1
Mekala 2020	Case-control	Lip and oral cavity cancer	1	1	1	1	0	1	0	0	0	0
Merchant 2000	Case-control	Lip and oral cavity cancer	1	1	0	0	0	1	1	0	0	0

Author	Study design	Health outcome	Geographically representative	Representative	Maximally adjusted	Adjusted for smoking, age, and sex	Adjusted for age and sex	Chewing tobacco temporality	Chewing tobacco product	Outcome assessment	Sub-population	Aggregate outcome definition
Merchant 2015	Case-control	Lip and oral cavity cancer	1	1	0	0	0	1	1	0	0	0
Muwonge 2008	Nested case-control	Lip and oral cavity cancer	1	0	0	0	0	0	0	0	0	0
Nandakumar 1990	Case-control	Lip and oral cavity cancer	1	1	1	1	0	1	1	0	0	0
Ngelangel 2009	Case-control	Lip and oral cavity cancer	1	1	1	1	1	0	0	0	0	0
Notani 1988	Case-control	Lip and oral cavity cancer	1	1	1	0	0	0	0	0	0	0
Radoi 2013	Case-control	Lip and oral cavity cancer	1	0	0	0	0	1	0	0	0	0
Rao 1994	Case-control	Lip and oral cavity cancer	1	1	1	1	1	1	0	0	0	0
Rao 1998	Case-control	Lip and oral cavity cancer	1	1	0	0	0	1	0	0	0	0
Ray 2013	Case-control	Lip and oral cavity cancer	1	1	1	1	1	1	0	0	0	0
Ruwali 2009	Case-control	Lip and oral cavity cancer	1	1	1	1	1	1	0	0	0	1
Ruwali 2011	Case-control	Lip and oral cavity cancer	1	1	0	0	0	1	0	0	0	1
Sam 2007	Case-control	Lip and oral cavity cancer	1	1	1	1	0	1	0	0	0	1

Author	Study design	Health outcome	Geographically representative	Representative	Maximally adjusted	Adjusted for smoking, age, and sex	Adjusted for age and sex	Chewing tobacco temporality	Chewing tobacco product	Outcome assessment	Sub-population	Aggregate outcome definition
Sam 2010	Case-control	Lip and oral cavity cancer	1	1	1	0	0	1	0	0	0	1
Sankaranarayanan 1989	Case-control	Lip and oral cavity cancer	1	1	1	1	0	1	1	0	0	0
Sankaranarayanan 1989	Case-control	Lip and oral cavity cancer	1	1	1	1	1	1	1	0	0	0
Sankaranarayanan 1990	Case-control	Lip and oral cavity cancer	1	1	1	1	1	1	1	0	0	0
Sharma 2019	Case-control	Lip and oral cavity cancer	1	1	1	1	1	0	0	0	0	0
Sikdar 2004	Case-control	Lip and oral cavity cancer	1	1	1	1	1	1	0	0	0	0
Singh 2008	Case-control	Lip and oral cavity cancer	1	1	1	0	0	1	0	0	1	1
Singh 2009	Case-control	Lip and oral cavity cancer	1	1	1	1	1	1	0	0	0	1
Singh 2014	Case-control	Lip and oral cavity cancer	1	1	1	1	1	1	0	0	1	0
Soya 2007	Case-control	Lip and oral cavity cancer	1	1	1	1	0	1	0	0	0	1
Subapriya 2007	Case-control	Lip and oral cavity cancer	1	1	1	1	0	1	0	0	1	0
Subapriya 2007	Case-control	Lip and oral cavity cancer	1	1	1	1	0	1	1	0	1	0

Author	Study design	Health outcome	Geographically representative	Representative	Maximally adjusted	Adjusted for smoking, age, and sex	Adjusted for age and sex	Chewing tobacco temporality	Chewing tobacco product	Outcome assessment	Sub-population	Aggregate outcome definition
Wynder 1977	Case-control	Lip and oral cavity cancer	1	1	1	1	0	1	0	0	0	0
Wyss 2016	Case-control	Lip and oral cavity cancer	1	1	0	0	0	1	0	0	0	1
Wyss 2016	Case-control	Lip and oral cavity cancer	1	1	0	0	0	1	0	0	1	0
Wyss 2016	Case-control	Lip and oral cavity cancer	1	1	0	0	0	1	0	0	1	1
Yadav 2008	Case-control	Lip and oral cavity cancer	1	1	0	0	0	1	0	0	1	1
Yadav 2010	Case-control	Lip and oral cavity cancer	1	1	0	0	0	1	0	0	0	0
Znaor 2003	Case-control	Lip and oral cavity cancer	1	1	0	0	0	1	0	0	0	0
Akhtar 2016	Case-control	Nasopharynx cancer	1	1	0	0	0	1	0	0	0	1
Anuradha 2019	Case-control	Nasopharynx cancer	1	1	1	1	0	1	0	0	0	1
Arain 2015	Case-control	Nasopharynx cancer	1	1	1	1	1	0	1	0	1	1
Chelleng 2000	Case-control	Nasopharynx cancer	1	1	1	1	0	0	0	0	0	0
Choudhury 2015	Case-control	Nasopharynx cancer	1	1	1	1	1	1	1	0	0	1
Gajalakshmi 2012	Case-control	Nasopharynx cancer	1	1	0	0	0	1	0	1	1	1
Gupta 2020	Case-control	Nasopharynx cancer	1	1	1	1	0	1	0	0	0	1

Author	Study design	Health outcome	Geographically representative	Representative	Maximally adjusted	Adjusted for smoking, age, and sex	Adjusted for age and sex	Chewing tobacco temporality	Chewing tobacco product	Outcome assessment	Sub-population	Aggregate outcome definition
Jussawalla 1971	Case-control	Nasopharynx cancer	1	0	0	0	0	0	1	0	0	0
Notani 1988	Case-control	Nasopharynx cancer	1	1	1	0	0	0	0	0	0	1
Ruwali 2011	Case-control	Nasopharynx cancer	1	1	0	0	0	1	0	0	0	1
Sam 2007	Case-control	Nasopharynx cancer	1	1	1	1	0	1	0	0	0	1
Sam 2010	Case-control	Nasopharynx cancer	1	1	1	0	0	1	0	0	0	1
Singh 2008	Case-control	Nasopharynx cancer	1	1	1	0	0	1	0	0	1	1
Singh 2009	Case-control	Nasopharynx cancer	1	1	1	1	1	1	0	0	0	1
Soya 2007	Case-control	Nasopharynx cancer	1	1	1	1	0	1	0	0	0	1
Wyss 2016	Case-control	Nasopharynx cancer	1	1	0	0	0	1	0	0	0	1
Wyss 2016	Case-control	Nasopharynx cancer	1	1	0	0	0	1	0	0	1	1
Yadav 2008	Case-control	Nasopharynx cancer	1	1	0	0	0	1	0	0	1	1
Akhtar 2016	Case-control	Other pharynx cancer	1	1	0	0	0	1	0	0	0	1
Anantharaman 2014	Case-control	Other pharynx cancer	1	1	0	0	0	1	0	0	0	1
Anuradha 2019	Case-control	Other pharynx cancer	1	1	1	1	0	1	0	0	0	1
Arain 2015	Case-control	Other pharynx cancer	1	1	1	1	1	0	1	0	1	1

Author	Study design	Health outcome	Geographically representative	Representative	Maximally adjusted	Adjusted for smoking, age, and sex	Adjusted for age and sex	Chewing tobacco temporality	Chewing tobacco product	Outcome assessment	Sub-population	Aggregate outcome definition
Chang 2020	Case-control	Other pharynx cancer	1	1	0	0	0	1	1	0	0	1
Choudhury 2015	Case-control	Other pharynx cancer	1	1	1	1	1	1	1	0	0	1
Dholam 2016	Case-control	Other pharynx cancer	1	1	0	0	0	1	0	0	1	1
Dikshit 2000	Case-control	Other pharynx cancer	1	0	1	0	0	1	0	0	0	0
Gajalakshmi 2012	Case-control	Other pharynx cancer	1	1	0	0	0	1	0	1	1	1
Gupta 2020	Case-control	Other pharynx cancer	1	1	1	1	0	1	0	0	0	1
Herrero 2003	Case-control	Other pharynx cancer	1	1	1	1	0	1	1	0	0	0
Jafarey 1977	Case-control	Other pharynx cancer	1	0	0	0	0	0	0	0	1	1
Jayalekshmi 2013	Prospective cohort	Other pharynx cancer	1	0	0	0	0	0	0	0	0	0
Jussawalla 1971	Case-control	Other pharynx cancer	1	0	0	0	0	0	1	0	0	0
Kabat 1994	Case-control	Other pharynx cancer	1	1	0	0	0	0	0	0	0	1
Krishnarao 2015	Case-control	Other pharynx cancer	1	1	1	1	1	1	1	0	0	1
Mashberg 1993	Case-control	Other pharynx cancer	1	1	0	0	0	1	0	0	0	1
Notani 1988	Case-control	Other pharynx cancer	1	1	1	0	0	0	0	0	0	1
Rao 1999	Case-control	Other pharynx cancer	1	1	1	1	1	1	0	0	0	0

Author	Study design	Health outcome	Geographically representative	Representative	Maximally adjusted	Adjusted for smoking, age, and sex	Adjusted for age and sex	Chewing tobacco temporality	Chewing tobacco product	Outcome assessment	Sub-population	Aggregate outcome definition
Ruwali 2009	Case-control	Other pharynx cancer	1	1	1	1	1	1	0	0	0	1
Ruwali 2011	Case-control	Other pharynx cancer	1	1	0	0	0	1	0	0	0	1
Sam 2007	Case-control	Other pharynx cancer	1	1	1	1	0	1	0	0	0	1
Sam 2010	Case-control	Other pharynx cancer	1	1	1	0	0	1	0	0	0	1
Sapkota 2007	Case-control	Other pharynx cancer	1	1	0	0	0	1	0	0	1	0
Singh 2008	Case-control	Other pharynx cancer	1	1	1	0	0	1	0	0	1	1
Singh 2009	Case-control	Other pharynx cancer	1	1	1	1	1	1	0	0	0	1
Soya 2007	Case-control	Other pharynx cancer	1	1	1	1	0	1	0	0	0	1
Wasnik 1998	Case-control	Other pharynx cancer	1	1	0	0	0	1	0	0	0	0
Wyss 2016	Case-control	Other pharynx cancer	1	1	0	0	0	1	0	0	0	1
Wyss 2016	Case-control	Other pharynx cancer	1	1	0	0	0	1	0	0	1	0
Wyss 2016	Case-control	Other pharynx cancer	1	1	0	0	0	1	0	0	1	1
Yadav 2008	Case-control	Other pharynx cancer	1	1	0	0	0	1	0	0	1	1
Znaor 2003	Case-control	Other pharynx cancer	1	1	1	1	1	1	0	0	0	0
Agashe 2013	Case-control	Stroke	1	1	1	1	0	0	1	0	0	N/A
Gajalakshmi 2015	Case-control	Stroke	1	1	0	0	0	1	0	0	0	N/A

Author	Study design	Health outcome	Geographically representative	Representative	Maximally adjusted	Adjusted for smoking, age, and sex	Adjusted for age and sex	Chewing tobacco temporality	Chewing tobacco product	Outcome assessment	Sub-population	Aggregate outcome definition
Mateen 2012	Case-control	Stroke	1	1	1	1	0	1	1	0	0	N/A

Bias covariates were eligible for testing in a model if there were two or more observations for each coded value of the covariate. If two or more bias covariates had the same values across all the observations, only one would be kept as eligible for testing with the covariates that align with GRADE criteria for bias being prioritized over the ones specific to chewing tobacco.

Table S10: Bias Covariates eligible for testing for each health outcome

Health outcome	Eligible bias covariates	Selected bias covariates
Stroke	Chewing tobacco temporality	None
Lip and oral cavity cancer	Aggregate outcome definition; Outcome assessment; Geographically representative; Representative; Maximally adjusted; Adjusted for smoking, age, and sex; Adjusted for age and sex; Sub-population; Chewing tobacco temporality; Chewing tobacco product	Chewing tobacco product; Sub-population
Esophageal cancer	Aggregate outcome definition; Outcome assessment; Representative; Maximally adjusted; Adjusted for smoking, age, and sex; Adjusted for age and sex; Sub-population; Chewing tobacco temporality; Chewing tobacco product	Maximally adjusted; Adjusted for smoking, age, and sex
Larynx cancer	Aggregate outcome definition; Outcome assessment; Geographically representative; Representative; Maximally adjusted; Adjusted for smoking, age, and sex; Adjusted for age and sex; Sub-population; Chewing tobacco temporality; Chewing tobacco product	Aggregate outcome definition; Adjusted for age and sex
Nasopharynx cancer	Aggregate outcome definition; Outcome assessment; Maximally adjusted; Adjusted for smoking, age, and sex; Adjusted for age and sex; Sub-population; Chewing	Maximally adjusted; Adjusted for age and sex

	tobacco temporality; Chewing tobacco product	
Other pharynx cancer	Aggregate outcome definition; Outcome assessment; Representative; Maximally adjusted; Adjusted for smoking, age, and sex; Adjusted for age and sex; Sub-population; Chewing tobacco temporality; Chewing tobacco product	Aggregate outcome definition; Adjusted for age and sex
Ischemic heart disease	Geographically representative; Representative; Maximally adjusted; Adjusted for smoking, age, and sex; Chewing tobacco temporality; Chewing tobacco product	None

Section 3.4: Detailed methods for estimating the relative risk of 8 health outcomes

The methods used to estimate the relative risk within the Burden of Proof framework have been described in detail elsewhere[4–9]. Here, we present some specific details that are particularly relevant to chewing tobacco relative risk estimations that were not covered in as much detail in the manuscript due to a lack of available space.

Within GBD, chewing tobacco exposure is estimated as a dichotomous risk factor in which individuals are either exposed (use chewing tobacco) or are not (do not use chewing tobacco)[2]. As a result, all of our risk-outcome pairs associated with chewing tobacco were evaluated as binary risk-outcome associations to produce compatible results for future incorporation in GBD. Furthermore, dose-response data for chewing tobacco is extremely limited, which would hinder our ability to create accurate dose-response curves if it were treated as a continuous risk factor. Similarly, we did not estimate sex-specific relative risks or any other disaggregated measures of risk because these estimates will be broadly applied within the GBD framework and because of the limited data available. Furthermore, it is unlikely the mechanism through which chewing tobacco influences an individual's risk of a given health outcome is impacted by their sex or other such characteristics, so a global relative risk was deemed appropriate given these constraints.

Since the meta-regression tool draws upon the uncertainty surrounding each observation to inform its estimate, we had to address the fact that three eligible observations for our head and neck cancer models did not report an associated uncertainty. We filled in these missing values by using the 98th percentile log-space standard error reported in the other head and neck cancer studies. This approach reflects a conservative estimation of uncertainty for these three data points and is not expected to influence our model results substantially.

For very data-sparse risk-outcome pairs, like chewing tobacco and stroke, the Burden of Proof approach applies the Fisher Scoring correction to gamma, which uses a quantile of our between-study heterogeneity that is sensitive to study characteristics and number of studies to address the potential underestimation of gamma in these conditions.

Table S11: MR-BRT model specifications by risk-outcome pair

Health outcome	Type of risk	Number of draws	Percent trimmed	Pre-selected covariates
Stroke	Dichotomous	1000	0%	None
Lip and oral cavity cancer	Dichotomous	1000	10%	None
Esophageal cancer	Dichotomous	1000	10%	None
Larynx cancer	Dichotomous	1000	10%	None
Nasopharynx cancer	Dichotomous	1000	10%	None
Other pharynx cancer	Dichotomous	1000	10%	None
Ischemic heart disease	Dichotomous	1000	0%	None

Section 3.5: Quantifying between-study heterogeneity

The gamma solution and its standard deviation (SD) quantifies the degree of heterogeneity observed in the included observations, while the SD quantifies the uncertainty around gamma, both of which contribute to our final conservative BPRF and RR uncertainty estimates. Within all dichotomous risk factors evaluated using the BPRF methodology, gamma solution values range from 0 to 0.59 with a mean of 0.07 (SD: 0.15).

Table S12: Gamma solution for each risk-outcome pair quantifying between-study heterogeneity

Health outcome	Gamma solution (SD)
Stroke	4.7×10^{-06} (0.0076)
Lip and oral cavity cancer	0.53 (0.11)
Esophageal cancer	0.092 (0.051)
Larynx cancer	0.39 (0.14)
Nasopharynx cancer	0.34 (0.16)
Other pharynx cancer	0.43 (0.13)
Ischemic heart disease	0.24 (0.15)

Section 4: Sensitivity Analyses

We conducted a number of sensitivity analyses to evaluate the robustness of our primary results with regard to changes in model parameters and data point inclusion. Each sensitivity analysis with more than 10 observations was conducted with and without 10% trimming, while observations with fewer than 10 observations were only feasible to conduct without trimming. For outcomes with sufficient data points to include 10% trimming in the primary analysis, we conducted an additional sensitivity analysis with the original dataset and other parameters but omitting the trimming of any data points. It was only feasible to run these analyses when data point restrictions still allowed for the inclusion of more than two observations. The sensitivity analyses included:

- **No covariates included:** Using no bias covariates to account for potential systematic biases. In this analysis, no adjustments would be made to observations to account for the patterns of significant biases detected in the covariate selection algorithm. Other parameters and the included observations were kept the same.
- **Removed data with <5 exposed/unexposed cases or controls**: We removed observations with very small samples in the traditional four-by-four table used to derive measures of excess risk. Specifically, we omitted observations that had fewer than five tobacco chewers with the outcome, fewer than five tobacco chewers without the outcome, fewer than five non-chewers with the outcome, or fewer than five non-chewers without the outcome. This analysis examines the potential impact of small studies on our results. Other parameters were kept the same.
- **Used only data points that defined tobacco chewers as current users:** Using only data points where the exposed group was reported to be current chewers (versus a reference group of non-chewers). This exposure temporality reflects the closest match to our gold-standard exposure definition and excludes observations that were estimated using ever chewers as the exposed group and never chewers as the reference group. Other parameters were kept the same.
- **Removed data points using aggregate outcome definitions:** This analysis was only relevant for the head and neck cancer outcomes. For these models, we removed observations that used an aggregate outcome definition and kept only observations that were specific to the head and neck cancer outcome in question. Other parameters were kept the same.
- **Used only male-specific data points:** We restricted the dataset to only observations that were estimated from a male-only sample. To ensure we capture the full impact of sex-specific observations, we also included male-specific observations that had not been selected for the primary analysis in favor of observations derived from an analytic sample with both males and females. Other parameters were kept the same.
- **Used only female-specific data points:** We restricted the dataset to only observations that were estimated from a female-only sample. To ensure we capture the full impact of sex-specific observations, we also included female-specific observations that had not been selected for the primary analysis in favor of observations derived from an analytic sample with both males and females. Other parameters were kept the same.
- **Used only data points from studies conducted in Asian countries:** We restricted the dataset to only observations that were estimated from studies conducted in Asian countries, which may have greater homogeneity in the types of chewing tobacco products used. Other parameters were kept the same.
- **Used only data points from non-smoking samples:** We restricted the dataset to only observations that were estimated from a sample of non-smokers. Other parameters were kept the same.
- **Did not apply any downweighing based on overlapping samples:** We removed the adjustment factors used to account for effect sizes derived from non-mutually exclusive analytical samples to examine the impact of this adjustment on our analyses. In this sensitivity analysis, all observations are equally weighted in the input dataset.

Section 4.1: Sensitivity analyses for the relationship between chewing tobacco and stroke

Table S13: Results of sensitivity analyses for chewing tobacco and stroke

Sensitivity Analysis	% trimming	RR (95% UI without γ)	RR (95% UI with γ)	BPRF	ROS	Star rating	Pub. bias	No. of studies	Selected covariates
Primary analysis	0%	1.46 (1.28-1.68)	1.46 (1.11-1.93)	1.16	0.07	2	No	3	None
No covariates included	0%	1.46 (1.28-1.68)	1.46 (1.11-1.93)	1.16	0.07	2	No	3	None
Removed data with <5 exposed/unexposed cases or controls	0%	1.46 (1.28-1.68)	1.46 (1.11-1.93)	1.16	0.07	2	No	3	None
Used only male-specific data points	0%	1.70 (1.10-2.63)	1.70 (0.67-4.35)	0.77	-0.13	1	No	2	None
Did not apply any downweighing based on overlapping samples	0%	1.47 (1.28-1.68)	1.47 (1.11-1.94)	1.16	0.08	2	No	3	None
Used only data points from studies conducted in Asian countries	0%	1.46 (1.28-1.68)	1.46 (1.11-1.93)	1.16	0.07	2	No	3	None

Section 4.2: Sensitivity analyses for the relationship between chewing tobacco and ischemic heart disease

Table S14: Results of sensitivity analyses for chewing tobacco and ischemic heart disease

Sensitivity Analysis	% interested	RR (95% UI with γ)	RR (95% UI without γ)	BPRF	ROS	Star rating	Pub. bias	No. of studies	Selected covariates
Primary analysis	0%	1.30 (0.88-1.92)	1.30 (0.29-5.83)	N/A	N/A	N/A	No	8	None
No covariates included	0%	1.30 (0.88-1.92)	1.30 (0.29-5.83)	N/A	N/A	N/A	No	8	None
Removed data with <5 exposed/unexposed cases or controls	0%	1.35 (0.88-2.06)	1.35 (0.27-6.68)	N/A	N/A	N/A	No	7	None
Used only data points that defined tobacco chewers as current users	0%	1.07 (0.86-1.33)	1.07 (0.59-1.92)	N/A	N/A	N/A	No	3	None
Used only data points from studies conducted in Asian countries	0%	1.31 (0.64-2.68)	1.31 (0.12-14.39)	N/A	N/A	N/A	No	5	None
Did not apply any downweighing based on overlapping samples	0%	1.29 (0.87-1.90)	1.29 (0.28-5.89)	N/A	N/A	N/A	No	8	None
Used only data points from non-smoking samples	0%	1.17 (0.97-1.40)	1.71 (0.81-1.69)	N/A	N/A	N/A	No	3	None

Section 4.3: Sensitivity analyses for the relationship between chewing tobacco and esophageal cancer

Table S15: Results of sensitivity analyses for chewing tobacco and esophageal cancer

Sensitivity Analysis	% trimming	RR (95% UI without γ)	RR (95% UI with γ)	BPRF	ROS	Star rating	Pub. bias	No. of studies	Selected covariates
Primary analysis	10%	2.14 (1.77-2.57)	2.14 (0.89-5.15)	1.02	0.01	2	No	22	Maximally adjusted, Adjusted for smoking, age, and sex
	0%	2.57 (1.75-3.78)	2.57 (0.27-24.07)	0.39	-0.47	1	No	22	Maximally adjusted, Adjusted for smoking, age, and sex
No covariates included	10%	2.13 (1.77-2.57)	2.13 (0.88-5.15)	1.02	0.01	2	No	22	None
	0%	2.57 (1.75-3.78)	2.57 (0.27-24.07)	0.39	-0.47	1	No	22	None
Removed data with <5 exposed/unexposed cases or controls	10%	2.06 (1.69-2.51)	2.06 (0.77-5.52)	0.9	-0.05	1	No	22	Maximally adjusted, Adjusted for smoking, age, and sex
	0%	2.61 (1.76-3.85)	2.61 (0.27-24.86)	0.39	-0.47	1	No	22	Maximally adjusted, Adjusted for smoking, age, and sex
Removed data points using aggregate outcome definitions	10%	2.09 (1.72-2.55)	2.09 (0.84-5.23)	0.97	-0.02	1	No	20	Maximally adjusted, Adjusted for

									smoking, age, and sex
	0%	2.11 (1.61-2.75)	2.11 (0.51-8.76)	0.64	-0.23	1	No	20	Maximally adjusted, Adjusted for smoking, age, and sex
Used only data points that defined tobacco chewers as current users	10%	2.49 (2.08-2.98)	2.49 (1.54-4.05)	1.66	0.25	3	No	8	Chewing tobacco product, Maximally adjusted, Representative
	0%	2.08 (1.45-2.97)	2.08 (0.52-8.26)	0.65	-0.21	1	No	8	Chewing tobacco product, Maximally adjusted
Used only male-specific data points	0%	1.64 (1.21-2.23)	1.64 (0.48-5.63)	0.58	-0.27	1	No	9	Maximally adjusted, Adjusted for smoking, age, and sex
Used only female-specific data points	0%	2.14 (1.35-3.4)	2.14 (0.52-8.87)	0.65	-0.22	1	No	4	Maximally adjusted
Used only data points from studies conducted in Asian countries	10%	2.1 (1.72-2.56)	2.1 (0.8-5.5)	0.94	-0.03	1	No	21	Sub-population
	0%	2.67 (1.79-3.98)	2.67 (0.27-25.9)	0.40	-0.46	1	No	21	Maximally adjusted, Adjusted for smoking, age, and sex
Did not apply any downweighing based on overlapping samples	10%	2.19 (1.78-2.7)	2.19 (0.79-6.1)	0.93	-0.04	1	No	22	Maximally adjusted, Adjusted for smoking, age,

									and sex, Aggregate outcome definition
	0%	2.58 (1.76-3.8)	2.58 (0.27-24.28)	0.39	-0.47	1	No	22	Maximally adjusted, Adjusted for smoking, age, and sex
Used only data points from non-smoking samples	0%	3.29 (1.58-6.86)	3.29 (0.32-34.17)	0.46	-0.39	1	No	4	None

Section 4.4: Sensitivity analyses for the relationship between chewing tobacco and lip and oral cavity cancer

Table S16: Results of sensitivity analyses for chewing tobacco and lip and oral cavity cancer

Sensitivity Analysis	% trimming	RR (95% UI without γ)	RR (95% UI with γ)	BPRF	ROS	Star rating	Pub. bias	No. of studies	Selected covariates
Primary analysis	10%	3.64 (3-4.41)	3.64 (0.66-19.95)	0.87	-0.07	1	No	70	Chewing tobacco product, Sub-population
	0%	3.81 (3.06-4.76)	3.81 (0.5-29.3)	0.69	-0.19	1	No	70	Chewing tobacco product, Sub-population
No covariates included	10%	3.64 (3-4.41)	3.64 (0.66-19.96)	0.87	-0.07	1	No	70	None
	0%	3.81 (3.06-4.76)	3.81 (0.5-29.3)	0.69	-0.19	1	No	70	None
Removed data with <5 exposed/unexposed cases or controls	10%	3.61 (2.98-4.38)	3.61 (0.68-19.22)	0.89	-0.06	1	No	64	Chewing tobacco product, Sub-population
	0%	3.71 (2.97-4.64)	3.71 (0.5-27.5)	0.69	-0.18	1	No	64	Chewing tobacco product, Sub-population
Removed data points using aggregate outcome definitions	10%	3.96 (3.09-5.06)	3.96 (0.62-25.43)	0.83	-0.09	1	No	49	Chewing tobacco product, Adjusted for age and sex, Geographically representative, Adjusted for age, sex, and smoking, Sub-population
	0%	3.89 (2.97-5.11)	3.89 (0.45-33.46)	0.64	-0.22	1	No	49	Chewing tobacco product, Sub-population, Adjusted for age

									and sex, Adjusted for age, sex, and smoking
Used only data points that defined tobacco chewers as current users	10%	3.62 (2.44-5.35)	3.62 (0.53-24.86)	0.72	-0.17	1	No	17	Adjusted for age and sex, Sub-population, Representative
	0%	3.47 (2.39-5.04)	3.47 (0.53-22.7)	0.72	-0.17	1	No	17	Adjusted for age and sex, Sub-population
Used only male-specific data points	10%	3.42 (2.53-4.62)	3.42 (0.57-20.35)	0.77	-0.13	1	No	24	Aggregate outcome definition, Chewing tobacco product
	0%	3.35 (2.42-4.62)	3.35 (0.47-23.76)	0.65	-0.22	1	No	24	Aggregate outcome definition, Chewing tobacco product
Used only female-specific data points	10%	5.45 (3.21-9.27)	5.45 (0.55-53.72)	0.8	-0.11	1	No	11	Adjusted for age and sex, Exposure temporality
	0%	6.6 (3.55-12.3)	6.6 (0.4-109.58)	0.63	-0.23	1	No	11	Adjusted for age and sex, Exposure temporality
Used only data points from studies conducted in Asian countries	10%	3.98 (3.28-4.85)	3.98 (0.78-20.44)	1.01	0.01	2	No	63	Adjusted for age and sex, Chewing tobacco product
	0%	4.18 (3.32-5.25)	4.18 (0.56-31.45)	0.77	-0.13	1	No	63	Adjusted for age and sex, Chewing tobacco product, Sub-population
Did not apply any downweighing based on overlapping samples	10%	3.73 (3.08-4.53)	3.73 (0.66-21.08)	0.87	-0.07	1	No	70	Adjusted for age and sex, Chewing tobacco product, Sub-population
	0%	3.81 (3.06-4.75)	3.81 (0.5-28.94)	0.70	-0.18	1	No	70	Chewing tobacco product, Sub-population

Used only data points from non-smoking samples	10%	3.96 (2.77-5.64)	3.96 (0.84-18.59)	1.08	0.04	2	No	15	None
	0%	4 (2.55-6.28)	4 (0.43-37.53)	0.61	-0.25	1	No	15	None

Section 4.5: Sensitivity analyses for the relationship between chewing tobacco and laryngeal cancer

Table S17: Results of sensitivity analyses for chewing tobacco and laryngeal cancer

Sensitivity Analysis	% trimming	RR (95% UI without γ)	RR (95% UI with γ)	BPRF	ROS	Star rating	Pub. bias	No. of studies	Selected covariates
Primary analysis	10%	2.66 (1.98-3.57)	2.66 (0.52-13.63)	0.68	-0.20	1	No	24	Aggregate outcome definition, Adjusted for age and sex
	0%	2.78 (1.91-4.06)	2.78 (0.29-27.11)	0.41	-0.44	1	No	24	Aggregate outcome definition, Adjusted for age and sex
No covariates included	10%	2.66 (1.98-3.57)	2.66 (0.52-13.63)	0.68	-0.20	1	No	24	None
	0%	2.78 (1.91-4.06)	2.78 (0.29-27.11)	0.41	-0.44	1	No	24	None
Removed data with <5 exposed/unexposed cases or controls	10%	2.58 (1.91-3.48)	2.58 (0.51-13.07)	0.66	-0.21	1	No	22	Aggregate outcome definition, Adjusted for age and sex
	0%	2.72 (1.83-4.03)	2.72 (0.27-27.53)	0.39	-0.47	1	No	22	Aggregate outcome definition, Adjusted for age and sex
Removed data points using aggregate outcome definitions	10%	1.16 (0.99-1.35)	1.16 (0.76-1.78)	N/A	N/A	N/A	No	9	Chewing tobacco product, Representative
	0%	1.6 (1.02-2.52)	1.6 (0.25-10.33)	0.34	-0.55	1	No	9	Chewing tobacco product, Representative
Used only data points that defined tobacco chewers as current users	0%	1.80 (0.97-3.35)	1.80 (0.25-13.22)	N/A	N/A	N/A	No	5	Chewing tobacco product

Used only male-specific data points	10%	1.75 (1.3-2.35)	1.75 (0.54-5.65)	0.65	-0.21	1	No	11	Aggregate outcome definition, Sub-population
	0%	2.28 (1.51-3.44)	2.28 (0.37-14.13)	0.49	-0.35	1	No	11	Aggregate outcome definition, Sub-population
Used only data points from studies conducted in Asian countries	10%	2.95 (2.17-4.01)	2.95 (0.6-14.44)	0.78	-0.13	1	No	21	Adjusted for age and sex, Aggregate outcome definition
	0%	3.02 (1.98-4.58)	3.02 (0.28-32.93)	0.41	-0.45	1	No	21	Adjusted for age and sex, Aggregate outcome definition
Did not apply any downweighing based on overlapping samples	10%	2.65 (1.98-3.56)	2.65 (0.52-13.61)	0.67	-0.2	1	No	24	Aggregate outcome definition, Adjusted for age and sex
	0%	2.78 (1.9-4.05)	2.78 (0.29-27.05)	0.41	-0.44	1	No	24	Aggregate outcome definition, Adjusted for age and sex
Used only data points from non-smoking samples	0%	4.22 (2.85-6.24)	4.22 (1.2-14.83)	1.47	0.19	3	No	5	Self-reported outcome

Section 4.6: Sensitivity analyses for the relationship between chewing tobacco and nasopharynx cancer

Table S18: Results of sensitivity analyses for chewing tobacco and nasopharynx cancer

Sensitivity Analysis	% trimming	RR (95% UI without γ)	RR (95% UI with γ)	BPRF	ROS	Star rating	Pub. bias	No. of studies	Selected covariates
Primary analysis	10%	2.5 (1.79-3.49)	2.5 (0.49-12.66)	0.64	-0.22	1	No	17	Maximally adjusted, Adjusted for age and sex
	0%	3.13 (1.95-5.04)	3.13 (0.25-38.63)	0.38	-0.48	1	No	17	Maximally adjusted, Adjusted for age and sex
No covariates included	10%	2.5 (1.79-3.49)	2.5 (0.49-12.66)	0.64	-0.22	1	No	17	None
	0%	3.13 (1.95-5.04)	3.13 (0.25-38.63)	0.38	-0.48	1	No	17	None
Removed data with <5 exposed/unexposed cases or controls	10%	2.53 (1.79-3.57)	2.53 (0.48-13.2)	0.63	-0.23	1	No	16	Maximally adjusted, Adjusted for age and sex
	0%	3.21 (1.96-5.26)	3.21 (0.25-41.88)	0.37	-0.49	1	No	16	Maximally adjusted, Adjusted for age and sex
Used only data points that defined tobacco chewers as current users	0%	1.38 (0.89-2.12)	1.38 (0.42-4.45)	N/A	N/A	N/A	No	4	None
Used only male-specific data points	0%	2.51 (1.51-4.18)	2.51 (0.36-17.49)	0.49	-0.35	1	No	7	Sub-population, Adjusted for smoking, age, and sex, Chewing tobacco product

Used only female-specific data points	0%	1.96 (1-3.85)	1.96 (0.35-10.89)	N/A	N/A	N/A	No	2	Maximally adjusted
Used only data points from studies conducted in Asian countries	10%	2.5 (1.79-3.5)	2.5 (0.54-11.59)	0.69	-0.18	1	No	16	Adjusted for age and sex, Exposure temporality, Chewing tobacco product
	0%	3.4 (2.11-5.49)	3.4 (0.29-40.17)	0.43	-0.42	1	No	16	Adjusted for age and sex, Exposure temporality
Did not apply any downweighing based on overlapping samples	10%	2.49 (1.78-3.48)	2.49 (0.48-12.78)	0.63	-0.23	1	No	17	Maximally adjusted, Adjusted for age and sex, Exposure temporality, Chewing tobacco product
	0%	3.13 (1.94-5.03)	3.13 (0.25-38.7)	0.38	-0.49	1	No	17	Maximally adjusted, Adjusted for age and sex, Exposure temporality, Chewing tobacco product
Used only data points from non-smoking samples	0%	2.96 (1.67-5.25)	2.96 (0.39-22.42)	0.54	-0.31	1	No	6	None

Section 4.7: Sensitivity analyses for the relationship between chewing tobacco and other pharynx cancer

Table S19: Results of sensitivity analyses for chewing tobacco and other pharynx cancer

Sensitivity Analysis	% trimming	RR (95% UI without γ)	RR (95% UI with γ)	BPRF	ROS	Star rating	Pub. bias	No. of studies	Selected covariates
Primary analysis	10%	2.33 (1.8-3.01)	2.33 (0.45-12.04)	0.59	-0.27	1	No	31	Aggregate outcome definition, Adjusted for age and sex
	0%	2.75 (2-3.78)	2.75 (0.32-23.32)	0.46	-0.39	1	No	31	Adjusted for age and sex, Aggregate outcome definition
No covariates included	10%	2.33 (1.8-3.01)	2.33 (0.45-12.04)	0.59	-0.27	1	No	31	None
	0%	2.75 (2-3.78)	2.75 (0.32-23.32)	0.46	-0.39	1	No	31	None
Removed data with <5 exposed/unexposed cases or controls	10%	2.36 (1.81-3.08)	2.36 (0.44-12.65)	0.58	-0.27	1	No	29	Adjusted for age and sex, Aggregate outcome definition
	0%	2.82 (2.02-3.93)	2.82 (0.32-25.05)	0.45	-0.4	1	No	29	Aggregate outcome definition, Adjusted for age and sex
Removed data points using aggregate outcome definitions	10%	1.45 (1.06-1.98)	1.45 (0.43-4.85)	0.52	-0.32	1	No	9	Maximally adjusted, Adjusted for smoking, age, and sex
	0%	1.63 (1.03-2.59)	1.63 (0.24-11.15)	0.33	-0.56	1	No	9	Maximally adjusted,

									Adjusted for smoking, age, and sex
Used only data points that defined tobacco chewers as current users	10%	1.73 (1-2.98)	1.73 (0.29-10.41)	N/A	N/A	N/A	No	6	Maximally adjusted, Adjusted for smoking, age, and sex
	0%	1.84 (1.18-2.89)	1.84 (0.38-8.85)	0.49	-0.35	1	No	6	Adjusted for smoking, age, and sex, Maximally adjusted
Used only male-specific data points	10%	1.66 (1.27-2.19)	1.66 (0.48-5.81)	0.58	-0.27	1	No	14	Aggregate outcome definition, Adjusted for age, sex, and smoking
	0%	1.8 (1.28-2.53)	1.8 (0.35-9.3)	0.45	-0.40	1	No	14	Aggregate outcome definition, Adjusted for age, sex, and smoking
Used only female-specific data points	0%	1.96 (1-3.85)	1.96 (0.35-10.89)	N/A	N/A	N/A	No	2	Maximally adjusted
Used only data points from studies conducted in Asian countries	10%	2.48 (1.89-3.27)	2.48 (0.47-13.16)	0.61	-0.24	1	No	27	Adjusted for age and sex, Aggregate outcome definition, Adjusted for age, sex, and smoking
	0%	2.99 (2.12-4.22)	2.99 (0.33-26.9)	0.47	-0.37	1	No	27	Adjusted for age and sex,

									Aggregate outcome definition, Adjusted for age, sex, and smoking
Did not apply any downweighing based on overlapping samples	10%	2.33 (1.8-3.01)	2.33 (0.45-12.06)	0.59	-0.27	1	No	31	Adjusted for age and sex, Aggregate outcome definition
	0%	2.75 (2-3.78)	2.75 (0.32-23.34)	0.46	-0.39	1	No	31	Adjusted for age and sex, Aggregate outcome definition
Used only data points from non-smoking samples	10%	4.38 (3.06-6.27)	4.38 (1.09-17.58)	1.36	0.15	3	No	10	Self-reported outcome
	0%	4.37 (2.76-6.93)	4.37 (0.61-31.43)	0.84	-0.09	1	No	10	Self-reported outcome

Section 4.8: Study characteristics and observations for sex-specific sensitivity analyses

The following tables depict study characteristics and observations that were not included in the primary analysis in favor of their both-sexes counterpart reported in the same studies. These observations were included in the sex-specific sensitivity analyses in addition to the sex-specific observations included in the primary analysis.

Table S20: Study characteristics for male-specific sensitivity analysis

Author	Year	Health outcome	Study name	Location	Study design	Sex	Age start	Age end	Exposure assessment	Outcome assessment method	Endpoint	Sample size	Exposed	Follow-up (years)	Person-years	Events	Cases	Controls	Control pool	Smoking status	Chewing tobacco type
Sankaranarayanan	1991	Esophageal cancer	N/A	Kerala	Case-control	Males	18	99	Self-report	Physician diagnosis; Biomarker	Morbidity	NA	NA	N/A	NA	NA	189	541	Hospital	Any smoking status	Pan With Tobacco
Muwonge	2008	Lip and oral cavity cancer	N/A	Kerala	Nested case-control	Males	35	99	Self-report	Physician diagnosis; Biomarker	Incidence	NA	NA	8	NA	NA	163	815	Community	Any smoking status	Exact
Nandakumar	1990	Lip and oral cavity cancer	N/A	Karnataka	Case-control	Males	15	99	Self-report	Biomarker	Morbidity	NA	NA	N/A	NA	NA	115	115	Hospital	Any smoking status	Pan With Tobacco
Ray	2013	Lip and oral cavity cancer	N/A	West Bengal	Case-control	Males	10	99	Self-report	Physician diagnosis; Biomarker	Morbidity	NA	NA	N/A	NA	NA	NA	NA	Hospital	Any smoking status	Exact
Sankaranarayanan	1989	Lip and oral cavity cancer	N/A	Kerala	Case-control	Males	18	99	Self-report	Physician diagnosis; Biomarker	Morbidity	NA	NA	N/A	NA	NA	150	310	Hospital	Any smoking status	Pan With Tobacco
Sankaranarayanan	1990	Lip and oral cavity cancer	N/A	Kerala	Case-control	Males	15	99	Self-report	Administrative medical records or disease registries	Morbidity	NA	NA	N/A	NA	NA	240	542	Hospital	Any smoking status	Pan With Tobacco
Sankaranarayanan	1989	Lip and oral cavity cancer	N/A	Kerala	Case-control	Males	18	99	Self-report	Administrative medical records or disease registries	Morbidity	NA	NA	N/A	NA	NA	106	541	Hospital	Any smoking status	Pan With Tobacco
Gajalakshmi	2015	Stroke (unspecified)	N/A	Tamil Nadu	Case-control	Males	35	69	Self-report	Death certificates	Mortality	NA	NA	N/A	NA	NA	197	23,254	Community	Never smokers	Exact
Gajalakshmi	2015	Stroke (unspecified)	N/A	Tamil Nadu	Case-control	Males	35	69	Self-report	Death certificates	Mortality	NA	NA	N/A	NA	NA	284	138,928	Community	Never smokers	Exact

N/A, not available

Table S21: Study characteristics for female-specific sensitivity analysis

Author	Year	Health outcome	Study name	Location	Study design	Sex	Age start	Age end	Exposure assessment	Outcome assessment method	Endpoint	Sample size	Exposed	Follow-up (years)	Person-years	Events	Cases	Controls	Control pool	Smoking status	Chewing tobacco type
Sankaranarayanan	1991	Esophageal cancer	N/A	Kerala	Case-control	Females	18	99	Self-report	Physician diagnosis; Biomarker	Morbidity	NA	NA	N/A	NA	NA	55	345	Hospital	Any smoking status	Pan With Tobacco
Muwonge	2008	Lip and oral cavity cancer	N/A	Kerala	Nested case-control	Females	35	99	Self-report	Physician diagnosis; Biomarker	Incidence	NA	NA	8	NA	NA	119	595	Community	Any smoking status	Exact
Nandakumar	1990	Lip and oral cavity cancer	N/A	Karnataka	Case-control	Females	15	99	Self-report	Biomarker	Morbidity	NA	NA	N/A	NA	NA	233	233	Hospital	Any smoking status	Pan With Tobacco
Ray	2013	Lip and oral cavity cancer	N/A	West Bengal	Case-control	Females	10	99	Self-report	Physician diagnosis; Biomarker	Morbidity	NA	NA	N/A	NA	NA	NA	NA	Hospital	Any smoking status	Exact
Sankaranarayanan	1989	Lip and oral cavity cancer	N/A	Kerala	Case-control	Females	18	99	Self-report	Physician diagnosis; Biomarker	Morbidity	NA	NA	N/A	NA	NA	75	345	Hospital	Any smoking status	Pan With Tobacco
Sankaranarayanan	1990	Lip and oral cavity cancer	N/A	Kerala	Case-control	Females	15	99	Self-report	Administrative medical records or disease registries	Morbidity	NA	NA	N/A	NA	NA	162	345	Hospital	Any smoking status	Pan With Tobacco
Sankaranarayanan	1989	Lip and oral cavity cancer	N/A	Kerala	Case-control	Females	18	99	Self-report	Administrative medical records or disease registries	Morbidity	NA	NA	N/A	NA	NA	66	138	Hospital	Any smoking status	Pan With Tobacco

N/A, not available

Table S22: Additional data inputs for sex-specific sensitivity analyses

Author	Health outcome	Sex	Log effect size	Standard error of the log effect size
Sankaranarayanan 1991	Esophageal cancer	Female	-0.075	0.28

Muwonge 2008	Lip and oral cavity cancer	Female	2.25	0.33
Sankaranarayanan 1989	Lip and oral cavity cancer	Female	1.85	0.36
Sankaranarayanan 1990	Lip and oral cavity cancer	Female	1.97	0.27
Nandakumar 1990	Lip and oral cavity cancer	Female	3.23	0.42
Sankaranarayanan 1989	Lip and oral cavity cancer	Female	2.41	0.44
Ray 2013	Lip and oral cavity cancer	Female	2.17	0.38
Sankaranarayanan 1991	Esophageal cancer	Male	0.30	0.17
Muwonge 2008	Lip and oral cavity cancer	Male	0.99	0.21
Sankaranarayanan 1989	Lip and oral cavity cancer	Male	1.29	0.21
Sankaranarayanan 1990	Lip and oral cavity cancer	Male	2.38	0.20
Nandakumar 1990	Lip and oral cavity cancer	Male	1.28	0.39
Sankaranarayanan 1989	Lip and oral cavity cancer	Male	2.22	0.27
Ray 2013	Lip and oral cavity cancer	Male	1.61	0.32
Gajalakshmi 2015	Stroke	Male	0.79	0.16
Gajalakshmi 2015	Stroke	Male	0.10	0.23

Section 5: GATHER and PRISMA checklists

Section 5.1: PRISMA

Table S23: PRISMA 2020 Abstract Checklist

Section and Topic	Item #	Checklist item	Reported (Yes/No)
**TITLE**			
Title	1	Identify the report as a systematic review.	Identified in the abstract but not in the title. The title reflects the review and meta-analysis methodology used.
**BACKGROUND**			
Objectives	2	Provide an explicit statement of the main objective(s) or question(s) the review addresses.	Yes
**METHODS**			
Eligibility criteria	3	Specify the inclusion and exclusion criteria for the review.	Briefly, but more detail provided in the main text and Supplementary Information 1.2.
Information sources	4	Specify the information sources (e.g. databases, registers) used to identify studies and the date when each was last searched.	The fact that three databases were reviewed is mentioned; Space limitation required us to describe search details in the main text and Supplementary Information 1.1 and 3.1.
Risk of bias	5	Specify the methods used to assess risk of bias in the included studies.	Yes, briefly as incorporated in the burden of proof meta-analytic approach; More detail provided in the main text and Supplementary Information 3.2.
Synthesis of results	6	Specify the methods used to present and synthesise results.	Yes, briefly; More detail provided in the main text and Supplementary Information 3.3.
**RESULTS**			
Included studies	7	Give the total number of included studies and participants and summarise relevant characteristics of studies.	Space limitation required us to describe these details in the main text and Supplementary Information 2.1.
Synthesis of results	8	Present results for main outcomes, preferably indicating the number of included studies and participants for each. If meta-analysis was done, report the summary estimate and confidence/credible interval. If comparing groups, indicate the direction of the effect (i.e. which group is favoured).	Star rating is reported for each outcome, which is the culmination of the primary results. Further details and summary estimates are provided in the main text and Table 2.
**DISCUSSION**			
Limitations of evidence	9	Provide a brief summary of the limitations of the evidence included in the review (e.g. study risk of bias, inconsistency and imprecision).	Not in abstract. Covered in detail in the main text.
Interpretation	10	Provide a general interpretation of the results and important implications.	Yes
**OTHER**			
Funding	11	Specify the primary source of funding for the review.	Not in abstract due to space constraints but described in the "Acknowledgements".
Registration	12	Provide the register name and registration number.	No

Table S24: PRISMA 2020 Manuscript Checklist

Section and Topic	Item #	Checklist item	Location where item is reported
**TITLE**			
Title	1	Identify the report as a systematic review.	Not in the title due to focus on analytical methodology. Instead, this is explicitly stated in the Manuscript Abstract, Introduction, Discussion, and Methods.
**ABSTRACT**			
Abstract	2	See the PRISMA 2020 for Abstracts checklist.	Supplementary Table S23
**INTRODUCTION**			
Rationale	3	Describe the rationale for the review in the context of existing knowledge.	Introduction (lines 45-72)
Objectives	4	Provide an explicit statement of the objective(s) or question(s) the review addresses.	Introduction (lines 74-81)
**METHODS**			
Eligibility criteria	5	Specify the inclusion and exclusion criteria for the review and how studies were grouped for the syntheses.	Supplementary Information 1.2; Supplementary Information 3.1; Methods “Step 1” (lines 486-507)
Information sources	6	Specify all databases, registers, websites, organisations, reference lists and other sources searched or consulted to identify studies. Specify the date when each source was last searched or consulted.	Methods “Step 1” (lines 484-485); Supplementary Information 3.2; Introduction (lines 85-89; lines 116-123)
Search strategy	7	Present the full search strategies for all databases, registers and websites, including any filters and limits used.	Supplementary Information 1.1
Selection process	8	Specify the methods used to decide whether a study met the inclusion criteria of the review, including how many reviewers screened each record and each report retrieved, whether they worked independently, and if applicable, details of automation tools used in the process.	Methods “Step 1” (lines 488-507); Supplementary Information 3.2
Data collection process	9	Specify the methods used to collect data from reports, including how many reviewers collected data from each report, whether they worked independently, any processes for obtaining or confirming data from study investigators, and if applicable, details of automation tools used in the process.	Methods “Step 1” (lines 508-509; 516-520); Supplementary Information 1.5; Supplementary Information 3.2
Data items	10a	List and define all outcomes for which data were sought. Specify whether all results that were compatible with each outcome domain in each study were sought (e.g. for all measures, time points, analyses), and if not, the methods used to decide which results to collect.	Methods “Selecting the health outcomes of interest” (lines 466-481); Supplementary Information 1.3; Supplementary Information 1.5
	10b	List and define all other variables for which data were sought (e.g. participant and intervention characteristics, funding sources). Describe any assumptions made about any missing or unclear information.	Methods “Step 1” (lines 508-520); Supplementary Information 1.5
Study risk of bias assessment	11	Specify the methods used to assess risk of bias in the included studies, including details of the tool(s) used, how many reviewers assessed each study and whether they worked independently, and if applicable, details of automation tools used in the process.	Methods “Step 3” (lines 555-566); Supplementary Information 3.3
Effect measures	12	Specify for each outcome the effect measure(s) (e.g. risk ratio, mean difference) used in the synthesis or presentation of results.	Methods “Step 2” and “Step 6” (lines 523-527, 584-603); Table 2 caption
Synthesis methods	13a	Describe the processes used to decide which studies were eligible for each synthesis (e.g. tabulating the study intervention characteristics and comparing against the planned groups for each synthesis (item #5)).	Methods “Step 1” and “Step 2” (lines 493-507, 527-553); Supplementary Information 2.2
	13b	Describe any methods required to prepare the data for presentation or synthesis, such as handling of missing summary statistics, or data conversions.	Methods “Step 2” (lines 533-553); Supplementary Information 2.2; Supplementary Information 3.4
	13c	Describe any methods used to tabulate or visually display results of individual studies and syntheses.	Captions of Figures 1, 2, 4-7

	13d	Describe any methods used to synthesize results and provide a rationale for the choice(s). If meta-analysis was performed, describe the model(s), method(s) to identify the presence and extent of statistical heterogeneity, and software package(s) used.	Methods Overview (lines 419-442); Code availability statement; Supplementary Information 3.4
	13e	Describe any methods used to explore possible causes of heterogeneity among study results (e.g. subgroup analysis, meta-regression).	Methods "Step 4" (lines 569-578); Supplementary Information 3.4
	13f	Describe any sensitivity analyses conducted to assess robustness of the synthesized results.	Methods "Model validation" (lines 606-634); Supplementary Information 4
Reporting bias assessment	14	Describe any methods used to assess risk of bias due to missing results in a synthesis (arising from reporting biases).	Methods "Step 5" (lines 580-582)
Certainty assessment	15	Describe any methods used to assess certainty (or confidence) in the body of evidence for an outcome.	Methods "Step 6" (lines 584-603); Supplementary Information 3.4
**RESULTS**			
Study selection	16a	Describe the results of the search and selection process, from the number of records identified in the search to the number of studies included in the review, ideally using a flow diagram.	PRISMA flow diagrams (Supplementary Figures S1-S3)
	16b	Cite studies that might appear to meet the inclusion criteria, but which were excluded, and explain why they were excluded.	N/A
Study characteristics	17	Cite each included study and present its characteristics.	Supplementary Table S4
Risk of bias in studies	18	Present assessments of risk of bias for each included study.	Supplementary Information 3.3; Table 2
Results of individual studies	19	For all outcomes, present, for each study: (a) summary statistics for each group (where appropriate) and (b) an effect estimate and its precision (e.g. confidence/credible interval), ideally using structured tables or plots.	Figures 1, 4, 6, 7; Supplementary Table S5
Results of syntheses	20a	For each synthesis, briefly summarise the characteristics and risk of bias among contributing studies.	Table 2; Results (lines 130-136, 149-156, 170-180, 203-212, 230-238, 257-262, 275-279)
	20b	Present results of all statistical syntheses conducted. If meta-analysis was done, present for each the summary estimate and its precision (e.g. confidence/credible interval) and measures of statistical heterogeneity. If comparing groups, describe the direction of the effect.	Table 2; Supplementary Information 3.5; Supplementary Information 4; Results (lines 115-293)
	20c	Present results of all investigations of possible causes of heterogeneity among study results.	Table 2; Figure 3; Supplementary Information 3.5; Supplementary Information 4
	20d	Present results of all sensitivity analyses conducted to assess the robustness of the synthesized results.	Figure 3; Supplementary Information 4
Reporting biases	21	Present assessments of risk of bias due to missing results (arising from reporting biases) for each synthesis assessed.	Table 2; Supplementary Information 4; Results (lines 115-293)
Certainty of evidence	22	Present assessments of certainty (or confidence) in the body of evidence for each outcome assessed.	Table 2; Results (lines 115-293)
**DISCUSSION**			
Discussion	23a	Provide a general interpretation of the results in the context of other evidence.	Table 1; Discussion (lines 304-306, 316-322, 329-336)
	23b	Discuss any limitations of the evidence included in the review.	Table 1; Discussion (lines 360-398)
	23c	Discuss any limitations of the review processes used.	Table 1; Discussion (lines 369-371,385-398)
	23d	Discuss implications of the results for practice, policy, and future research.	Table 1; Introduction (lines 106-112); Discussion (lines 337-359, 409-415)
**OTHER INFORMATION**			
Registration and protocol	24a	Provide registration information for the review, including register name and registration number, or state that the review was not registered.	The entirety of the Global Burden of Diseases, Injuries, and Risk Factors Study has been registered and approved through the UW IRB. The systematic review was not registered separately. Methods (lines 449-451)
	24b	Indicate where the review protocol can be accessed, or state that a protocol was not prepared.	Methods (lines 451-452)
	24c	Describe and explain any amendments to information provided at registration or in the protocol.	N/A
Support	25	Describe sources of financial or non-financial support for the review, and the role of the funders or sponsors in the review.	Acknowledgments

Competing interests	26	Declare any competing interests of review authors.	Competing Interests Statement
Availability of data, code and other materials	27	Report which of the following are publicly available and where they can be found: template data collection forms; data extracted from included studies; data used for all analyses; analytic code; any other materials used in the review.	Data availability and Code availability statements; Supplementary Table S3

Figure S1. PRISMA 2020 flow diagram for chewing tobacco and head and neck cancers

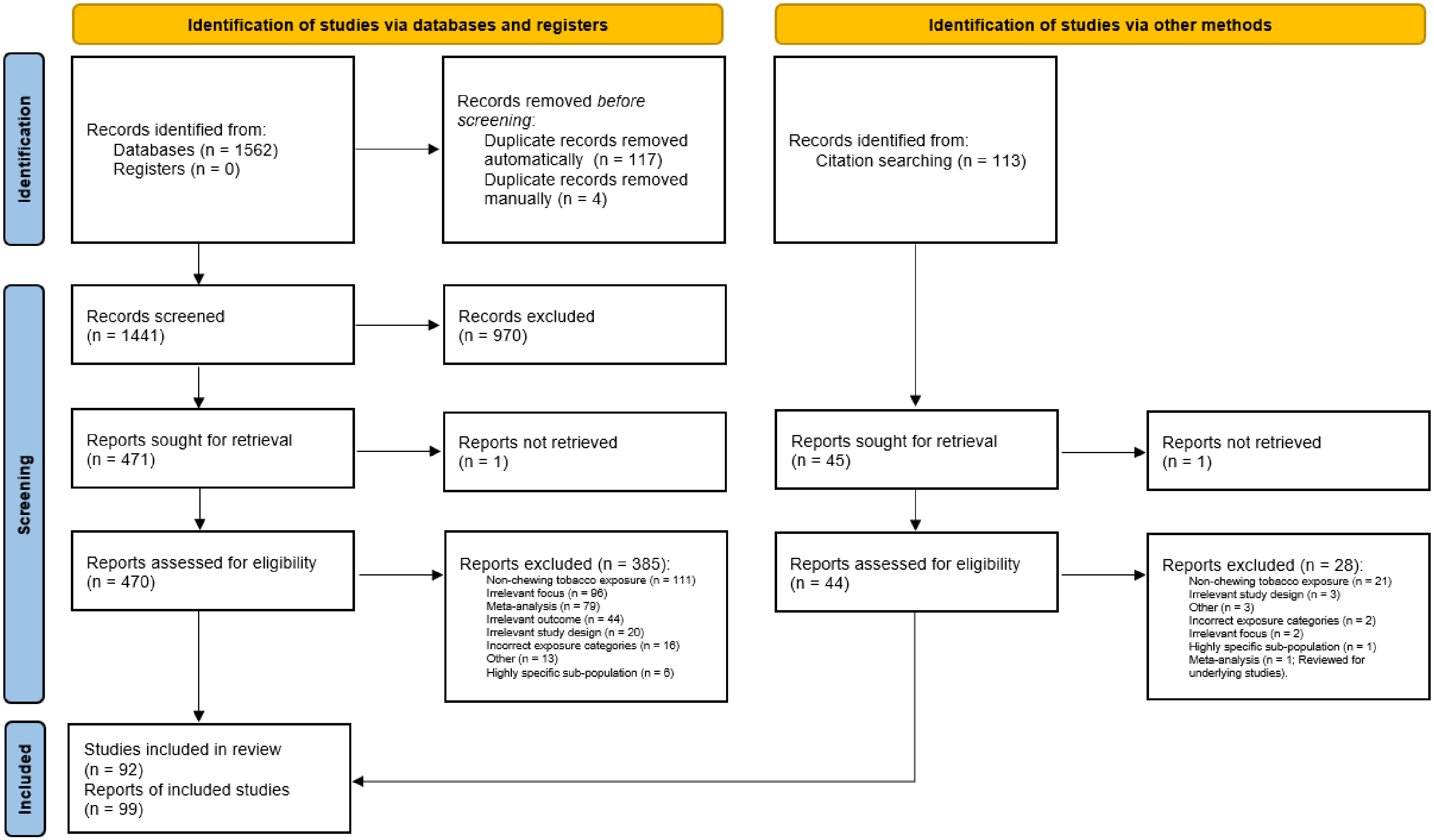

From: Page MJ, McKenzie JE, Bossuyt PM, Boutron I, Hoffmann TC, Mulrow CD, et al. The PRISMA 2020 statement: an updated guideline for reporting systematic reviews. BMJ 2021;372:n71. doi: 10.1136/bmj.n71. For more information, visit: http://www.prisma-statement.org/

Figure S2. PRISMA 2020 flow diagram for chewing tobacco and ischemic heart disease

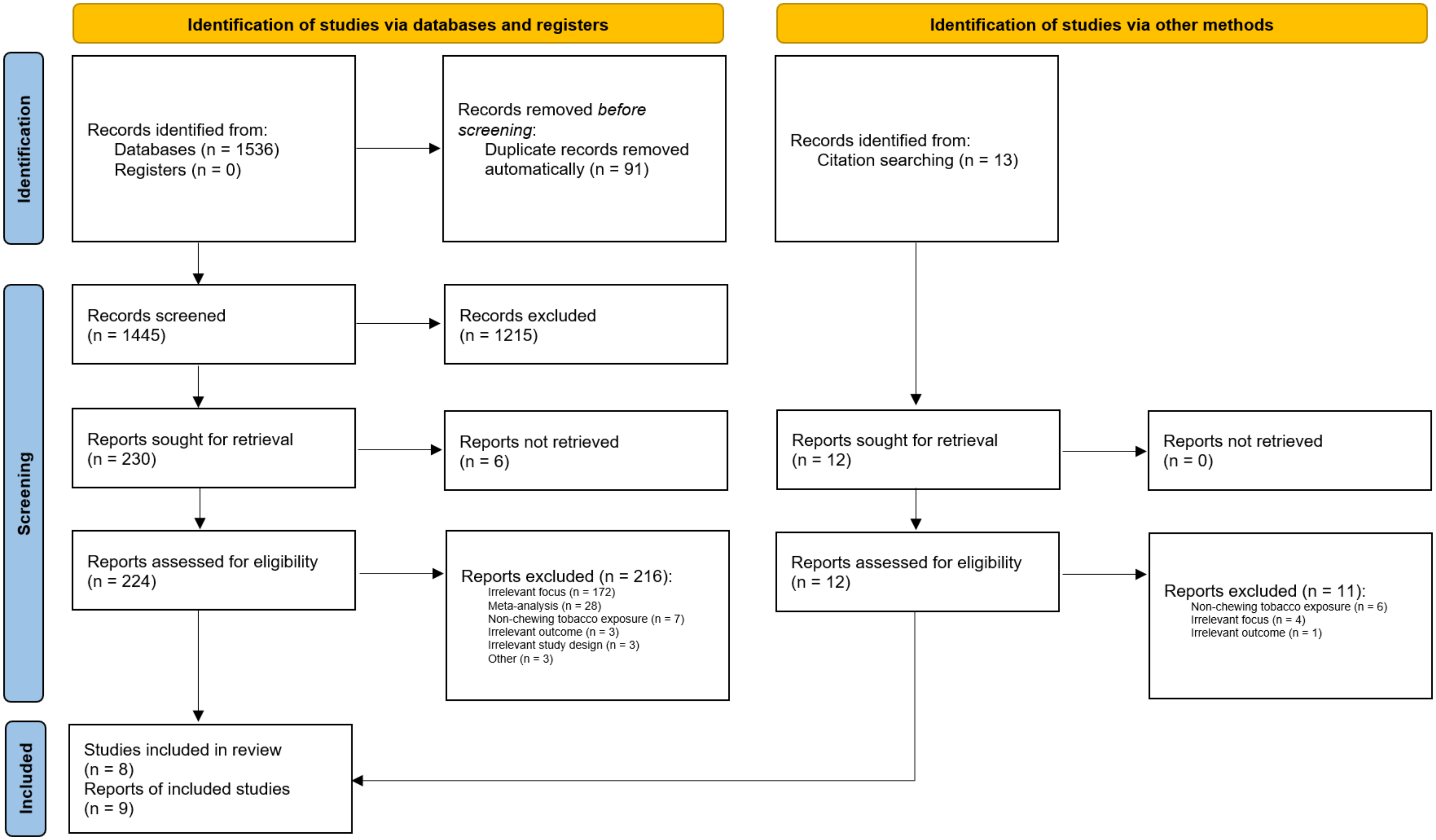

From: Page MJ, McKenzie JE, Bossuyt PM, Boutron I, Hoffmann TC, Mulrow CD, et al. The PRISMA 2020 statement: an updated guideline for reporting systematic reviews. BMJ 2021;372:n71. doi: 10.1136/bmj.n71. For more information, visit: http://www.prisma-statement.org/

Figure S3. PRISMA 2020 flow diagram for chewing tobacco and stroke

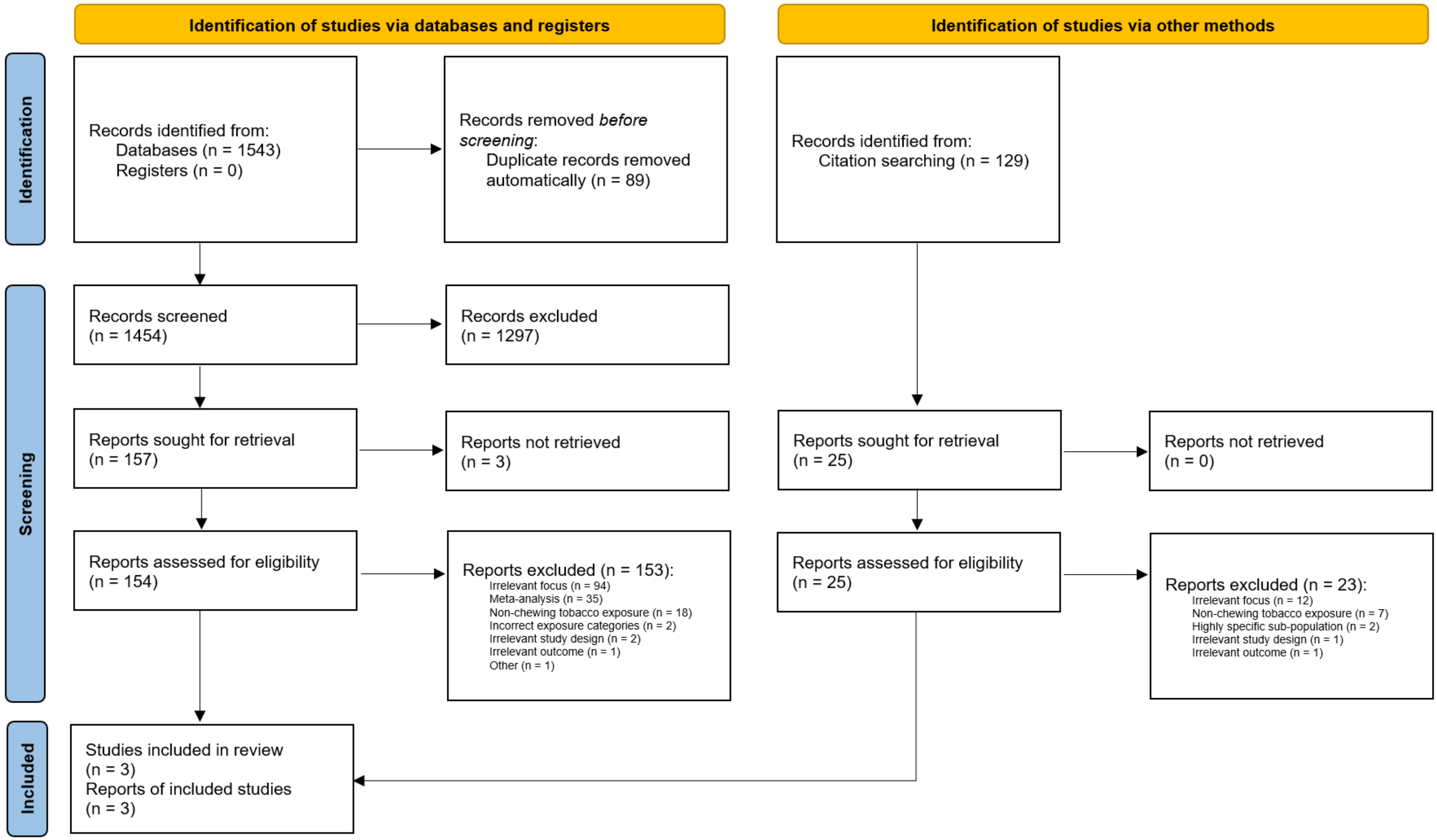

From: Page MJ, McKenzie JE, Bossuyt PM, Boutron I, Hoffmann TC, Mulrow CD, et al. The PRISMA 2020 statement: an updated guideline for reporting systematic reviews. BMJ 2021;372:n71. doi: 10.1136/bmj.n71. For more information, visit: http://www.prisma-statement.org/

Section 5.2: GATHER

Table S25: GATHER Checklist

Item #	Checklist item	Reported on page #
**Objectives and funding**		
**1**	Define the indicator(s), populations (including age, sex, and geographic entities), and time period(s) for which estimates were made.	Methods (p. 14)
**2**	List the funding sources for the work.	"Acknowledgements"
**Data Inputs**		
For all data inputs from multiple sources that are synthesized as part of the study:		
**3**	Describe how the data were identified and how the data were accessed.	Methods (p. 15); SI (p. 3-10, 44-45)
**4**	Specify the inclusion and exclusion criteria. Identify all ad-hoc exclusions.	Methods (p. 15); SI (p. 11)
**5**	Provide information on all included data sources and their main characteristics. For each data source used, report reference information or contact name/institution, population represented, data collection method, year(s) of data collection, sex and age range, diagnostic criteria or measurement method, and sample size, as relevant.	SI (p. 20-36); References
**6**	Identify and describe any categories of input data that have potentially important biases (e.g., based on characteristics listed in item 5).	Methods (p. 16); SI (p. 46-63)
For data inputs that contribute to the analysis but were not synthesized as part of the study:		
**7**	Describe and give sources for any other data inputs.	N/A
For all data inputs:		
**8**	Provide all data inputs in a file format from which data can be efficiently extracted (e.g., a spreadsheet rather than a PDF), including all relevant meta-data listed in item 5. For any data inputs that cannot be shared because of ethical or legal reasons, such as third-party ownership, provide a contact name or the name of the institution that retains the right to the data.	SI (p. 38-44, 79); Data availability statement (p. 18)
**Data analysis**		
**9**	Provide a conceptual overview of the data analysis method. A diagram may be helpful.	Main (p. 4); Methods (p. 13)
**10**	Provide a detailed description of all steps of the analysis, including mathematical formulae. This description should cover, as relevant, data cleaning, data pre-processing, data adjustments and weighting of data sources, and mathematical or statistical model(s).	Methods (p. 15-17); SI (p. 37-38, 63-64)
**11**	Describe how candidate models were evaluated and how the final model(s) were selected.	Methods (p. 17-18)
**12**	Provide the results of an evaluation of model performance, if done, as well as the results of any relevant sensitivity analysis.	Results (p. 5-9); SI (p. 64-78)
**13**	Describe methods for calculating uncertainty of the estimates. State which sources of uncertainty were, and were not, accounted for in the uncertainty analysis.	Methods (p. 16-17); SI (p. 64)
**14**	State how analytic or statistical source code used to generate estimates can be accessed.	Code availability statement (p. 18)
**Results and Discussion**		
**15**	Provide published estimates in a file format from which data can be efficiently extracted.	Table 2; Burden of Proof visualization tool (URL to be provided at resubmission)
**16**	Report a quantitative measure of the uncertainty of the estimates (e.g. uncertainty intervals).	Table 2; Results (p. 5-9)
**17**	Interpret results in light of existing evidence. If updating a previous set of estimates, describe the reasons for changes in estimates.	Discussion (p. 10-11)
**18**	Discuss limitations of the estimates. Include a discussion of any modelling assumptions or data limitations that affect interpretation of the estimates.	Table 1; Discussion (p. 12)

Section 6: Supplementary References

The references for underlying studies and literature cited in the main text are provided in the main manuscript. These references refer specifically to those that were cited in the methodology descriptions provided in this document, the Supplementary Information. They are all also cited in the main manuscript for proper reference.

1. Vos, T. *et al.* Global burden of 369 diseases and injuries in 204 countries and territories, 1990–2019: a systematic analysis for the Global Burden of Disease Study 2019. *The Lancet* **396**, 1204–1222 (2020).
2. Kendrick, P. J. *et al.* Spatial, temporal, and demographic patterns in prevalence of chewing tobacco use in 204 countries and territories, 1990–2019: a systematic analysis from the Global Burden of Disease Study 2019. *The Lancet Public Health* **6**, e482–e499 (2021).
3. Covidence - Better systematic review management. *Covidence* https://www.covidence.org/.
4. Aravkin, A. Y. *et al.* Reply to: Concerns about the Burden of Proof studies. *Nat Med* 1–2 (2023) doi:10.1038/s41591-023-02295-7.
5. Dai, X. *et al.* Health effects associated with smoking: a Burden of Proof study. *Nat Med* **28**, 2045–2055 (2022).
6. Lescinsky, H. *et al.* Health effects associated with consumption of unprocessed red meat: a Burden of Proof study. *Nat Med* **28**, 2075–2082 (2022).
7. Razo, C. *et al.* Effects of elevated systolic blood pressure on ischemic heart disease: a Burden of Proof study. *Nat Med* **28**, 2056–2065 (2022).
8. Stanaway, J. D. *et al.* Health effects associated with vegetable consumption: a Burden of Proof study. *Nat Med* **28**, 2066–2074 (2022).
9. Zheng, P. *et al.* The Burden of Proof studies: assessing the evidence of risk. *Nat Med* **28**, 2038–2044 (2022).

10. Boffetta P, Aagnes B, Weiderpass E, & Andersen A. Smokeless tobacco use and risk of cancer of the pancreas and other organs. *Int J Cancer* **114**, 992–5 (2005).
