# Supplementary material for: Health effects associated with chewing tobacco: a Burden of Proof study
Source: Nat Commun. 2024 Feb 5;15:1082. doi: 10.1038/s41467-024-45074-9 (PMC10844244; doi:10.1038/s41467-024-45074-9)
Supplement: Supplementary file 3 — Reporting Summary [file 41467_2024_45074_MOESM3_ESM.pdf]

# nature portfolio

Corresponding author(s): Emmanuela Gakidou, MSc, PhD

Last updated by author(s): 12/22/2023

# Reporting Summary

Nature Portfolio wishes to improve the reproducibility of the work that we publish. This form provides structure for consistency and transparency in reporting. For further information on Nature Portfolio policies, see our Editorial Policies and the Editorial Policy Checklist.

## Statistics

For all statistical analyses, confirm that the following items are present in the figure legend, table legend, main text, or Methods section.

| n/a | Confirmed | |
|---|---|---|
| ☐ | ☒ | The exact sample size (*n*) for each experimental group/condition, given as a discrete number and unit of measurement |
| ☐ | ☒ | A statement on whether measurements were taken from distinct samples or whether the same sample was measured repeatedly |
| ☒ | ☐ | The statistical test(s) used AND whether they are one- or two-sided<br>*Only common tests should be described solely by name; describe more complex techniques in the Methods section.* |
| ☐ | ☒ | A description of all covariates tested |
| ☐ | ☒ | A description of any assumptions or corrections, such as tests of normality and adjustment for multiple comparisons |
| ☐ | ☒ | A full description of the statistical parameters including central tendency (e.g. means) or other basic estimates (e.g. regression coefficient) AND variation (e.g. standard deviation) or associated estimates of uncertainty (e.g. confidence intervals) |
| ☒ | ☐ | For null hypothesis testing, the test statistic (e.g. *F*, *t*, *r*) with confidence intervals, effect sizes, degrees of freedom and *P* value noted<br>*Give P values as exact values whenever suitable.* |
| ☐ | ☒ | For Bayesian analysis, information on the choice of priors and Markov chain Monte Carlo settings |
| ☒ | ☐ | For hierarchical and complex designs, identification of the appropriate level for tests and full reporting of outcomes |
| ☐ | ☒ | Estimates of effect sizes (e.g. Cohen's *d*, Pearson's *r*), indicating how they were calculated |

*Our web collection on statistics for biologists contains articles on many of the points above.*

## Software and code

Policy information about availability of computer code

| | |
|---|---|
| Data collection | No primary data was collected for this analysis. Secondary data was collected through systematic reviews that are described in detail in the manuscript. The systematic reviews were managed in Covidence, a systematic review management tool linked here: https://www.covidence.org/. No other software was used in data collection. |
| Data analysis | Analyses were carried out using R version 4.0.5 and Python version 3.10.9. The code used, including all relevant packages, are publicly available at https://github.com/ihmeuw-msca/burden-of-proof. |

For manuscripts utilizing custom algorithms or software that are central to the research but not yet described in published literature, software must be made available to editors and reviewers. We strongly encourage code deposition in a community repository (e.g. GitHub). See the Nature Portfolio guidelines for submitting code & software for further information.

## Data

Policy information about availability of data

All manuscripts must include a data availability statement. This statement should provide the following information, where applicable:

- Accession codes, unique identifiers, or web links for publicly available datasets
- A description of any restrictions on data availability
- For clinical datasets or third party data, please ensure that the statement adheres to our policy

The findings from this study are supported by data extracted from published literature. Details on data sources can be found on the Burden of Proof visualization tool (https://vizhub.healthdata.org/burden-of-proof/). Study characteristics and citations for all input data used in the analyses are also provided in the

Supplementary Information.

## Research involving human participants, their data, or biological material

Policy information about studies with human participants or human data. See also policy information about sex, gender (identity/presentation), and sexual orientation and race, ethnicity and racism.

| | |
|---|---|
| Reporting on sex and gender | As a meta-analysis of peer-reviewed literature, this study does not involve primary data collection of human research participants. Our estimates are not specific to specific demographic populations, including by sex or gender, and we did not exclude studies that did not report sex- or gender-specific estimates. If a study only reported information on disaggregated effect sizes, that data would be used. However, due to heterogeneity in the way underlying studies collected and reported on sex or gender, we did not distinguish between the two concepts in our extractions and refer to them as sex-specific data. While our primary analysis did not consider sex-specific differences, we conducted a sensitivity analysis with sex-specific data for males and for females, and we did not find substantially different results by sex. Furthermore, the limitations and scarcity of sex-specific data restricted our ability to even perform this sensitivity analysis. |
| Reporting on race, ethnicity, or other socially relevant groupings | Our analysis did not limit included literature to a specific geographic region or country, so groupings that may have been described as socially relevant differed substantially across our included studies. We extracted whether or not a study effect size was adjusted for race/ethnicity or other such groupings, like urban/rural. This information was used to derive a cascading covariate for an effect size's degree of adjustment, which is presented in the Supplementary Information, but is not reported in detail given the large variation of included studies. |
| Population characteristics | This study is a meta-analysis of estimates published in peer-reviewed literature, so it does not involve direct interaction with a human population. Included studies were not limited by geography, age, sex, or other demographic characteristics except for study populations that were defined by clinical characteristics that would affect the generalizability of the findings, such as populations of diabetes patients or cancer survivors. Other characteristics of the populations of the included studies were extracted, per the data extraction template provided in the Supplementary Information. |
| Recruitment | Our analysis is a meta-analysis of peer-reviewed literature, so it does not involve primary data collection or recruitment of individual participants. |
| Ethics oversight | This study was approved by the University of Washington IRB Committee (study #9060) as a component of the Global Burden of Disease, Injuries, and Risk Factors (GBD) study. |

Note that full information on the approval of the study protocol must also be provided in the manuscript.

# Field-specific reporting

Please select the one below that is the best fit for your research. If you are not sure, read the appropriate sections before making your selection.

☒ Life sciences ☐ Behavioural & social sciences ☐ Ecological, evolutionary & environmental sciences

For a reference copy of the document with all sections, see nature.com/documents/nr-reporting-summary-flat.pdf

# Life sciences study design

All studies must disclose on these points even when the disclosure is negative.

| | |
|---|---|
| Sample size | The number of studies included was determined through three systematic reviews in which studies underwent multiple rounds of screening and the underlying studies of meta-analyses were reviewed. Extracted data points were used for each included study, so the sample size for each analysis was determined by the total number of available and relevant studies identified in the systematic reviews. No power calculation was done as a result because the present analyses leverage all of the available and relevant studies identified. The sample size of each included study can be found in Supplementary Table S4, the number of included studies per primary model is reported in Table 2, and the number of included studies in each sensitivity analysis is reported in Supplementary Tables S13-S19. |
| Data exclusions | In the systematic reviews, we excluded studies on the basis of pre-determined exclusion criteria. It is described in detail in Supplementary Information Section 1.2. In brief, we excluded studies that did not use a cohort or case-control study design, used a non-chewing tobacco exposure, involved a highly-specific sub-population, had an irrelevant focus, used an irrelevant outcome, reported on Global Burden of Disease, Injuries, and Risk Factors (GBD) study results, and did not have data to calculate/extract binary effect sizes. |
| Replication | This is a meta-analysis of existing published literature. The data and code have all been made available, and the data was identified through common large databases. It could theoretically be replicated. |
| Randomization | This study is a meta-analysis of existing literature. Thus, there were no experimental groups and no need for randomization. |
| Blinding | Blinding was not relevant to this study, as it was a meta-analysis using existing secondary data. |

# Reporting for specific materials, systems and methods

We require information from authors about some types of materials, experimental systems and methods used in many studies. Here, indicate whether each material, system or method listed is relevant to your study. If you are not sure if a list item applies to your research, read the appropriate section before selecting a response.

## Materials & experimental systems

| n/a | Involved in the study |
| --- | --- |
| ☒ | ☐ Antibodies |
| ☒ | ☐ Eukaryotic cell lines |
| ☒ | ☐ Palaeontology and archaeology |
| ☒ | ☐ Animals and other organisms |
| ☒ | ☐ Clinical data |
| ☒ | ☐ Dual use research of concern |
| ☒ | ☐ Plants |

## Methods

| n/a | Involved in the study |
| --- | --- |
| ☒ | ☐ ChIP-seq |
| ☒ | ☐ Flow cytometry |
| ☒ | ☐ MRI-based neuroimaging |
